# One hundred years of benzotropone chemistry

**DOI:** 10.3762/bjoc.14.98

**Published:** 2018-05-23

**Authors:** Arif Dastan, Haydar Kilic, Nurullah Saracoglu

**Affiliations:** 1 Department of Chemistry, Science Faculty, Atatürk University, 25240, Erzurum, Turkey; 2 Oltu Vocational Training School, Atatürk University, 25400, Erzurum, Turkey; 3 East Anotolia High Technology Application and Research Center, Atatürk University, 25240, Erzurum, Turkey

**Keywords:** benzotropolone, benzotropone, dibenzotropone, halobenzotropolone, halobenzotropone, tribenzotropone, tropone

## Abstract

This review focuses on the chemistry of benzo-annulated tropones and tropolones reported since the beginning of the 20th century, which are currently used as tools by the synthetic and biological communities.

## Review

### 1. Introduction

**Scheme 1:**
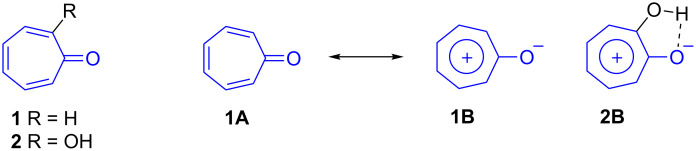
Tropone (**1**), tropolone (**2**) and their resonance structures.

Tropone (**1**) and tropolone (**2**) have fascinated organic chemists for well over one hundred years. The carbocycles **1** and **2** are a special variety of organic compounds and represent a nonbenzenoid type of aromatic system (Scheme 1). Their dipolar resonance structures such as tropylium oxide form **1B** and **2B** have been reported to provide a Hückel sextet of electrons that is necessary for aromaticity (Scheme 1) [1–9].

**Figure 1:**
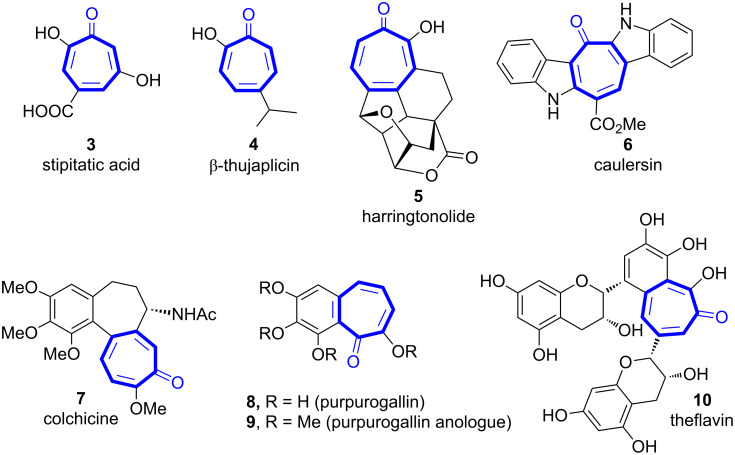
Natural products containing a tropone nucleus.

The tropone core is the ubiquitous structural motif in the alkaloid colchicine and in a number of other natural compounds that have shown a highly diverse range of biological activity [1–9], like the inhibitory activity of inositol monophosphatase [10–11], antitumor [12], antibiotic [12–13], and antibacterial activity [14] and lipoxygenase inhibitor activity [14–16]. Troponoids **3**–**10** have been reported in the literature in a number of natural forms (Figure 1) [1–8]. These compounds have a structural class spacing from the simple monocyclic tropones, such as the potent antifungal and antibiotic monoterpene β-thujaplicin (**4**) [17–23] (isolated from the heartwood and essential oils of trees of the family *Cupressaceae*), to complex macrocyclic analogues, such as harringtonolide (**5**) [24–26], which was found to have antineoplastic and antiviral properties, and caulersin (**6**) [26], which is a biologically active natural tropone fused to indole rings (Figure 1). Benzo-annulated cycloheptanones (as colchicine [8], allocolchicine), benzo[7]annulenones, or benzotropones (as purpurogallin) and their analogues are present in a great variety of pharmacologically relevant natural products [27–29]. Colchicine (**7**, from *Colchicum autumnale*) is a medication most commonly used to treat gout and familial Mediterranean fever (Figure 1) [30]. Colchicine and its analogues are potent microtubule-polymerizing agents and they inhibit growth of human cancer cell lines and show antimitotic activity [31–36]. Purpurogallin (**8**), which is biogenetically produced by oxidation of pyrogallol, and its analogues (like **9**) are natural pigments (Figure 1) [37–44]. Theaflavin (**10**) and its derivatives, named theaflavins, are antioxidant benzotropones that are formed by the enzymatic oxidation of black tea and have been found to have numerous biological activities such as antipathogenic and anticancer activity, and they prevent heart disease, hypertension, and diabetes (Figure 1) [39,43]. Because of the pharmacological relevance of benzotropone analogues, the development of new and efficient synthetic methods is one of the major goals for future research in chemistry. Perhaps most importantly, the continued interest in troponoids systems originates from the fact that such compounds can be used as both building blocks and starting materials in the synthesis of complex natural products [1–9].

To date, the chemistry of tropone (**1**) and tropolones **2** has been reviewed [1–9], but there have been no surveys covering benzotropones and benzotropolenes completely. Tang’s group published a recent review limited to the synthesis of naturally occurring tropones and tropolones [9]. In addition to this, chemistry of dibenzosuberenone, which is one of the dibenzotropone isomers, has already reviewed by us [45].

**Figure 2:**
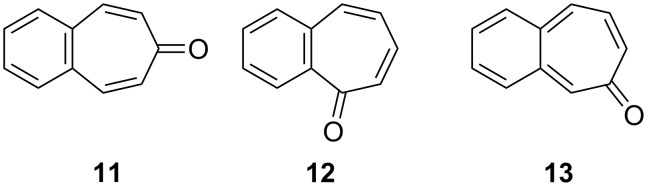
Possible isomers **11**–**13** of benzotropone.

There are three possible benzotropone isomers: 4,5-benzotropone (**11**), 2,3-benzotropone (**12**), and 3,4-benzotropone (**13,** Figure 2). The present review focuses on the chemistry of parent benzotropones and their hydroxy analogues (benzotropolones) in the hundred years from the beginning to the present day, because these classes of molecules still attract noticeable attention from the synthetic and biological communities due to emerging reports of their interesting chemical structures and potential biological activities. Historically, many efforts have been devoted to the chemistry of benzotropones/benzotropolones and a plethora of benzotropone-type molecules have been produced over 100 years. Furthermore, the scope of this review includes the chemistry of halo-benzotropones, halo-benzotropolones, dibenzotropones, dibenzotropolones, tribenzotropone, and tropoquinones in addition to parent benzotropones and benzotropolones. The numerous functionalized benzotroponoids are excluded from the review.

### 2. Chemistry of 4,5-benzotropone (**11**)

Several research studies have been reported on the synthesis and properties of 4,5-benzotropones since they were first prepared by Thiele and Weitz nearly a century ago [46–47]. Similar approaches of this method was independently studied by Cook [48] and Föhlisch [49] groups. In 1975, the crystal and molecular structure of 4,5-benzotropone (**11**) was determined by Hata’s group [50]. X-ray diffraction analysis showed that the molecule is approximately planar and the bond alternation in the seven-membered ring and C=O bond length support satisfactory aromaticity.

#### 2.1. Synthesis of 4,5-benzotropone (**11**)

**Scheme 2:**
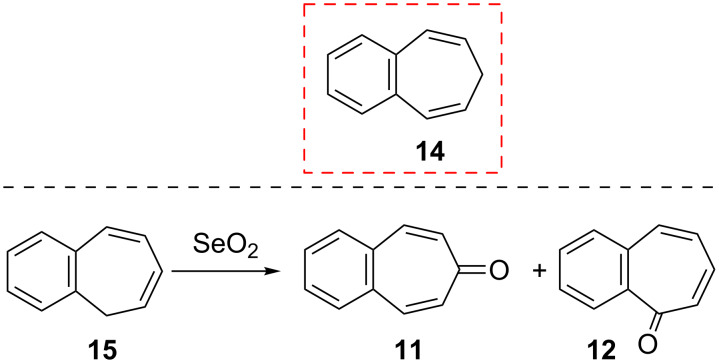
Synthesis of benzotropones **11** and **12**.

**2.1.1. Oxidation of benzo[7]annulenes:** 4,5-Benzotropone (**11**) was synthesized for the first time via oxidation with selenium dioxide of 7*H*-benzo[7]annulene (**14**, Scheme 2) [39–45]. Furthermore, the direct oxidation of 5*H*-benzo[7]annulene to benzotropones was examined by Srivastava and Dev [37]. The selenium dioxide oxidation of 5*H*-benzo[7]annulene (**15**) furnished not only 4,5-benzotropone (**11**; 27%) but also 2,3-benzotropone (**12**; 13%, Scheme 2).

**Scheme 3:**
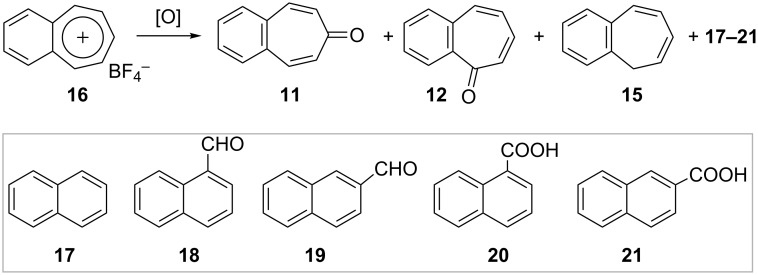
Oxidation products of benzotropylium fluoroborate (**16**).

Pomerantz and Swei [51] investigated the oxidation of benzotropylium cation **16** with several oxidants. The oxidants used and results obtained are summarized in Table 1 and Scheme 3. Oxidation of benzotropylium fluoroborate (**16**) with Na_2_O_2_ and KO_2_ gives benzotropones as the major products, whereas oxidation with *m*-chloroperoxybenzoic acid (*m*-CPBA) produces a small amount of ring-contracted naphthaldehydes along with benzotropones. The oxidation with Na_2_O_2_ gives slightly higher amounts of benzotropones than of naphthaldehydes. As shown in Table 1, the most suitable reaction conditions to obtain 4,5-benzotropone (**11**) with the Pomerantz and Swei procedure include Na_2_O_2_/Me_2_SO.

**Table 1:** The oxidation of benzotropylium fluoroborate (**16**).

reagent/solvent	products/absolute yields							

	**11**	**12**	**15**	**17**	**18**	**19**	**20**	**21**
	
Na_2_O_2_/Me_2_SO	56	4	4	–	trace	trace	–	–
Na_2_O_2_/CH_2_Cl_2_	45	14	15	–	trace	trace	–	–
KO_2_/DMF	25	4	4	–	trace	trace	–	–
Na_2_O_2_ (90%)/THF	3	13	0.5	0.9	2	3	5	13
Na_2_O_2_ (30%)/THF	9	13	1.2	2	9	13	2	4
*m*-CPBA/CH_2_Cl_2_	17	5	10	0.7	3	trace	–	–
*t*-BPA/CH_2_Cl_2_	6	0.2	0.4	1	trace	trace	trace	8

**Scheme 4:**
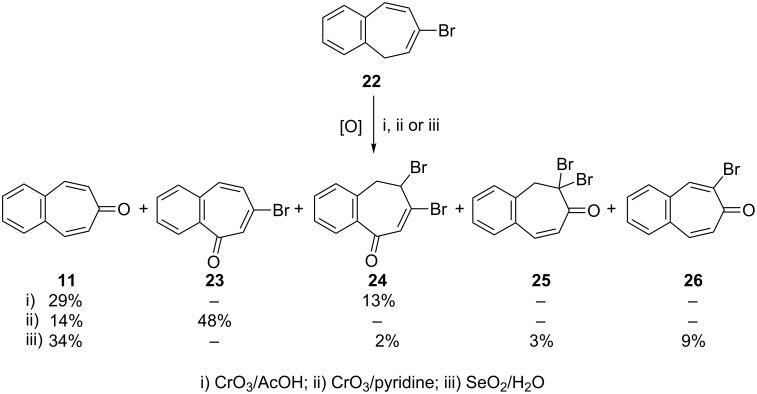
Oxidation of 7-bromo-5*H*-benzo[7]annulene (**22**).

Mechanistic and synthetic aspects of the reaction of 7-bromo-5*H*-benzo[7]annulene (**22**) with CrO_3_ and SeO_2_ as oxidation reagents were studied (Scheme 4) [52]. All reactions provided 4,5-benzotropone (**11**) in addition to a few benzotroponoid compounds **23**–**26**, the structures of which were determined by means of spectral data and chemical transformations. It is deemed that the dibromides **24** and **25** are the result of the addition of HBr, which is formed under the reaction conditions.

**Scheme 5:**
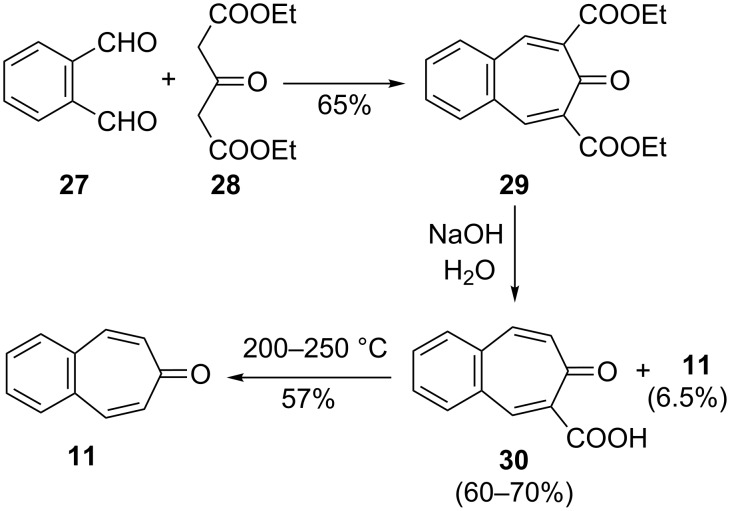
Synthesis of 4,5-benzotropone (**11**) using *o*-phthalaldehyde (**27**).

**2.1.2. Multistep synthesis of 4,5-benzotropone (11):** The first multistep synthesis for 4,5-benzotropone (**11**) is the original procedure described by Thiele, Schneider, and Weitz, which involves the condensation of *o*-phthalaldehyde (**27**) with diethyl 1,3-acetonedicarboxylate (**28**), followed by hydrolysis and decarboxylation steps [46–47] (Scheme 5). Similar syntheses were made by Cook’s [48] and Föhlisch’s [49] groups. Nevertheless, for performing large-scale synthesis, the cost of **27** make deterrent and an autoclave environment at 200–250 °C for final stage are an unattractive feature (Scheme 5).

**Scheme 6:**
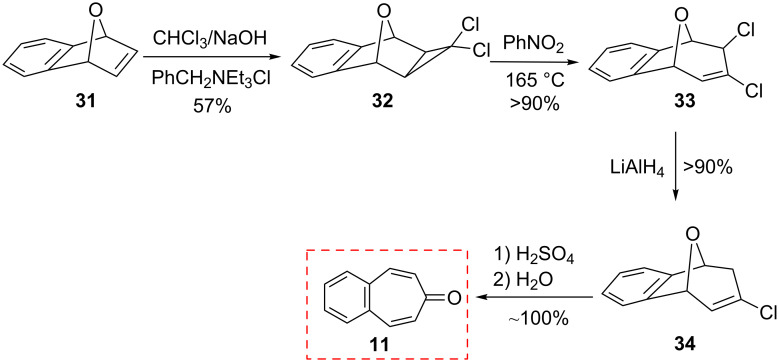
Synthesis of 4,5-benzotropone (**11**) starting from oxobenzonorbornadiene **31**.

Ranken’s group reported a novel synthetic route to 4,5-benzotropone (**11**) via an acid-catalyzed bridge cleavage reaction of 7-chloro-6,9-dihydro-5*H*-5,9-epoxybenzo[7]annulene (**34**) (Scheme 6) [53]. Transformation of adduct **31** to **11** starts with the synthesis of the stable cyclopropanoid tricyclic **32** from the reaction of the 7-oxabenzonorbornadiene **31** with dichlorocarbene, generated by the phase-transfer method. The thermolysis of dichloride **32** in nitrobenzene at 165 °C resulted in the formation of ring-expanded product **33**. After the reduction of the allylic position with LiAlH_4_, the treatment of monochloride **34** with concentrated sulfuric acid in ice water afforded a quantitative yield of 4,5-benzotropone (**11**).

**Scheme 7:**
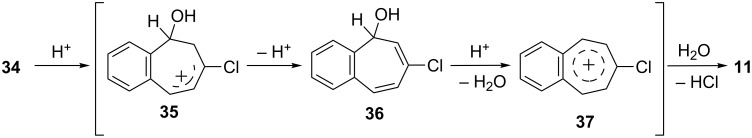
Acid-catalyzed cleavage of oxo-bridge of **34**.

The above researchers also proposed a mechanism for the formation of **11** from **31** as shown in Scheme 7. The acid-catalyzed cleavage of the oxo-bridge of **34** gives benzylic carbocation **35**. Consequently, after deprotonation and dehydration, chloro benzotropilium cation **37** undergoes hydrolysis to give 4,5-benzotropone (**11**) in aqueous reaction media.

**Scheme 8:**
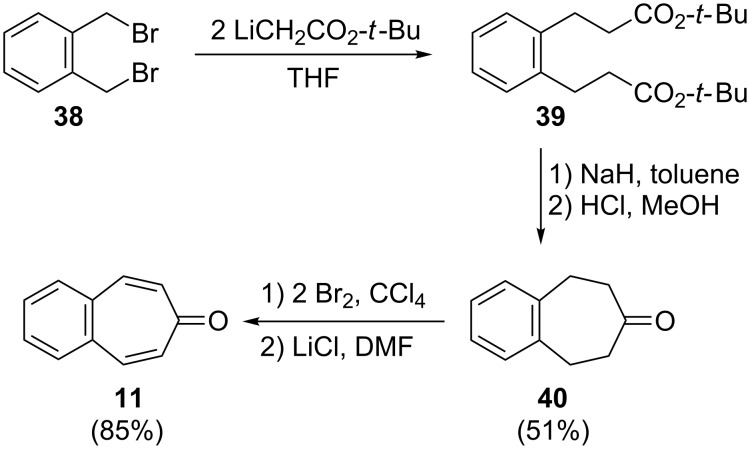
Synthesis of 4,5-benzotropone (**11**) from *o*-xylylene dibromide (**38**).

Using *o*-xylylene dibromide (**38**) as starting material, Ewing and Paquette designed and synthesized benzotropone **11** by an especially reliable route [54]. For this purpose, bisalkylation of *o*-xylylene dibromide (**38**) with *tert*-butyl lithioacetate (Rathke's salt) and subsequent Dieckmann cyclization provided simple access to **40** in 51% overall yield (Scheme 8). After bromination of **40** with molecular bromine in carbon tetrachloride, direct dehydrobromination with lithium chloride in dimethylformamide gave **11** in 85% isolated yield.

**Scheme 9:**
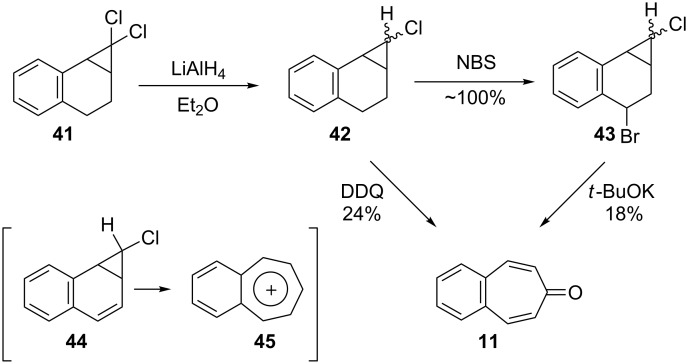
Synthesis of 4,5-benzotropone (**11**) via the carbene adduct **41**.

Müller’s group reported an alternative synthesis for **11** starting from the carbene adduct **41** over two or three steps [55]. Firstly, dichloride **41** was reduced with LiAlH_4_ in ether to give the monochloride **42**. The reaction of **42** with DDQ produced 4,5-benzotropone (**11**) in 24% yield together with 28% of starting material. The key step for **11** from **42** is the electrocyclic ring expansion of dehydrogenation product **44** to the benzotropylium ion **45**. Secondly, **11** is obtained in 18% yield after benzylic bromination of **42** with NBS, followed by in situ elimination reaction of the labile bromide **43** mediated by *t*-BuOK (Scheme 9).

**Scheme 10:**
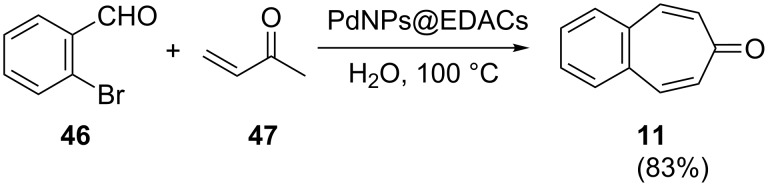
Heck coupling strategy for the synthesis of **11**.

Palladium-catalyzed C–C bond-formation reactions such as Heck and Sonogashira couplings are employed in a wide variety of areas in organic chemistry [56–57]. Recently, Shaabani’s group synthesized and characterized palladium nanoparticles supported on ethylenediamine-functionalized cellulose (PdNPs@EDACs) as a novel bio-supported catalyst for Heck and Sonogashira couplings in water [58]. Shaabani’s group reported the efficient synthesis of benzotropone **11** in a good isolated yield (83%) via PdNPs@EDACs-catalyzed Heck coupling and intramolecular condensation of 2-bromobenzaldehyde (**46**) and methyl vinyl ketone (**47**) (Scheme 10) [58].

#### 2.2. Reactions of 4,5-benzotropone (**11**)

**Scheme 11:**
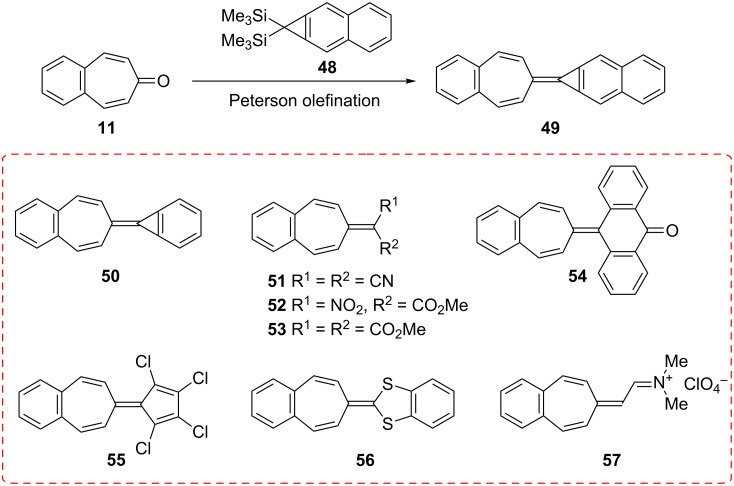
Synthesis of benzofulvalenes via carbonyl group of 4,5-benzotropone (**11**).

**2.2.1. Reactions via the carbonyl group:** Fulvalenes are typical cross-conjugated carbocyclic unsaturated compounds and are of theoretical and synthetic interest [59]. Several researchers have studied the synthesis of benzofulvalenes via the carbonyl group of 4,5-benzotropone (**11**) (Scheme 11). Halton’s group applied the Peterson olefination reaction to the synthesis of benzofulvalanes **49** and **50** from the reaction of 4,5-benzotropone (**11**) with corresponding cyclopropanes [60–61]. While the reaction of 4,5-benzotropone (**11**) with malononitrile afforded 8,8-dicyano-3,4-benzoheptafulvalene (**51**) [62], the condensation of 4,5-benzotropone (**11**) with dimethyl malonate and its nitro analogue gave benzoheptafulvalene derivatives **52** and **53** [50,63]. The condensation of 4,5-benzotropone (**11**) and anthrone (10*H*-anthracen-9-one) also afforded 4,5-benzo-tropyliden-anthron **54** in 65% yield [63]. Kitahara reported the synthesis of 1,2,3,4-tetrachloro-7,8-benzosesquifulvalene **55** via condensation of 1,2,3,4-tetrachlorocyclopentadiene and 4,5-benzotropone (**11**) [64]. The thia-heptafulvalene **56** was synthesized by Wittig–Horner reaction of 4,5-benzotropone (**11**) with 2-diethoxyphosphinyl-1,3-benzodithiole [59]. The reactants used for the synthesis of benzoheptafulvalene derivate **57** were *N*,*N*-dimethylformamide, methyl iodide, 4,5-benzotropone (**11**), and NaClO_4_ [65].

**Figure 3:**
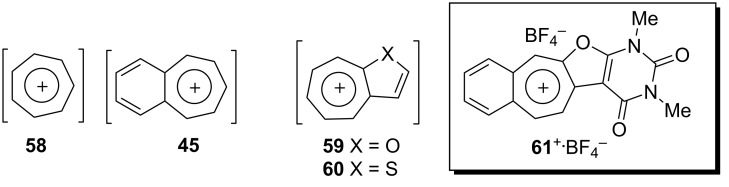
Some cycloheptatrienylium cations.

**Scheme 12:**
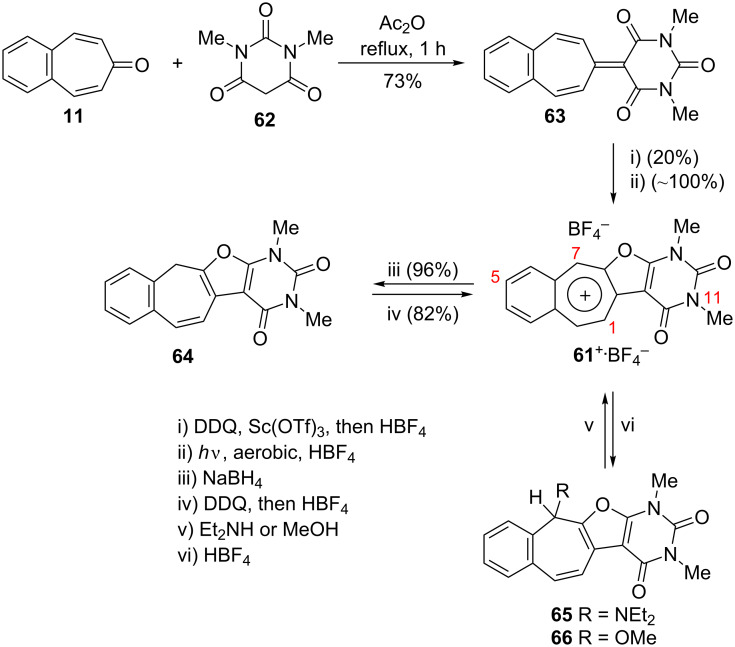
Synthesis of condensation product **63** and its subsequent oxidative cyclization products.

As polycyclic conjugated π systems can endow new properties to the original π system, conjugated systems are important in terms of both theoretical and experimental aspects. Nitta’s group extensively studied the synthesis and structural and chemical properties of a new kind of cycloheptatrienylium ions using aromatic π systems (Figure 3) [66–70]. In this context, Nitta and colleagues reported the synthesis, properties, and oxidizing ability of the novel **61^+.^**BF_4_^−^ [71]. A condensation reaction of 4,5-benzotropone (**11**) with dimethyl barbituric acid (**62**) and subsequent oxidative cyclization reaction using DDQ-Sc(OTf)_3_ or photoirradiation under aerobic conditions afforded **61^+.^**BF_4_^−^ (Scheme 12). The p*K*_R+_ value and reduction potential of the cation **61** were studied. The relative stability of a carbocation can be expressed by the p*K*_R+_ value, which is the affinity of the carbocation toward hydroxide ions. The p*K*_R+_ value for cation **61** was determined to be 4.7 spectrophotometrically. The reduction potential of the cation **61** was determined as −0.46 and −1.07 V by cyclic voltammetry in acetonitrile. The oxidizing ability toward alcohols of **61^+.^**BF_4_**^−^** in the auto-recycling process was also reported. However, to test the reactivity, the reactions of **61^+.^**BF_4_^−^ with a nucleophile such as NaBH_4_, diethylamine, and methanol were carried out to afford 7-adducts **64**–**66**. Compound **64** was oxidized by DDQ to regenerate **61^+.^**BF_4_^−^ in good yield, whereas the treatment with 42% aq HBF_4_ of the compounds **65** and **66** regenerated **61^+.^**BF_4_^−^ in good yields.

**Figure 4:**
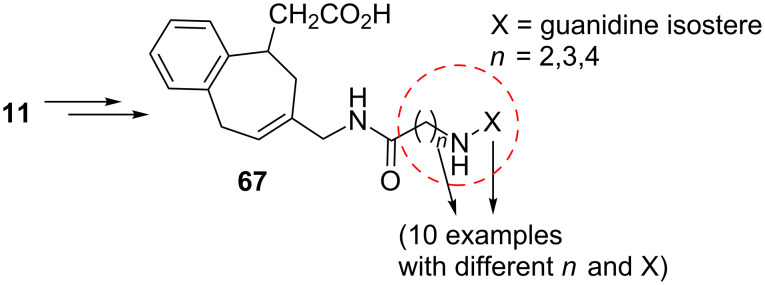
A novel series of benzo[7]annulenes prepared from 4,5-benzotropone (**11**).

**Scheme 13:**
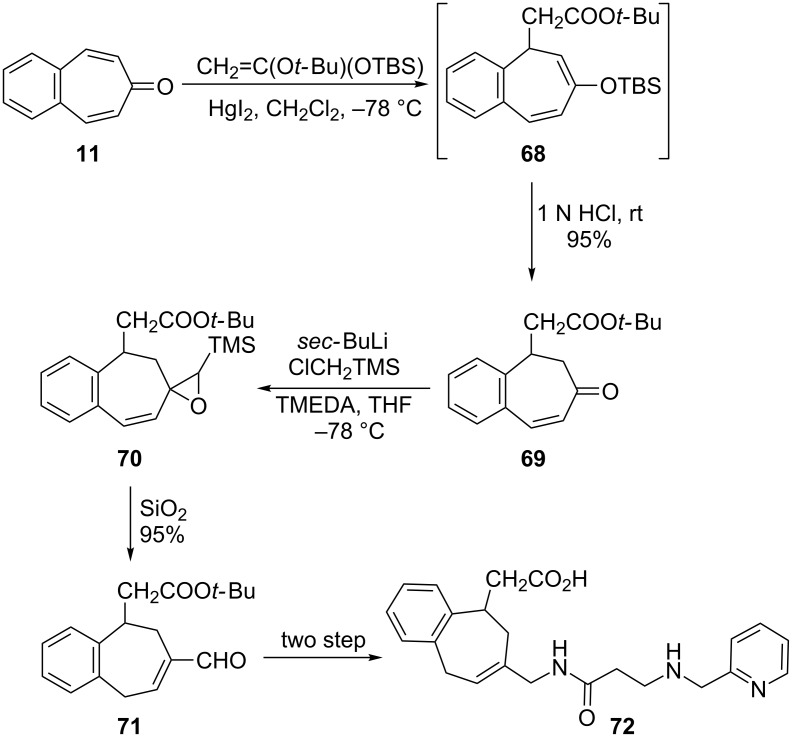
Preparation of substituted benzo[7]annulene **72** using the Mukaiyama-Michael reaction.

Integrins are transmembrane α/β heterodimers that bind to extracellular matrix ligands, cell-surface ligands, and soluble ligands [72]. Perron-Sierra’s group prepared substituted benzo[7]annulenes as a novel series of potent and specific α_v_ integrin antagonists starting from 4,5-benzotropone (**11**) (Figure 4 and Scheme 13) [73].

TBS-enol ether intermediate **68** was first formed by the Mukaiyama–Michael reaction of *O*-silyl ketene acetal to 4,5-benzotropone (**11**) at low temperature in the presence of catalytic mercury iodide; it is a critical step for the formation of the compound **72**. Hydrolysis of intermediate **68** led to ketone **69** in high yield containing an acetic acid residue β to the carbonyl group. A sequence of the one-carbon homologation of ketone **69** is followed by isomerization into the α,β-unsaturated aldehyde **71**. A series of reductive amination and amidation reactions then led to the formation of the targeted substituted benzo[7]annulene **72** (Scheme 13). Moreover, the structure–activity study revealed that some of the compounds showed nano- to subnanomolar IC_50_ values on α_v_β_3_ and α_v_β_5_ integrins.

**Figure 5:**
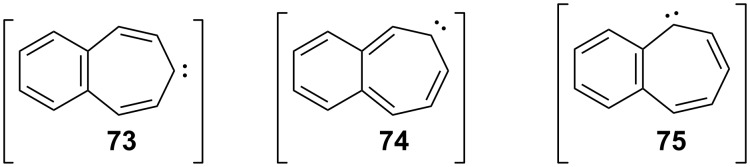
Possible benzo[7]annulenylidenes **73**–**75**.

**Scheme 14:**
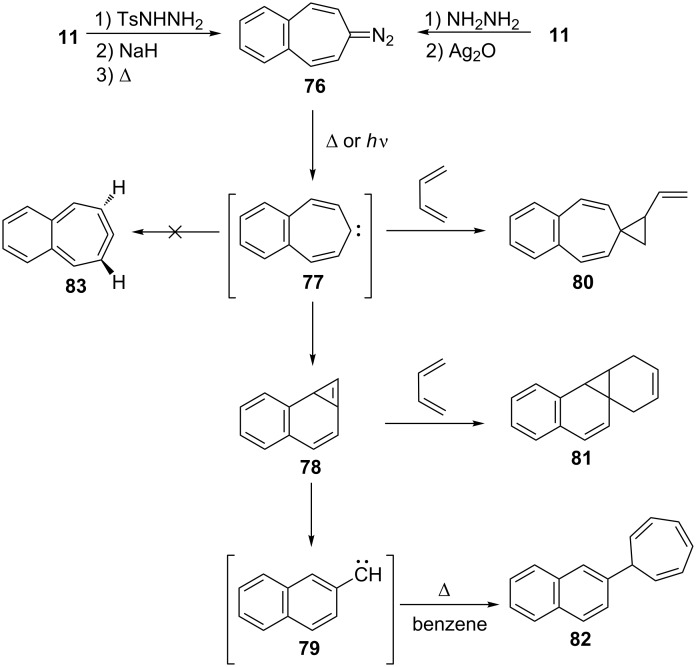
Thermal and photochemical decomposition of 7-diazo-7*H*-benzo[7]annulene (**76**) and the trapping of intermediates **77**–**79**.

Benzo[7]annulenylidenes **73**–**75** and their rearrangements have attracted much interest due to their thermal and photochemical transformations (Figure 5) [74–76]. For the first time, Jones reported the chemical trapping of thermal and photochemical decomposition of the tosylhydrazone sodium salt of 4,5-benzotropone (**11**) and defined carbene–carbene rearrangements of **77**–**79** before finally it was verified by trapping of unstable intermediates **77**–**79** (Scheme 14) [77]. In 2002, McMahon reported obtaining the naphthylcarbene rearrangement manifold via the carbonyl groups of the isomeric benzotropones **11** and **12** (Scheme 14 and Scheme 33) [78]. Diazo compound **76** was prepared from 4,5-benzotropone hydrazone under oxidative conditions. Irradiation of matrix-isolated 7-diazo-7*H*-benzo[7]annulene (**76**) afforded a mixture of triplet 7*H*-benzo[7]annulenylidene (**77**), 2,3-benzobicyclo[4.1.0]hepta-2,4,6-triene (**78**), and triplet 2-naphthylcarbene (**79**). Formation of allene **83** as an alternative carbene rearrangement product was not detected under the studied photolysis conditions (Scheme 14).

**Scheme 15:**
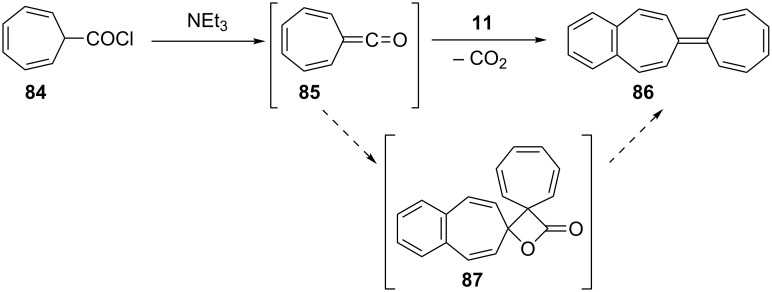
Synthesis of benzoheptafulvalene **86**.

Kitahara’s group reported the one-step synthesis of heptafulvalenes and benzoheptafulvalenes from monocyclic tropones and benzotropones [79]. The reaction of 4,5-benzotropone (**11**) and 8-oxoheptafulvene (**85**), prepared in situ via the reaction of cycloheptatriene-7-carboxylic acid chloride (**84**) with NEt_3_, afforded 3,4-benzoheptafulvalene **86** in 50% yield as fairly stable deep brown crystals (Scheme 15). The structure of **86** was confirmed by the spectroscopic data. The formation of the heptafulvalenes could be explained via an intermolecular [2 + 2] cycloaddition product such as **87** between the carbonyl group of tropones and the ketene C=C double bond of 8-oxoheptafulvene (**85**) followed by decarboxylation.

**Scheme 16:**
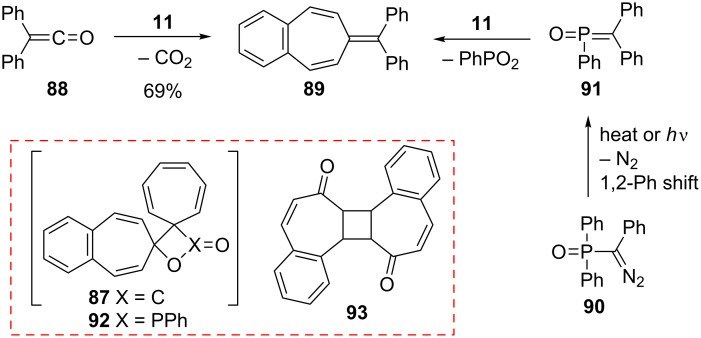
Synthesis of 7-(diphenylmethylene)-7*H*-benzo[7]annulene (**89**).

In a similar manner, the synthesis of 7-(diphenylmethylene)-7*H*-benzo[7]annulene (**89**) was reported in two different ways (Scheme 16). First, the addition of diphenylketene (**88**) to **11** resulted in the formation of benzoheptafulvalene **89** [80–81]. 7-(Diphenylmethylene)-7*H*-benzo[7]annulene (**89**) was also prepared via the oxaphosphetane **92** intermediate by treating 4,5-benzotropone (**11**) with (diphenylmethylene)(phenyl)phosphine oxide (**91**) generated thermally or photochemically from **90** [82]. Furthermore, dimeric byproduct **93** is also formed under photochemical conditions.

**Scheme 17:**
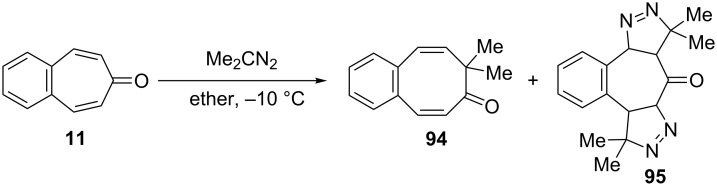
Reaction of 4,5-benzotropone (**11**) with dimethyl diazomethane.

**2.2.2. Ring expansion reactions via a tropone unit:** In 1975, Franck-Neumann and Martina reported the reaction of dimethyl diazomethane with tropone and benzotropones [83]. This reaction gave benzo-4,5-dimethyl-8,8-cyclooctatrienone (**94**, 30% isolable yield) as an insertion product via a carbonyl group and pyrazoline **95** as 1,3-dipolar addition product via double bonds (Scheme 17).

**Scheme 18:**
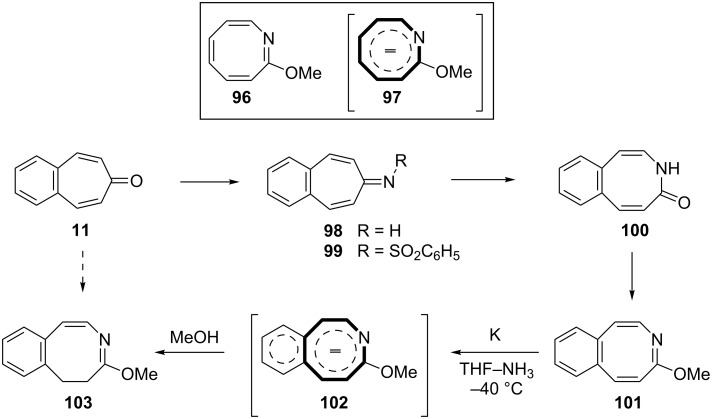
Synthesis of dihydrobenzomethoxyazocine **103**.

Azocine derivatives, eight-membered nitrogen heterocycles, exist as the core structure in many natural and non-natural products [84]. Nevertheless, 2-methyoxyazocine (**96**) reduces related aromatic 10π-electron dianion **97** (Scheme 18). Paquette’s group investigated benzo-annelation effects on azocine reactivity and the chemical and polarographic reduction of several methyoxy azocines [85–86]. 4,5-Benzotropone (**11**) was used as the logical starting material for the synthesis of benzomethoxyazocine **101** (Scheme 18). Treatment of **101** with potassium at –40 °C in NH_3_–THF (5:1), subsequent quenching by the addition of methanol, yielded dihydro derivative **103** (Scheme 18).

**Scheme 19:**
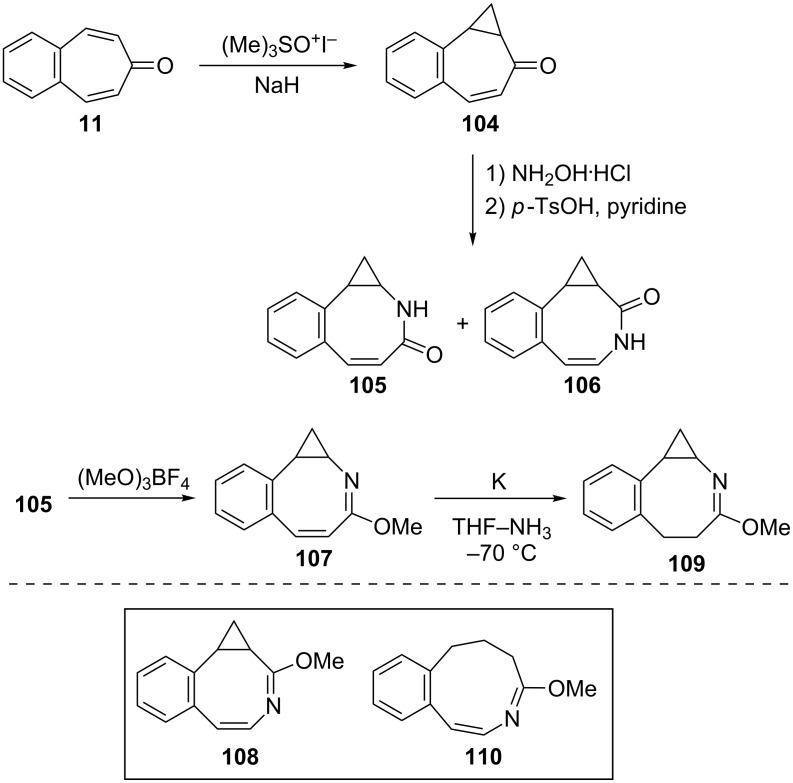
Synthesis and reducibility of benzo-homo-2-methoxyazocines.

In 1978, in order to examine heteroatomic influences on the possible generation of 9C–10π homoaromatic dianions, Paquette’s group described the synthesis and reducibility of benzo-fused-homo-2-methoxyazocines from benzotropones (Scheme 19) [87]. Firstly, dimethylsulfoxonium methylide addition to 4,5-benzotropone (**11**) provided the introduction of the cyclopropane ring required for two benzohomoazocines. Beckmann rearrangement of **104** resulted in a mixture of ring expansion products **105** and **106** in nearly equal proportions. This lactam mixture was then converted into the desired imidates and the imidates **107** and **108** were separated. Reduction of benzohomoazocine **107** proceeded without cleavage of its three-membered ring, whereas the internal cyclopropane σ bond of **108** underwent cleavage to form **110** (Scheme 19). Paquette’s group were unable to determine the formation of homoazocinyl dianion intermediates due to the added benzene ring in **107** and **108** and concluded that the presence of imino ether does not enhance the homoaromaticity of 9C–10π dianions.

**Scheme 20:**
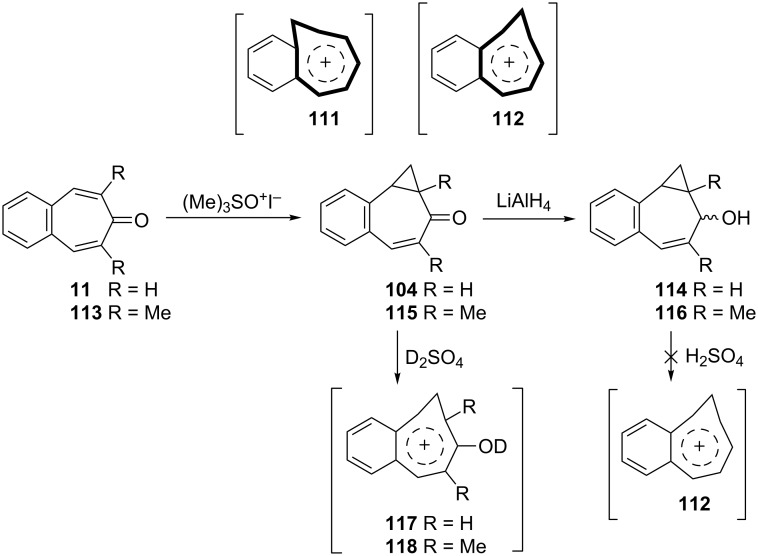
Synthesis of 4,5-benzohomotropones **104** and **115** from 4,5-benzotropones **11** and **113**.

Homoaromaticity, homotropylium cations, and homotropones have been extensively studied [88–123]. Merk’s group obtained with H_2_SO_4_ benzohomotropylium cation **111** starting from benzocyclooctatetraene and investigated its homoaromaticity (Scheme 20) [124]. Sugimura’s group attempted to prepare an alternative benzohomotropylium cation **112** and reported the synthesis of 4,5-benzohomotropones **104** and **115** from 4,5-benzotropones (Scheme 20). Although the conversions of 4,5-benzohomotropones to hydroxytropylium ions **117** and **118** in sulfuric acid were presumed, benzohomotropylium cation **112** was not detected from the reactions of the corresponding alcohol **114** in sulfuric acid [125].

**Scheme 21:**
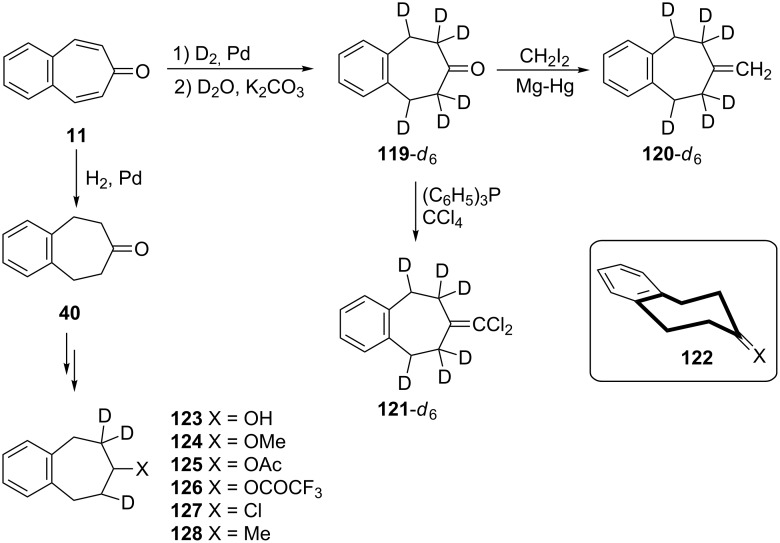
A catalytic deuterogenation of 4,5-benzotropone (**11**) and synthesis of 5-monosubstituted benzo[7]annulenes **123**–**128**.

**2.2.3. Reduction-based studies:** To define the stereochemical, conformational, and dynamic properties of both benzo[7]annulenones (and related compounds) and monosubstituted tetrahydro-7*H*-benzo[7]annulenone, St-Jacques' group benefited extensively from the use of dynamic nuclear magnetic resonance (DNMR) techniques [126–127]. A catalytic deuterogenation of 4,5-benzotropone (**11**) followed by deuteration led to deuterated 5,6,8,9-tetrahydro-7*H*-benzo[7]annulen-7-one **119-***d*_6_ with the presence of appreciable quantities of *d*_4_ and *d*_5_ species (Scheme 21). The compounds **120-***d*_6_ and **121-***d*_6_ were prepared from **119-***d*_6_ as shown in Scheme 21. NMR studies for these molecules show that the chair conformation **122** is predominant over the boat. Several 5-monosubstituted benzo[7]annulenes **123**–**128** were prepared using 4,5-benzotropone (**11**) as starting material and ^1^H and ^13^C NMR studies in each series of compounds revealed strikingly different substituent effects (Scheme 21).

**Scheme 22:**
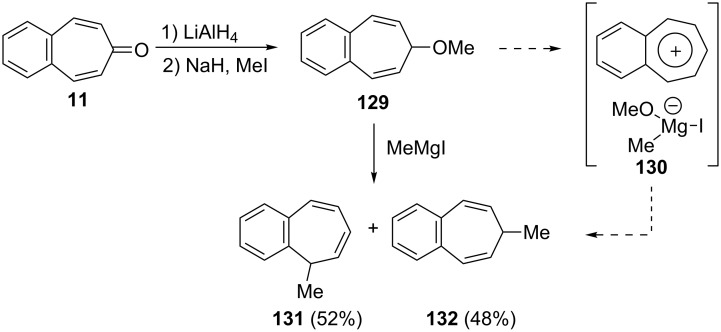
Synthesis of methyl benzo[7]annulenes **131** and **132**.

In order to perform reactions with alkyl Grignard reagents, Bertelli’s group realized the synthesis of 7-methoxy-7*H*-benzo[7]annulene (**129**) (Scheme 22) [128]. Reduction of 4,5-benzotropone (**11**) with LiAlH_4_ followed by etherification gave the corresponding ether **129**. Treatment of the ether **129** with MeMgI afforded an approximately equal mixture of two methyl benzo[7]annulenes, **131** and **132**. An intermediate, **130**, formed via the coordination of the Grignard reagent with ether was proposed to be operative in the reaction (Scheme 22).

**Scheme 23:**
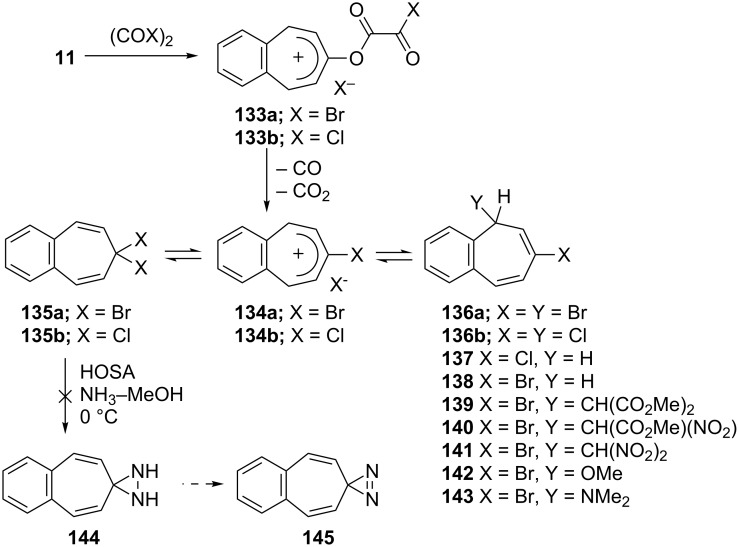
Ambident reactivity of halobenzo[7]annulenylium cations **133a/b**.

**2.2.4. Miscellaneous reactions: a. Halogenated benzo[7]annulenes and their synthetic potentials:** In 1978, Föhlisch’s group reported the synthesis and ambident reactivity of benzo[7]annulenylium cations **133a** and **133b** (Scheme 23) [49]. While the reaction of **11** with oxalyl bromide yielded bromobenzo[7]annulenenylium bromide **134a** as a stable carbenium salt, the reaction of oxalyl chloride or phosgene with **11** afforded 7,7-dichloro-7*H*-benzo[7]annulene (**135b**) as a covalent compound that ionizes in liquid SO_2_ to the cation **134b**. Treatment of cations with nucleophiles that are preferably added to the benzylic position (C-5 or C-9) yielded chloro- and bromo-5*H*-benzo[7]annulenes **136**–**143**. According to Hückel molecular orbital (HMO) calculations, this observed regiochemistry is attributed to the highest positive charge density at the benzylic position, which is the favored process under kinetic conditions. During the attempted preparation of 4,5-benzotropone diaziridine **144**, the synthesis of 7,7-dichloro-7*H*-benzo[7]annulene (**135b**) was also carried out from the reaction of 4,5-benzotropone (**11**) with SOCl_2_ in a quantitative yield (Scheme 23) [77–78]. However, all attempts to synthesize **144** from **135** have failed.

**Scheme 24:**
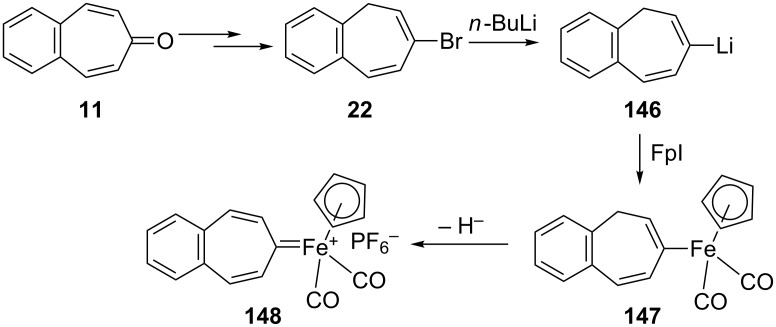
Preparation of benzo[7]annulenylidene–iron complexes **147**.

7-Bromo-5*H*-benzo[7]annulene (**22**) was also used as a key molecule in the synthesis of benzo[7]annulenylidene–iron complexes **147** and **148** (Scheme 24) [129]. The monobromide **22** obtained from the reaction of oxalyl bromide with **11** followed by *n*-BuLi-reduction converted into **146** and then into yellow-brown complex **147** by treatment with a cold solution of (η^5^-C_5_H_5_)(CO)_2_FeI (FpI). After chromatography of **147** over alumina with a pentane–benzene mixture, the complex **147** oxidized to **148** as a fairly air-stable red-brown solid.

**Scheme 25:**
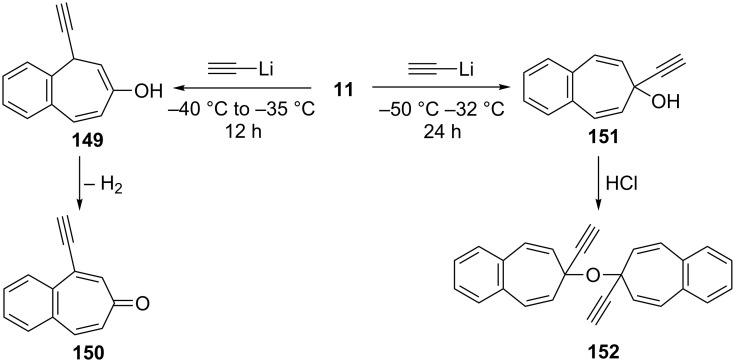
Synthesis of 1-ethynylbenzotropone (**150**) and the etheric compound **152** from 4,5-benzotropone (**11**) with lithium acetylide.

**b. Nucleophilic addition to 4,5-benzotropone (11):** Ried’s group realized the reaction of 4,5-benzotropone (**11**) and its derivatives with lithium acetylide as a nucleophile between −50 and −32 °C [130]. While the possible 1,4-conjugate addition product **149** was oxidized to 1-ethynylbenzotropone **150** in situ, the etheric compound **152** was obtained from the reaction of 1,2-addition product **151** with HCl (Scheme 25).

**Scheme 26:**
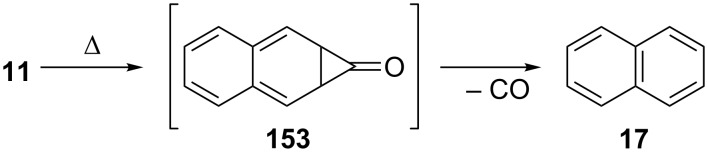
Thermal decomposition of 4,5-benzotropone (**11**).

**c. Decarbonylation of 4,5-benzotropone (11):** The mechanism for the neutral and radical-cationic decarbonylation of tropone and benzannulated tropones was compared by both experimental techniques and by means of MNDO calculations (Scheme 26) [131]. While the key steps for the thermal decomposition of tropones are electrocyclic ring closure and cheletropic CO extrusion to give an aromatic system, the cationic reactions occur with ring closure followed by the opening to a benzoyl-type ion, which is the actual precursor of the CO loss (Scheme 26).

**Scheme 27:**
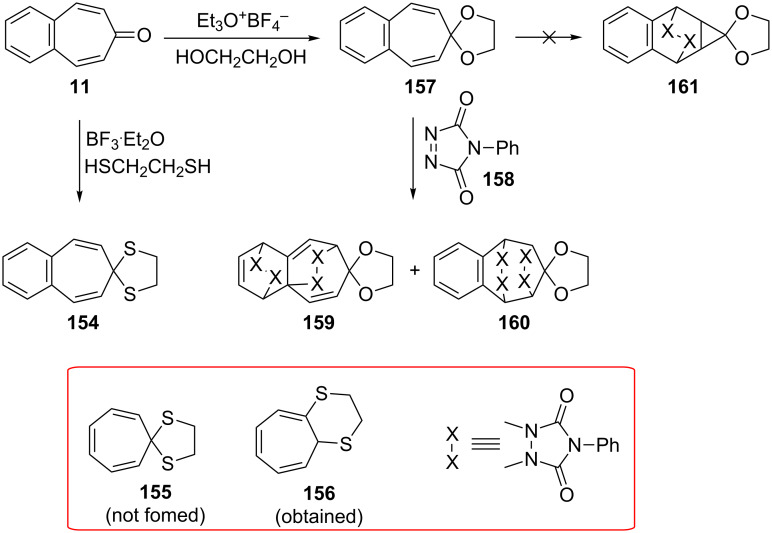
Reaction of 4,5-benzotropone (**11**) with 1,2-ethanediol and 1,2-ethanedithiol.

**d. Ketalization of 4,5-benzotropone (11):** Cavazza’s group reported their unsuccessful attempts to obtain tropone dithioketals such as **155** [132]. The treatment of 4,5-benzotropone (**11**) with 1,2-ethanedithiol and BF_3_**^.^**Et_2_O in MeOH gave the expected dithioketal **154**, whereas the reaction of tropone under similar conditions presented complications from rapid [1,7] sigmatropic shifts of unhindered alkylthio groups to give bicyclic 1,7-disubstituted cycloheptatrienes like **156** (Scheme 27). Leitich’s group reported the synthesis and cycloaddition of tropone ethylene acetal and benzotropone ethylene acetal **157** (Scheme 27) [133]. The ketal **157** was prepared from the reaction of 4,5-benzotropone (**11**) with ethylene glycol in the presence of triethyloxonium tetrafluoroborate. The cycloaddition of **157** with 4-phenyl-1,2,4-triazoline-3,5-dione (**158**) gave the cycloadducts **159** and **160** via the cycloheptatriene form. However, usually the norcaradiene type product **161** observed with cycloheptatrienes was not formed (Scheme 27).

### 3. Chemistry of 2,3-benzotropone (**12**)

#### 3.1. Synthesis of 2,3-benzotropone (**12**)

**Scheme 28:**
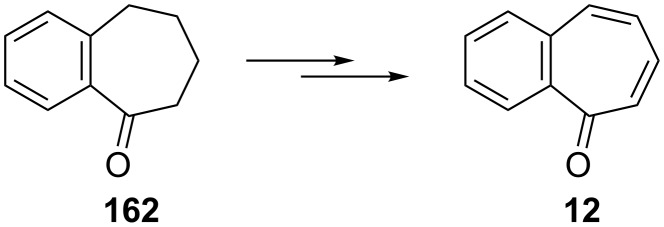
Conversions of 1-benzosuberone (**162**) to 2,3-benzotropone (**12**).

Several procedures relating to the synthesis of 2,3-benzotropone (**12**) were reported. The vast majority of these procedures utilize commercially available 1-benzosuberone (6,7,8,9-tetrahydro-5*H*-benzo[7]annulen-5-one, **162**) (Scheme 28).

**Scheme 29:**
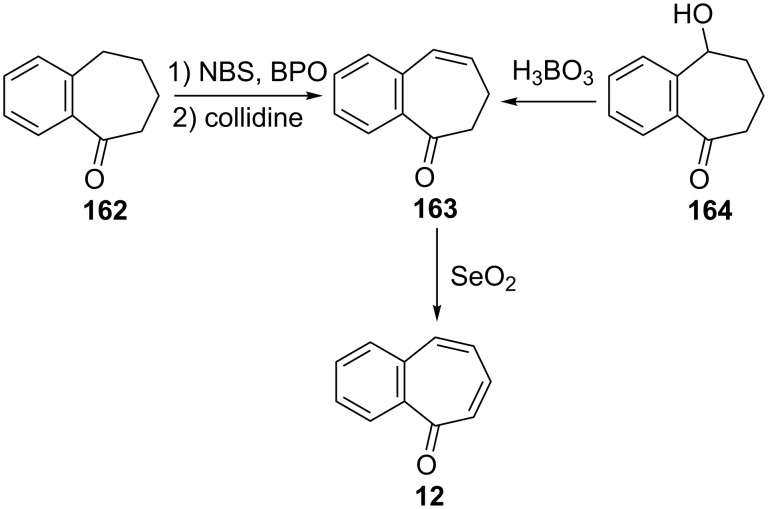
Synthesis strategies for 2,3-bezotropone (**12**) using 1-benzosuberones.

**3.1.1. Synthesis using 1-benzosuberone (162):** In 1959, Buchanan’s group realized a nontedious method for the synthesis of 2,3-benzotropone (**12**) starting with 1-benzosuberone (**162**) (Scheme 29) [134]. First, the unsaturated ketone **163**, which is called Julia’s ketone, was prepared by NBS-bromination in the presence of a trace of benzoyl peroxide (BPO) and followed by dehydrobromination. Another synthesis of Julia’s ketone was achieved by dehydration of the known keto-alcohol **164** by boric acid. Oxidation of Julia’s ketone with selenium dioxide gave 2,3-benzotropone (**12**). An alternative synthesis for **12**, which represents a feasible route to avoid the disadvantage of selenium dioxide, is also bromination of Julia’s ketone **163** followed by spontaneous elimination of hydrogen bromide at the temperature of the reaction.

**Scheme 30:**
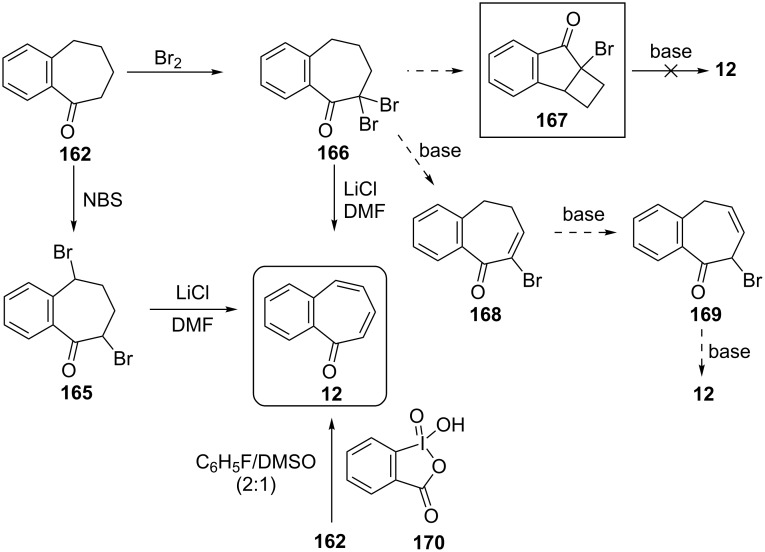
Oxidation-based synthesis of 2,3-benzotropone (**12**) via 1-benzosuberone (**162**).

2,3-Benzotropone (**12**) was also prepared by bromination of 1-benzosuberone (**162**) using both NBS and molecular bromine followed by dehydrobromination (using lithium chloride in dimethylformamide) of the resulting dibromo derivatives (Scheme 30) [135–136]. Moreover, the formation mechanism of the elimination of HBr from the germinal dibromide was investigated by Jones’ group [135]. Jones’ group also investigated the mechanism of the elimination of HBr from geminal-dibromide **166** with the preparation of possible intermediate bromo-tetrahydrocyclobuta[*a*]inden-7-one **167** [136]. As the reaction of **167** does not work under basic conditions, it is supposed that the reaction takes place via an acid-catalyzed double bond isomerization followed by an elimination reaction. Moreover, Ghosh’s group repeated the synthesis of **12** through a molecular bromination–dehydrobromination sequence starting with **162** [137].

Hypervalent iodine(V)-based reagents such as IBX (or 2-iodoxybenzoic acid) and Dess–Martin periodinane (DMP) are commonly used in organic synthesis as oxidizing agent to form both unsaturated carbonyl compounds and conjugated aromatic carbonyl systems. Nicolaou’s group reported a general method for the mild, swift, and highly efficient oxidation of alcohols, ketones, and aldehydes to unsaturated compounds in one pot (Scheme 30) [138–139]. Accordingly, an IBX-controlled dehydrogenation through single-electron-transfer-based oxidation processes of **162** gave **12** in 60% yield.

**Scheme 31:**
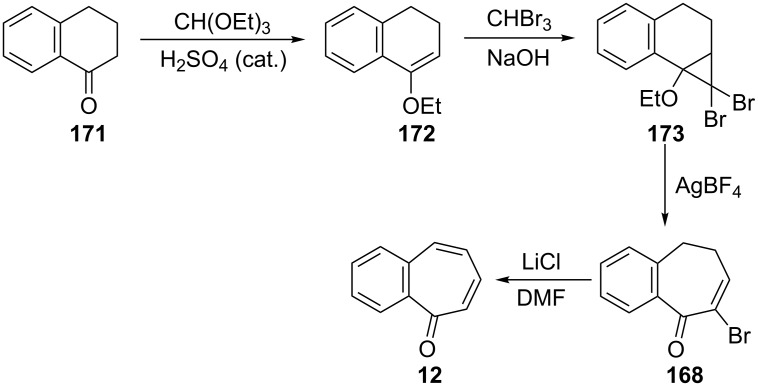
Synthesis of 2,3-benzotropone (**12**) from α-tetralone (**171**) via ring-expansion.

**3.1.2. Other synthetic approaches:** A convenient synthesis of 2,3-benzotropone (**12**) from α-tetralone (**171**) by ring expansion was performed by Sato’s group (Scheme 31) [140]. First, 1-ethoxy-3,4-dihydronaphthalene (**172**) was prepared by reacting α-tetralone (**171**) with ethyl orthoformate in the presence of an acid catalyst. Subsequent successive reactions are dihalocarbene addition to enolether **172**, ring expansion of the adduct **173** to halocycloheptadienone **168**, and dehydrohalogenation of **168** with lithium chloride. Later, McMahon's group reiterated the same protocol in their work on achieving the rearrangement of naphthylcarbene [78].

**Scheme 32:**
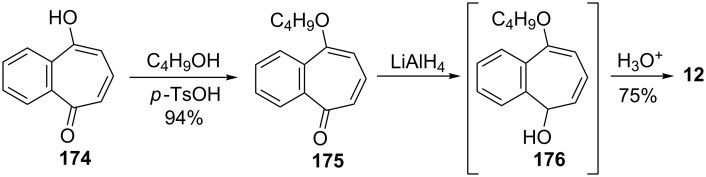
Preparation of 2,3-benzotropone (**12**) by using of benzotropolone **174**.

Rennhard’s group reported the formation of 2,3-benzotropone (**12**) from benzotropolone **176** (Scheme 32) [141–142]. After the etherification of benzotropolone **174** using isobutanol in the presence of *p*-toluenesulfonic acid (*p*-TsOH), the reduction of **175** with lithium aluminum hydride afforded the intermediate **176**, which was converted to 2,3-benzotropone (**12**) under acidic conditions.

#### 3.2. Reactions of 2,3-benzotropone (**12**)

**Figure 6:**
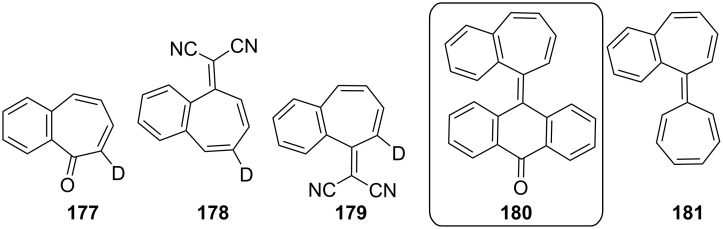
Benzoheptafulvenes as condensation products of 2,3-benzotropone (**12**).

**3.2.1. Reactions via a carbonyl group:** Among the most common reactions for 2,3-benzotropone (**12**) are condensation reactions with active methylenic compounds, resulting in the formation of benzoheptafulvenoids (Figure 6). For example, Machiguchi reported that the condensation reaction of deuterium tracer 2,3-benzotropone **177** with malononitrile to yield 8,8-dicyano-2,3-benzoheptafulvene **178** via reaction paths including the choice of a Michael-type attack of the nucleophile at the C-4 position to Knoevenagel-type attack at the C-1 position [143]. Benzannulated quinotropylidene **180** was produced by the condensation reaction of 2,3-benzotropone (**12**) and 10*H*-anthracen-9-one [63]. 2,3-Benzotropone (**12**) was transformed into the corresponding benzoheptafulvalene **181** using the ketene addition protocol illustrated in Scheme 15 and Figure 6.

**Scheme 33:**
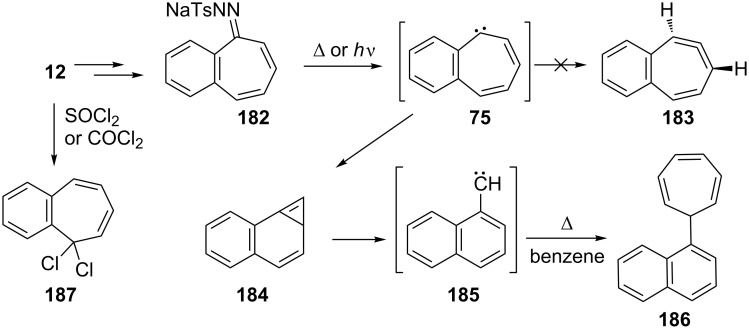
Conversion of 2,3-benzotropone (**12**) to tosylhydrazone salt **182** and *gem*-dichloride **187**.

The thermal decomposition of the obtained tosylhydrazone salt **182** from 2,3-benzotropone (**12**) afforded a trapping product of 1-naphthylcarbene (**185**) [128], while the allenic rearrangement product **183** for the carbene **75** was not detected in the photolysis of a diazo compound (Scheme 33) [78]. 2,3-Benzotropone (**12**) was converted to gem-dichloride **187** to achieve diazirine as carbene precursor (Scheme 33) [77,144].

**Figure 7:**
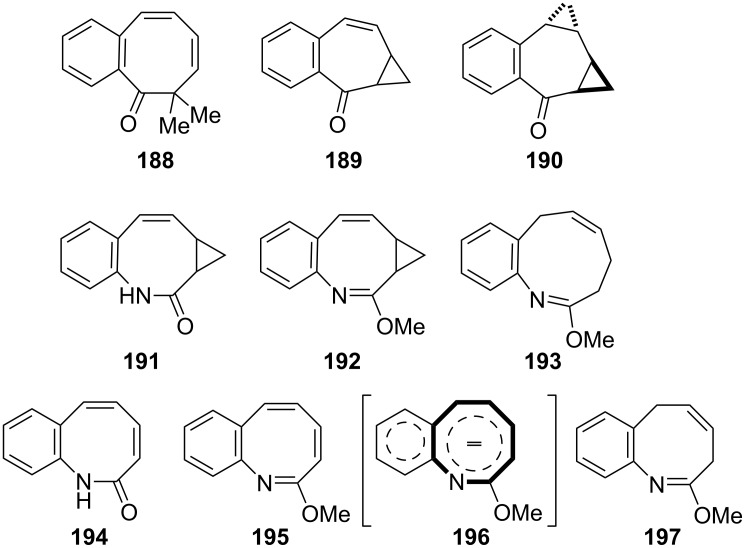
Benzohomoazocines **191**–**193** and benzoazocines **194**–**197**.

**3.2.2. Ring-expansion reactions via a tropone unit:** The ring-expansion product **188**, which is a net insertion of a diazomethane unit into the tropone, was prepared from the reaction of 2,3-benzotropone (**12**) with dimethyl diazomethane (Figure 7) [83]. Benzohomoazocines **191**–**193** and benzomethoxyazocines **195**–**197** were prepared using a similar protocol illustrated in Scheme 18 and Scheme 19 (Figure 7) [84–87,145]. The cyclopropane ring in **192** was reduced to the corresponding dihydroazonine **193**.

**Scheme 34:**
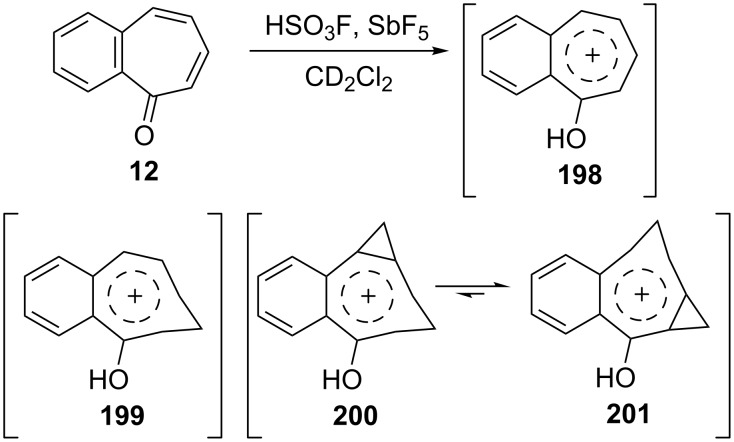
From 2,3-benzotropone (**12**) to carbonium ions **198**–**201**.

Additionally, Ogliaruso’s group prepared 2,3-benzo-6,7-monohomotropone (**189**) and *trans*-2,3-benzo-4,5:6,7-bishomotropone (**190**) from the nonselective reaction of 2,3-benzotropone (**12**) with dimethyloxosulfonium methylide in yields of 35% and 28% and investigated the structural characterization of these compounds by extensive NMR analysis (Figure 7 and Scheme 34) [146]. In addition, carbonium ions **198**–**201**, prepared via the protonation of 2,3-benzotropone (**12**), homotropone **189,** and bishomotropone **190**, and their deuterated analogues using fluorosulfuric acid–antimony pentafluoride were investigated using NMR spectroscopy (Scheme 34) [147]. NMR investigation of the carbonium ion formed from **189** indicated the formation of the carbonium ion **199** with complete electron delocalization, whereas the carbonium ion **200** (and **201**) formed from **190** indicated considerably less electron delocalization (Scheme 34).

**Scheme 35:**
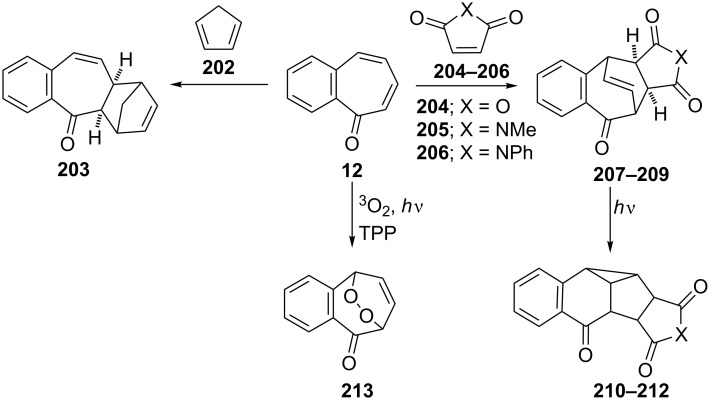
Cycloaddition reactions of 2,3-benzotropone (**12**).

**3.2.3. Cycloaddition reactions:** 2,3-Benzotropone (**12**) possesses a high functional tolerance towards both the diene and dienophile and undergoes the Diels–Alder reaction. Ghosh’s group reported an exclusive peri-, regio-, and stereoselective cycloaddition reaction of 5*H*-benzo[7]annulen-5-one (Scheme 35) [137]. The intermolecular [4 + 2] cycloaddition of 2,3-benzotropone (**12**) with cyclopentadiene (**202**) in the presence of boron trifluoride occurred totally periselectively, regioselectively, and *exo*-diaselectively to afford the adduct **203** in 75% yield. The Diels–Alder reaction of 2,3-benzotropone (**12**) with various dienophiles has also been reported. In the first example, Rennhard’s group realized the cycloaddition of benzotropone **12** with maleic anhydride (**204**) to give a tricyclic adduct **207** (in 90% yield) (Scheme 35) [141–142]. Later, Middlemiss’ group also used dienophiles such as maleic anhydride (**204**), *N*-methylmaleimide (**205**), and *N*-phenylmaleimide (**206**) to give *endo*-adducts **207**–**209** [148]. Furthermore, these ethenobenzocycloheptenones **207**–**209** underwent di-π-methane photo-rearrangement to **210**–**212** exclusively (Scheme 35). Moreover, Dastan’s group prepared bicyclic endoperoxide **213** in 74% yield from 2,3-benzotropone (**12**) via tetraphenylporphyrine (TPP)-sensitized photo-oxygenation (Scheme 35) [149].

**Scheme 36:**
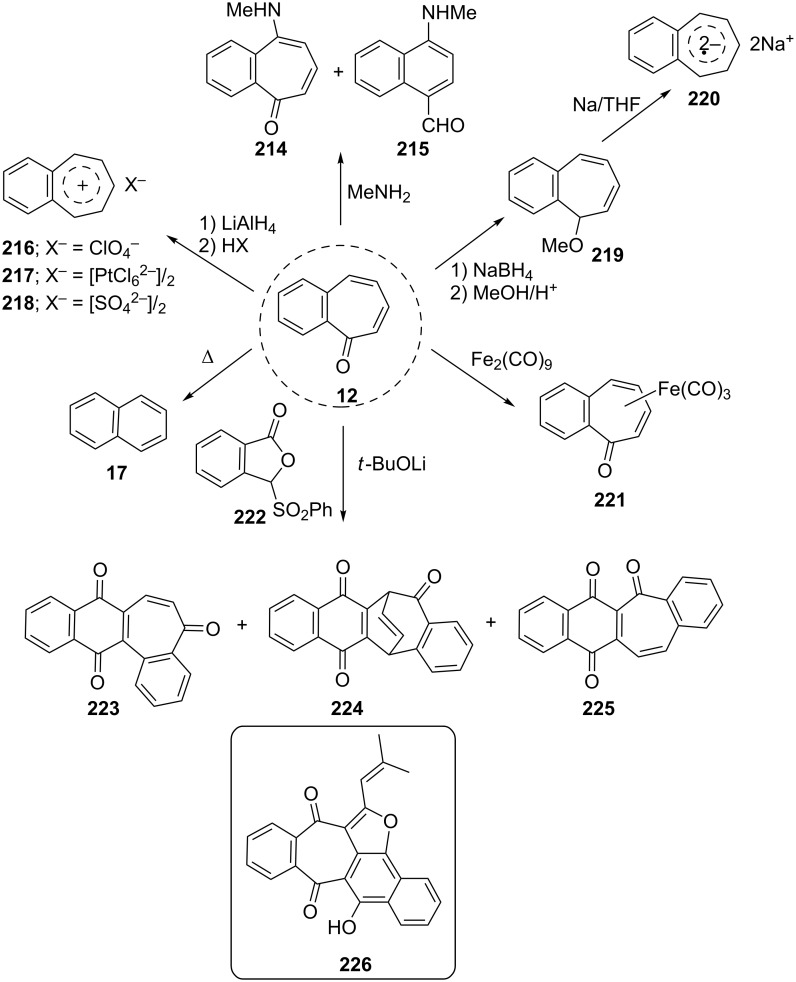
Reaction of 2,3-benzotropone (**12**) with various reagents and compounds.

**3.2.4. Miscellaneous transformations:** Machiguchi reported that the oxidative amination of 2,3-benzotropone (**12**) with methylcopperamine sulfate in aqueous methylamine at room temperature afforded 4-methylamino-1-naphthalene carbaldehyde (**215**) with a trace amount of 4-methylamino-2,3-benzotropone (**214**) via reaction paths including the dishing of Michael-type attack of the nucleophile at the C-4 position to a normal attack at the C-1 position. (Scheme 36) [143]. Preparation of benzotropylium cations **216**–**218** was reported by Rennhard’s group [142]. The corresponding cations **216**–**218** were produced by reduction of **12** with LiAlH_4_ followed by treatment with Brønsted acids (Scheme 36) [142]. Bauld and Brown reported the ready generation of the first detectable dianion radicals as outlined in Scheme 36 [150]. Benzo[7]annulenide (benzotropenide) dianion radical **220** was generated in two steps from **12** via benzotropyl methyl ether **219**. Holzmann’s group described the thermal and electron impact-induced decarbonylation reaction of **12** (Scheme 36) [131]. Tajiri’s group reported the resolution and determination of the kinetic parameters of the optically active 2,3-benzotropone(tricarbonyl)iron complex **221** using high-performance liquid chromatography (HPLC) and circular dichroism (CD) measurements, respectively (Scheme 36) [151]. In conjunction with the efficient preparation of 5*H*-benzo[7]annulen[1,2-*b*]naphthalenes as key intermediates to the naturally occurring red pigment radermachol (**226**), condensation of the anion of the isobenzofuranone **222** with 2,3-benzotropone (**12**) was also performed (Scheme 36). This condensation followed by incipient oxidation gave the quinone **223** (15%), the bridged bicyclic product **224** (7%), and **225** (28%) [152].

### 4. Chemistry of 3,4-benzotropone (**13**)

#### 4.1. Generation, characterization, and reaction

**Figure 8:**
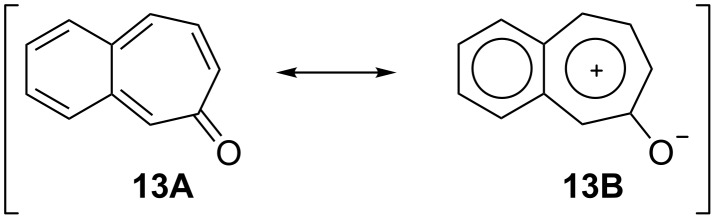
3,4-Benzotropone (**13**) and its resonance structure.

3,4-Benzotropone (**13**) is of considerable interest for both theoretical and preparative reasons. The aromaticity of 3,4-benzotropone (**13A**) is considered to depend on the contribution of electronically polarized form **13B** such as tropone **1A** (Figure 8) [153–154]. Kurihara’s group reported the calculated resonance energies and bond currents for benzotropones and troponoid compounds [155]. Although benzotropones **11** and **12** have been isolated, 3,4-benzotropone (**13**) is rather unstable and has not been isolated. This instability is attributed to the *o*-quinoidal structure of **13** because it has no sextet electron system in the benzene ring and there are limited reports on the *o*-quinoidal 3,4-benzotropones **13**.

**Scheme 37:**
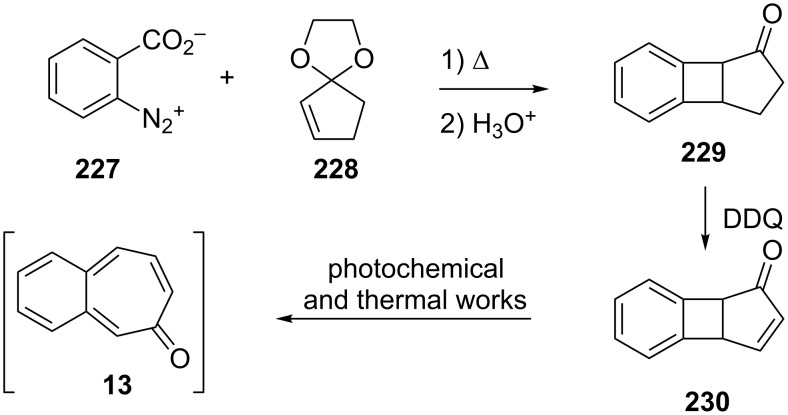
Synthesis of 6,7-benzobicyclo[3.2.0]hepta-3,6-dien-2-one (**230**).

**Figure 9:**
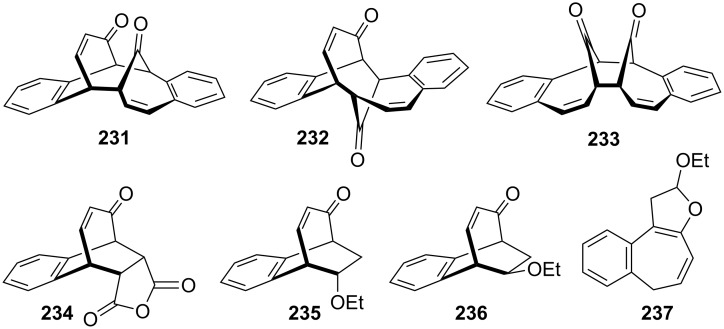
Photolysis and thermolysis products of **230**.

Considering the known reactivity of benzocyclobutenes, i.e., their isomerisation to *o*-quinodimethanes, Tsuji’s group used 6,7-benzobicyclo[3.2.0]hepta-3,6-dien-2-one (**230**) as a precursor to produce **13**. They reported the first generation and spectroscopic characterization of this elusive molecule obtained by electrocyclic ring-opening reaction of **230** through irradiation in a rigid medium at low temperature or by thermolysis at high temperature [153]. As shown in Scheme 37, compound **230** was synthesized through the addition of benzyne to 2-cyclopentenone acetal (**228**) followed by hydrolysis and subsequent oxidation of the resultant ketone **229** with DDQ. Irradiation (>300 nm) of **230** in EPA (a 5:5:2 mixture of ether, isopentane, and ethanol) at −196 °C led to the formation of **13**, which exhibited the development of characteristic UV–vis absorption in the range 300–550 nm. In addition to product **13**, two [π8 + π10] dimers **231** and **232** at –78 °C were also isolated (Figure 9) [153]. In a subsequent study, Tsuji’s group described details of the spectral and chemical properties of **13** [154]. The IR spectroscopic results showed a substantial contribution of **13B** to **13A** in the ground state. Moreover, it was found that the photochemical behavior of **230** depended on the state of the irradiation medium. For example, the smooth [π10 + π10] dimerization of **13** to give dimeric product **233** was realized with the irradiation (>420 nm) of **13** in a fluid EPA solution below −100 °C [154]. Furthermore, the IR spectra of 3,4-benzotropone (**13**) generated in matrices at 13 K by the photoisomerization of **230** were directly observed [156]. In addition, the thermal generation of **13** from **230** was investigated [153–154]. When **230** with 10 equiv of maleic anhydride in benzene at 220 °C was reacted, [π2 + π8] cyclo-adduct **234** as a single product was isolated in 52% yield (Figure 9) [153–154]. The thermolysis of **230** in the presence of ethyl vinyl ether gave three volatile products **235**–**237** in GLC yields of 10%, 7%, and 15%, respectively (Figure 9) [154].

### 5. Benzotropolones

**Figure 10:**
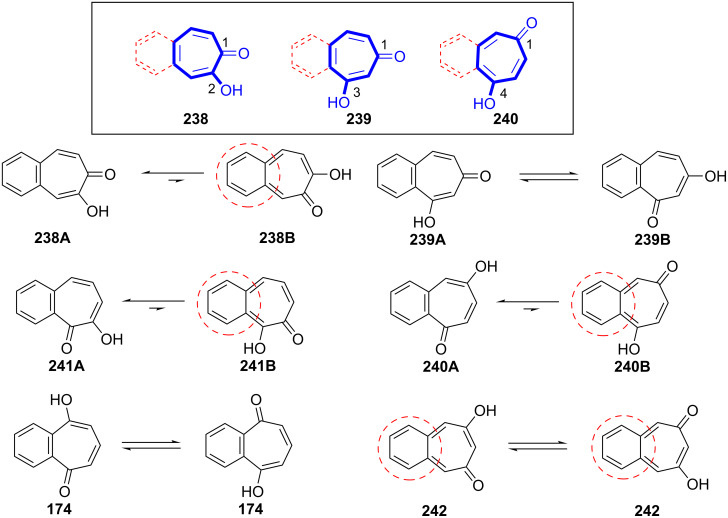
Benzotropolones and their tautomeric structures.

Benzannulation to the tropolone scaffold can give numerous tautomeric hydroxybenzotropones or benzotropolenes. Figure 10 shows **238A** (or **241A**–**240**) as single tautomers, whereas **239** and **174** are depicted as a mixture of tautomers. Moreover, benzenoid structures as **238A** are more stable than *o*-quinoidal structures as **238B** due to Clar’s π-sextet rule.

#### 5.1. 2-Hydroxy-4,5-benzotropone (**238**)

**Scheme 38:**
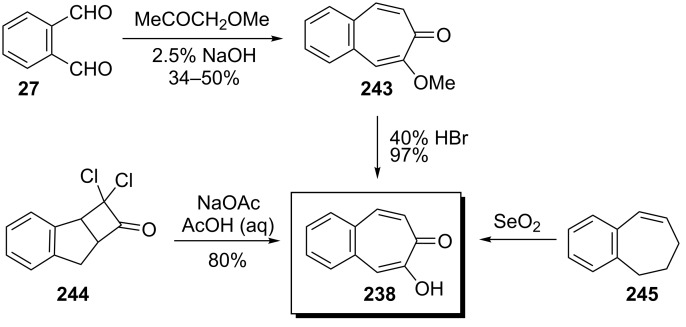
Synthesis strategies of 4,5-benzotropolone (**238**).

**5.1.1. Synthesis of 2-hydroxy-4,5-benzotropone (238):** Tarbell’s group reported the first synthesis of 2-hydroxy-4,5-benzotropone (**238**) starting from phthalaldehyde (**27**) in two steps (Scheme 38) [157]. Condensation of **27** with methoxyacetone in the presence of NaOH and then cleavage of the methyl ether **243** by strong acid afforded 2-hydroxy-4,5-benzotropone (**238**). Turner’s group reported a new method for the synthesis of 4,5-benzotropolone (**238**) via the cycloaddition of dichloroketene (generated *in situ* from trichloroacetyl chloride) with indene followed by hydrolysis of the adduct **244** with sodium acetate in aqueous acetic acid (Scheme 38) [158]. In addition, Stevens’ group reported a method for the synthesis of **238** using a strategy similar to that of Turner’s group (Scheme 38) [159]. One-step synthesis for **238** was described by Christol’s group through the oxidation of benzocyclopheptene **245** with SeO_2_ in 35% yield (Scheme 38) [160].

**Scheme 39:**
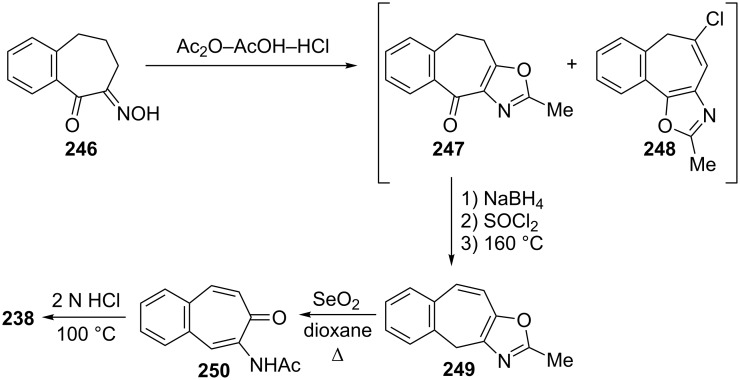
Synthesis protocol for 2-hydroxy-4,5-benzotropone (**238**) using oxazole-benzo[7]annulene **247**.

Galantay’s group described a novel synthetic protocol for benzotropolones using the oxazole-benzo[7]annulenes **247** and **248**, which may be easily obtained from the reaction of α-oximino-benzosuberone **246** with Ac_2_O/AcOH/HCl (Scheme 39) [161]. The olefin **249**, derived from reduction, chlorination, and elimination of **247**, was converted by SeO_2_ in refluxing dioxane or xylene to the acetamido-benzotropone **250**, which in turn can be hydrolyzed to 2-hydroxy-4,5-benzotropone (**238**).

**Figure 11:**
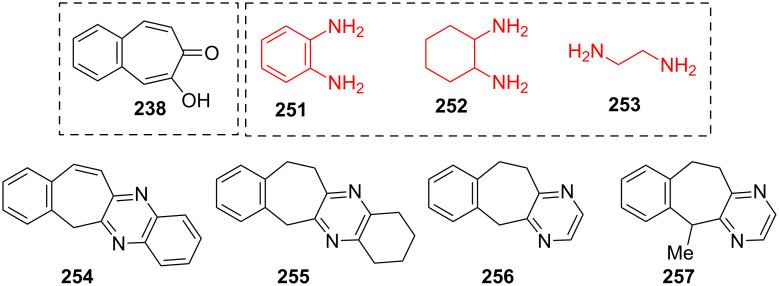
Some quinoxaline and pyrazine derivatives **254**–**256** prepared from 4,5-benzotropolone (**238**).

**5.1.2. Reactions of 2-hydroxy-4,5-benzotropone (238):** Some quinoxaline and pyrazine derivatives **254**–**256** were synthesized from 1,2-phenylenediamine (**251**), 1,2-diaminocyclohexane (**252**), and ethylenediamine (**253**) with 4,5-benzotropolone **238** (Figure 11) [162]. Compound **256** can be converted to methylated **257**.

**Scheme 40:**
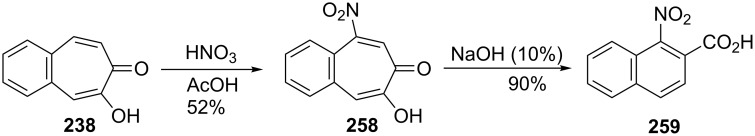
Nitration product of 4,5-benzotropolone (**238**) and its isomerization to 1-nitro-naphthoic acid (**259**).

Tarbell’s group also reported 4,5-benzotropolone **238** and its methyl ether do not give the characteristic aromatization reactions as colchicine and monocyclic tropolones, and explained that **238** is a weaker acid than colchicine or tropolone [157]. However, the conversion of **238** to 1-nitro-2-naphthoic acid (**259**) was reported in two steps including the treatment of **238** with concentrated nitric acid in glacial acetic acid, followed by the reaction of 10% aqueous alkali at room temperature (Scheme 40) [157].

#### 5.2. 6-Hydroxy-2,3-benzotropone (**239**)

**Scheme 41:**
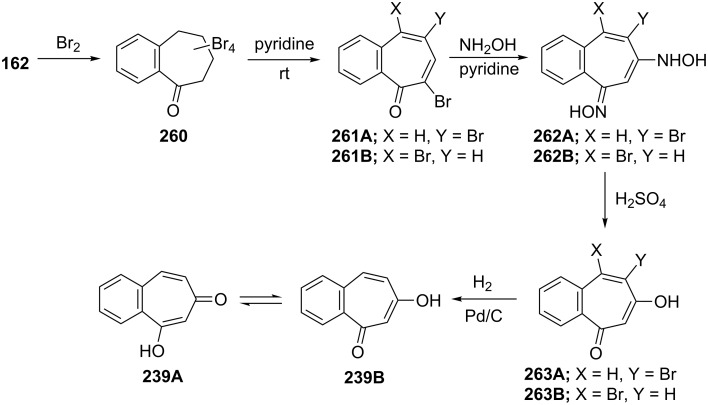
Synthesis protocol for 6-hydroxy-2,3-benzotropone (**239**) from benzosuberone (**162**).

**5.2.1. Synthesis of 6-hydroxy-2,3-benzotropone (239):** Takahashi and co-workers described a route for 6-hydroxy-2,3-benzotropone (**239**) starting from benzosuberone (**162**) [163]. Firstly, a mixture of isomeric dibromides **261** was prepared with excessive bromination of **162** followed by subsequent dehydrobromination. By treatment of dibromides **261** with hydroxylamine in pyridine, 5- and 4-bromo-6-hydroxylamino-2,3-benzotropone oximes **262** were obtained. Hydrolysis of these oximes **262** with sulfuric acid gave 5-bromo-6-hydroxy-2,3-benzotropone and the 4-bromo isomer **263**, which were debrominated with catalytic hydrogenation to give **239** (Scheme 41). Although **239** is capable of existing as two tautomeric mixture tautomers, **239A** and **239B**, which predominates is not clear, and the formation of a single methyl ether, acetate, and 2,4-dinitrophenylhydrazone has been reported [163].

**Scheme 42:**
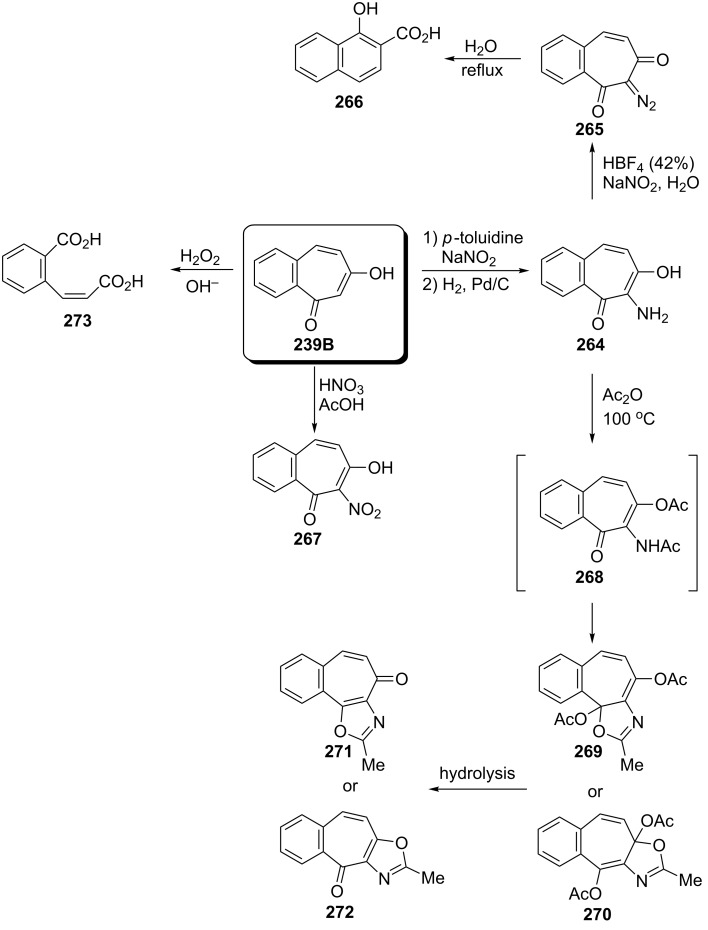
Various reactions via 6-hydroxy-2,3-benzotropone (**239**).

**5.2.2. Reactions of 6-hydroxy-2,3-benzotropone (239):** Hoshino’s group reported the synthesis and chemical transformations of azo, nitro, and amino derivatives of 6-hydroxy-2,3-benzotropone (**239**) (Scheme 42) [164]. While 7-amino derivative **264** was prepared via diazo coupling of **239** with diazotized *p*-toluidine in a pyridine solution followed by hydrogenation, the nitration of **239** in acetic acid solution afforded nitro compound **267**. Nitro compound **267** was also hydrogenated to produce 7-amino derivative **264**. Diazoketone **265** was prepared from **264** with sodium nitrite in fluoroboric acid (HBF_4_) and its Wolff rearrangement under reflux conditions in water gave 1-hydroxy-2-naphthoic acid (**266**). Acetylation of **264** with sodium acetate in acetic anhydride at 100 °C for 30 min afforded an unusual product, **269** or **270** via intermediate **268**. The hydrolysis of **269** (or **270**) provided a compound that is assumed to be an oxazolobenzotropone based on its infrared absorption spectrum. In fact, the authors reported that the correct structures for both pairs (**271** or **272**) are not clear. However, the oxidation of both **239** and **264** to *o*-carboxycinnamic acid (**273**) was also reported under alkaline hydrogen peroxide conditions (Scheme 42) [163–164]. Furthermore, bromination of 6-hydroxy-2,3-benzotropone (**239**) and corresponding transformations will be covered in the next sections.

**Scheme 43:**
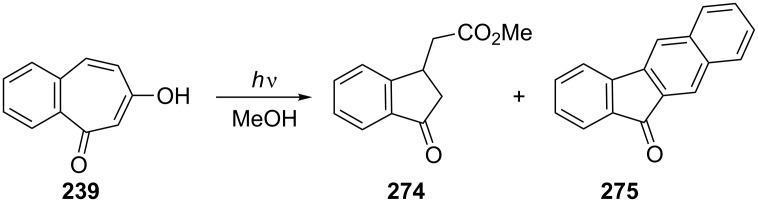
Photoreaction of 6-hydroxy-2,3-benzotropone (**239**).

Photoreaction of 6-hydroxy-2,3-benzotropone (**239**) was reported by Yoshioka and Hoshino (Scheme 43) [165]. The irradiation of **239** in methanol with Pyrex-filtered light gave the products **274** and **275** in 25% and 2% yields, respectively, accompanied by undefined materials.

#### 5.3. 7-Hydroxy-2,3-benzotropone (**241**)

**Scheme 44:**
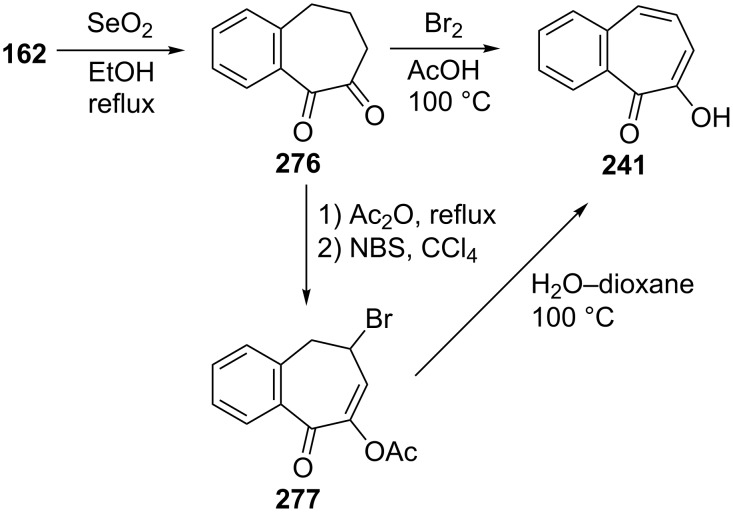
Synthesis of 7-hydroxy-2,3-benzotropone (**241**) from benzosuberone (**162**).

**Scheme 45:**
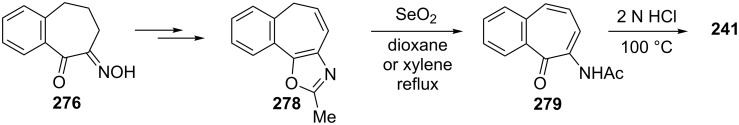
Synthesis strategy for 7-hydroxy-2,3-benzotropone (**241**) from ketone **276**.

**5.3.1. Synthesis of 7-hydroxy-2,3-benzotropone (241):** The first synthesis of 7-hydroxy-2,3-benzotropone (**241**) was described by Cook’s group (Scheme 44) [166–167]. 7-Hydroxy-2,3-benzotropone (**241**) was prepared from benzosuberone (**162**) by oxidation with selenium dioxide in boiling ethanol, followed by dehydrogenation with bromine in acetic acid at 100 °C. Another method for the synthesis of 7-hydroxy-2,3-benzotropone (**241**) starting from the reaction of diketone **276** with boiling acetic anhydride was achieved by Maignan (Scheme 44) [168]. The reaction of enol-acetate with NBS led to **277**, which was heated in water–dioxane at 100 °C to give 7-hydroxy-2,3-benzotropone (**241**) by an elimination process. Galantay’s group also reported the synthesis of 7-hydroxy-2,3-benzotropone (**241**), which was similar to the synthesis of 2-hydroxy-4,5-benzotropone (**238**) as depicted in Scheme 38 (Scheme 45) [161].

**Scheme 46:**
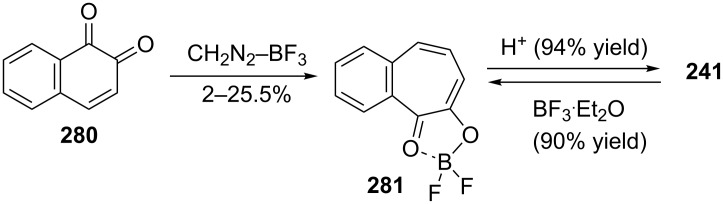
Synthesis of 7-hydroxy-2,3-benzotropone (**241**) from β-naphthoquinone (**280**).

Sato’s group reported the synthesis of 7-hydroxy-2,3-benzotropone (**241**) via the ring expansion pathway of β-naphthoquinone (**280**) with diazomethane under various conditions and hydrolysis steps (Scheme 46) [169]. The boron trifluoride etherate-promoted ring expansion reactions were carried out at various quinone/BF_3_**^.^**OEt_2_/CH_2_N_2_ molar ratios in a different solvent under an atmosphere of nitrogen and with cooling in an ice bath and 3,4-benzotropolonoboron difluoride **281** was obtained in 2–25.5% yield. The hydrolysis of chelate compound **281** was performed with dilute sulfuric acid to afford **241** in almost quantitative yield.

**Scheme 47:**
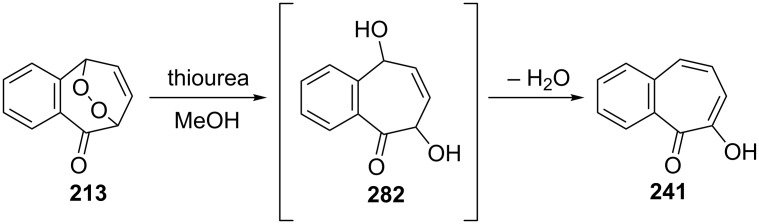
Synthesis of 7-hydroxy-2,3-benzotropone (**241**) from bicyclic endoperoxide **213**.

Bicyclic endoperoxides generated from the cycloaddition of singlet oxygen to 1,3-dienes serve as excellent synthetic precursors and have led to developments in tropone chemistry [170–172]. Taking advantage of the endoperoxide transformation, the synthesis of 7-hydroxy-2,3-benzotropone (**241**) was successfully realized by Dastan’s group (Scheme 47) [149]. Thiourea reduction of the peroxide linkage of **213** to the diol **282** and then simultaneously dehydration in situ gave the corresponding benzotropolone **241** in nearly quantitative yield.

**Scheme 48:**
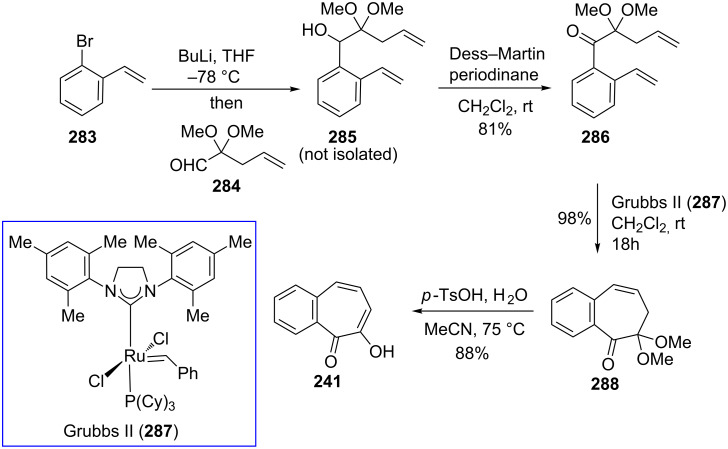
Synthesis of 7-hydroxy-2,3-benzotropone (**241**) by ring-closing metathesis.

Recently, Arican and Brückner reported the synthesis of 7-hydroxy-2,3-benzotropones by ring-closing metathesis (Scheme 48) [173]. 7-Hydroxy-2,3-benzotropone (**241**) was synthesized in four steps starting from a Br/Li exchange reaction of *o*-bromostyrene (**283**) followed by the addition of aldehyde **284** to give the benzylic alcohol **285**. Oxidation with Dess–Martin periodinane of the alcohol **285** followed by ring-closing metatheses in the presence of 1 mol % of the second generation Grubbs catalyst (**287**) gave the 5*H*-benzo[7]annulene-5,6(7*H*)-dione monoketal **288** in nearly quantitative yield. The hydrolysis of **288** with excess *p*-TsOH in aqueous acetonitrile at 75 °C for 4 h afforded the benzotropolone **241**.

**Figure 12:**
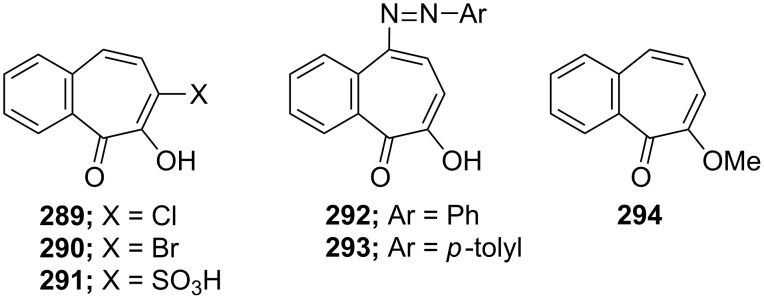
Various monosubstitution products **289**–**291** of 7-hydroxy-2,3-benzotropone (**241**).

**5.3.2. Reaction of 7-hydroxy-2,3-benzotropone (241):** Various monosubstitution products **289**–**291** of 7-hydroxy-2,3-benzotropone (**241**) were readily prepared by Nozoe’s group (Figure 12) [174]. The synthesis of bromo-derivative **290** from **241** was also reported by Cook’s group [166]. The coupling of **241** with aryldiazonium chlorides resulted mainly in the formation of 5-phenyl- and *p*-tolylazo-coupling products **292** [175] and **293** [174] (Figure 12). The reaction of **241** with diazomethane afforded methoxy-benzotropone **294** (Figure 12) [174].

**Scheme 49:**
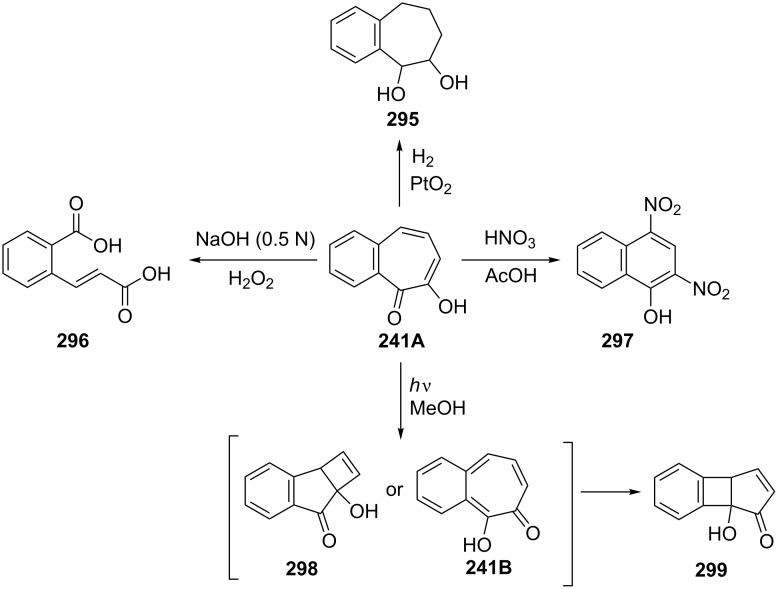
Reaction of 7-hydroxy-2,3-benzotropone (**241**) with various reagents.

Catalytic hydrogenation of **241** over Adams's catalyst (PtO_2_**^.^**H_2_) gave the diol **295** (Scheme 49) [162,165,174]. Treatment of **241** with alkaline hydrogen peroxide caused degradative fission to give *o*-carboxycinnamic acid (**296**) [165], while nitration of **241** with nitric acid in an acetic acid solution afforded 2,4-dinitro-1-naphthol (**297**) [175] (Scheme 49). Yoshioka’s group studied the photochemical behavior of benzotropolone **241** (Scheme 49) [176]. Irradiation of a dilute solution of **241A** in methanol with Pyrex-filtered light led to the formation of l-hydroxy-6,7-benzobicyclo[3.2.0]hepta-3,6-dien-2-one (**299**) in good yield as a major product. The formation of this product has been described either by the initial formation of **298** followed by rearrangement or by a mechanism with **241B** as an intermediate. Also, Aihara’s group reported excited-state intramolecular proton transfer (ESIPT) and aromaticity studies for **241A** and **241B** [177].

#### 5.4. 4-Hydroxy-2,3-benzotropone (**174**)

**Scheme 50:**
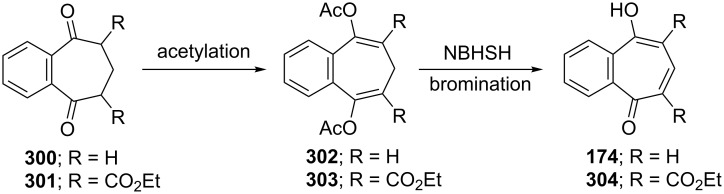
Synthesis of 4-hydroxy-2,3-benzotropones **174** and **304** from diketones **300**/**301**.

**5.4.1. Synthesis of 4-hydroxy-2,3-benzotropone (174):** Benzotropolone **174** was prepared through intermediate bis-enol acetate obtained from reaction between benzo[7]annulene-3,7-dione (**300**) and isopropenyl acetate followed by dehydrogenation using *N*-bromosuccinimide, and its properties were compared with those of benzotropolone **241A** (Scheme 50) [178]. The benzotropolone **174** could also be prepared from diester **301** in a similar way (Scheme 50) [179]. The simultaneous hydrolysis and decarboxylation of benzotropolone-diester **304** to **174** were catalyzed by NaOH.

**Scheme 51:**
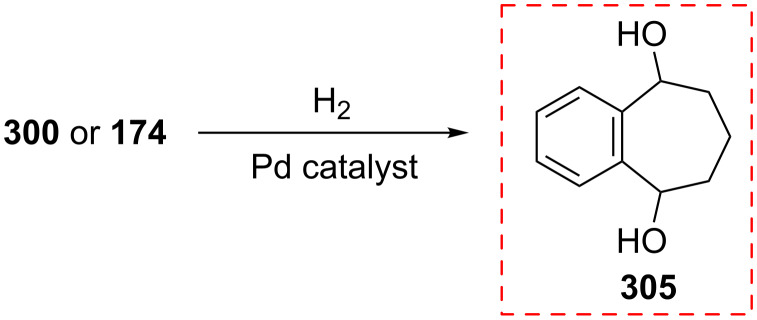
Catalytic hydrogenation of diketones **300** and **174**.

**5.4.2. Reaction of 4-hydroxy-2,3-benzotropone (174):** The structure of **174** was confirmed by the reduction of both benzotropolone **174** and diketone **300** into the diol **305** with catalytic hydrogenation (Scheme 51) [178].

### 6. Halobenzotropones

#### 6.1. Monohalobenzotropones

**Scheme 52:**
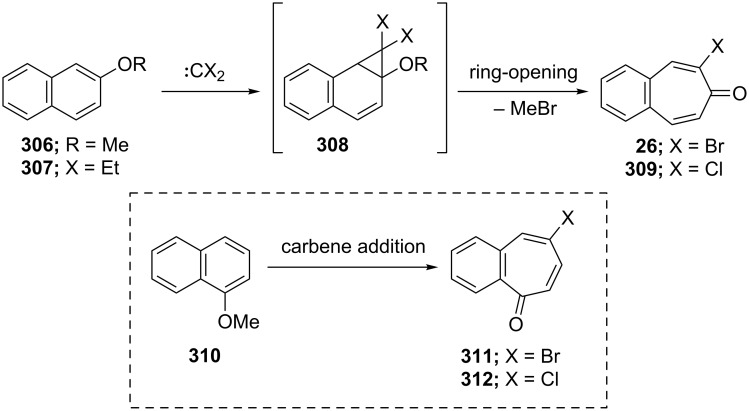
Synthesis of halo-benzotropones from alkoxy-naphthalenes **306**, **307** and **310**.

**6.1.1. One-step synthesis via dihalocarbene addition:** Probably one of the most useful methods for the synthesis of halo-benzotropones is the formation of a three-membered intermediate by addition of halocarbenes to alkoxynaphthalenes. The carbene addition step is then a simultaneous ring-opening step to give the corresponding halobenzotropone (Scheme 52). In 1969, two research groups independently reported the preparation of 2-bromo-4,5-benzotropone via adduct **308** starting from 2-methoxynaphthalene (**306)** using different dibromocarbene reagents (Scheme 52) [180–181]. The results for the synthesis of halo-benzotropones via carbene addition are shown in Table 2.

**Table 2:** Synthesis of some mono-halobenzotropones via carbene addition.

entry	alkoxy-naphthalene	carbene source	product	yield (%)	reference(s)

**1**	**306**	CHBr_3_, *t*-BuOK	**26**	11	[181]
**2**				20	[182]
**3**		PhHgCBr_3_		37	[180]
**4**		PhHgCCl_3_	**309**	37	[183]
**5**		CHCl_3_, *t*-BuOK		18	[181]
**6**		CHCl_3_, *t*-BuOK		13	[184]
**7**		Cl_3_CCO_2_Et, NaOMe		13–33	[184–185]
**8**	**307**	Cl_3_CCO_2_Et, NaOMe	**309**	66	[185]
**9**	**310**	CHBr_3_, *t*-BuOK	**311**	15–25	[180–181]
**10**		PhHgCBr_3_		33	[181]
**11**		PhHgCCl_3_	**312**	17	[183]
**12**		Cl_3_CCO_2_Et, NaOMe		11	[184]

**Figure 13:**
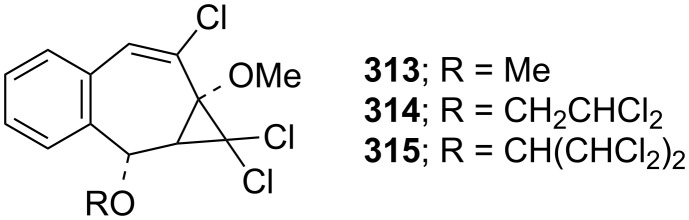
Unexpected byproducts **313**–**315** during synthesis of chlorobenzotropone **309**.

As shown in Table 2, the reported yields were extremely low. To further improve the yields of the products, different carbene sources and reaction conditions were tested. Parham’s group reported treatment of 2-methoxynaphthalene (**306**) with 0.75 equivalents of the carbene source (ethyl trichloroacetate) and sodium methoxide to give the chlorobenzotropone **309** in 13% yield [184]. Uyehara’s group also performed the same reaction by changing the ratios of the carbene sources and the base to the substrate [185]. When 7 equivalents of the carbene source and sodium methoxide were used, however, **309** was obtained in lower yield (33%) and unexpected byproducts **313–315** were isolated in 6%, 23%, and 0.2% yields, respectively (Figure 13).

**Figure 14:**
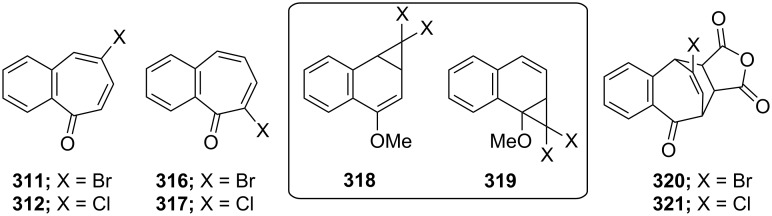
Some halobenzotropones and their cycloadducts.

Several methods for the synthesis of 7-bromo-2,3-benzotropone (**316**) via dibromocarbene addition to 1-methoxynaphthalene (**310**) were reported (Figure 14) [180–181]. However, Moncur and Grutzner repeated the reaction as described and their studies led to the structural revision of the previously published structure of 7-bromo-2,3-benzotropone (**316**) to that of 5-bromo-2,3-benzotropone (**311**, Scheme 52, Figure 14) [186]. The structure of **311** has also been confirmed by independent extensive experiments and NMR data [182,187]. The chloro-derivative **312** was synthesized from the addition of dichlorocarbene to **310** in the same manner [187]. The results indicated that the dihalocarbenes prefer the addition of the 3,4-double bond rather than the 1,2-double bond to 1-methoxynaphthalene (**310**). The position of the halogen substituent in **311** and **312** was also determined by the cycloadducts **320** and **321** between 5-halo-2,3-benzotropones and maleic anhydride (Figure 14) [185,187].

**Scheme 53:**
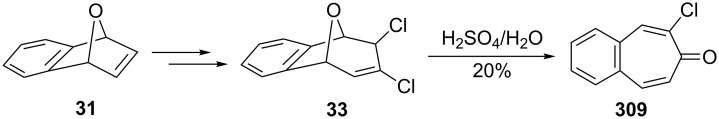
Multisep synthesis of 2-chlorobenzotropone **309**.

**6.1.2. Multistep synthesis via dihalocarbene addition:** As shown in Scheme 6, the synthesis of the bicyclic ring **33** from the dichlorocarbene adduct of oxobenzonorbornadiene **31** has also been reported by Ranken’s group in two steps [53]. Hydrolysis of **33** in water under acidic conditions led to 2-chlorobenzotropone **309** in 20% yield (Scheme 53) [53].

**Scheme 54:**
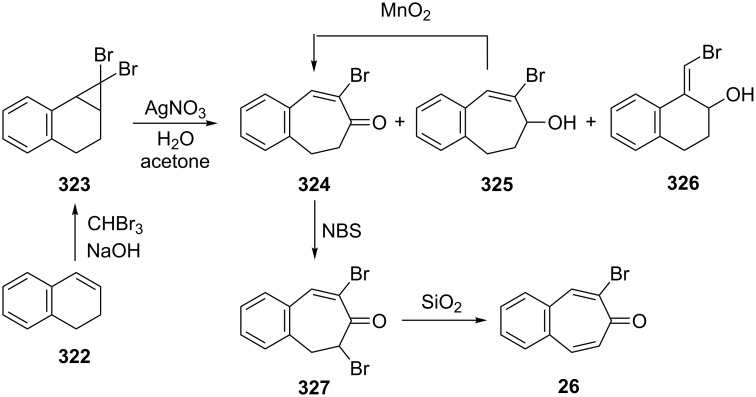
A multistep synthesis of 2-bromo-benzotropone **26**.

A multistep method for the synthesis of 2-bromobenzotropone **26** starting from dihydronaphthalene (**322**) was also realized by Balci’s group (Scheme 54) [188]. After addition of dibromocarbene to **322**, the obtained dibromocyclopropane **323** was submitted to silver ion-catalyzed ring expansion/hydrolysis in aqueous acetone (autoclave, 7.5–8.5 atm, 120–124 °C) to yield a mixture of products, **324**, **325**, and **326** in 53%, 3%, and 8% yields, respectively. Bromo-alcohol **325** can be converted readily to unsaturated ketone **324** by MnO_2_ oxidation. Finally, the NBS-mediated bromination of **324** followed by dehydrobromination on silica gel led to the corresponding bromobenzotropone **26** in 80% yield.

**Scheme 55:**
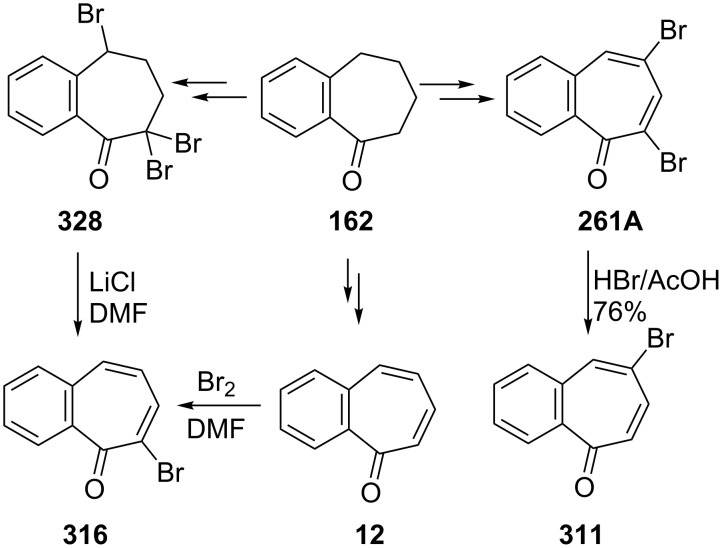
A multistep synthesis of bromo-2,3-benzotropones **311** and **316**.

**6.1.3. Synthesis using benzosuberone:** Jones’ groups reported two synthetic ways for obtaining 7-bromo-2,3-benzotropone (**316**) starting from benzosuberone (**162**) (Scheme 55) [135–136]. As shown in Scheme 30, brominations of **162** were investigated using both molecular bromine and NBS conditions. On the other hand, excess bromination of benzosuberone **162** with NBS resulted in the formation of tribromide **328**. Treatment of tribromide **328** with LiCl in DMF yielded 7-bromo-2,3-benzotropone (**316**) in high yield. The results have shown that lithium chloride can be used as a mild dehydrobromination base to obtain the corresponding tropones from the multihalo-benzosuberones. Alternatively, 7-bromo-2,3-benzotropone (**316**) was prepared in situ from benzotropone **12** and bromine in DMF followed by dehydrobromination (Scheme 55). The synthesis of benzotropone **12** from benzosuberone (**162**) is shown in Scheme 30. Benzosuberone (**162**) was also used as starting material for the synthesis of 5-bromo-2,3-benzotropone (**311**) (Scheme 55) [187,189].

**Scheme 56:**
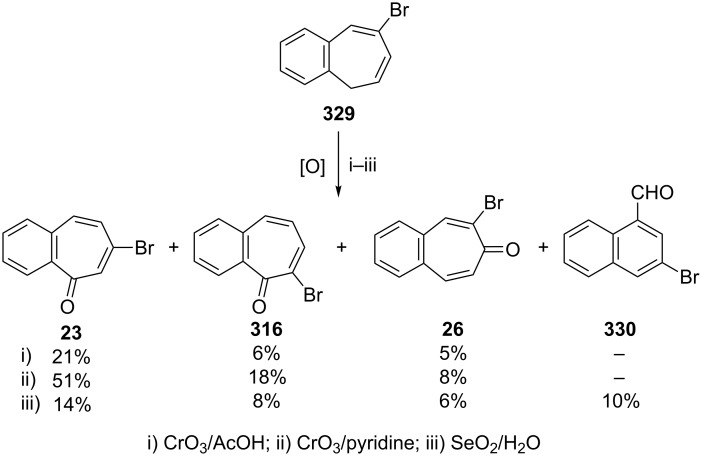
Oxidation reactions of 8-bromo-5*H*-benzo[7]annulene (**329**) with some oxidants.

**6.1.4. Synthesis via oxidation:** As shown in Scheme 4, bromobenzotropones **23** and **26** were obtained and characterized during the oxidation of both benzylic and allylic positions in 7-bromo-5*H*-benzo[7]annulene (**22**) [52]. To the best of those authors’ knowledge, this is the first synthesis of **23**. With the reaction conditions established, Balci’s group next turned their attention to evaluating the scope and limitations of the oxidation reaction with different types of benzo[7]annulene (Scheme 56) [190]. Thus 8-bromo-5*H*-benzo[7]annulene (**329**) was oxidized with different oxidants to give a mixture of bromobenzotropones such as **23**, **316**, and **26**. Formation of naphthaldehyde derivative **330** was also reported by SeO_2_-oxidation reaction (Scheme 56).

**Scheme 57:**
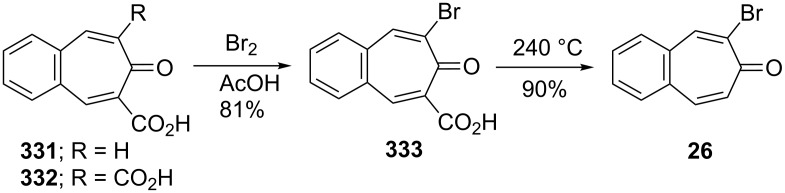
Synthesis of 2-bromo-4,5-benzotropone (**26**).

**6.1.5. Synthesis via benzotropone precursors:** Suzuki reported the formation and reactions of 2-carboxylic acid and 2,7-dicarboxylic acid derivatives **331** and **332** of 4,5-benzotropone (Scheme 57) [191]. 2-Carboxylic acid-substituted 4,5-benzotropone **331** was converted to 2-bromo-4,5-benzotropone (**26**) via bromination in acetic acid followed by decarboxylation at 240 °C.

**Scheme 58:**
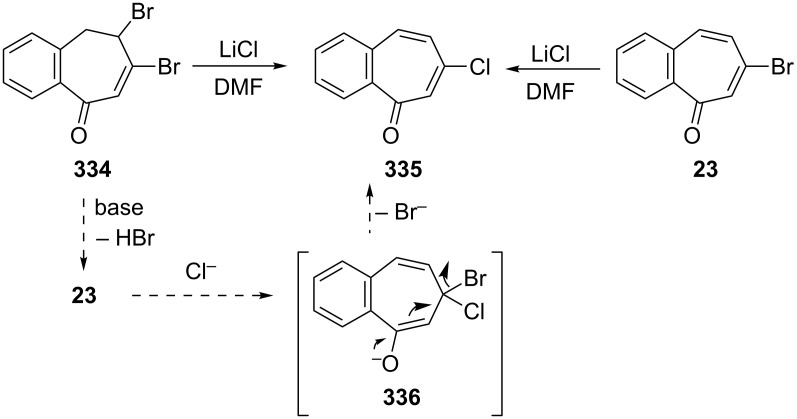
Synthesis of 6-chloro-2,3-benzotropone (**335**) using LiCl and proposed intermediate **336**.

The first synthetic methods for 6-chloro-2,3-benzotropone (**335**) were presented by Balci’s group (Scheme 58) [52]. When dibromide **334** was dehydrobrominated by lithium chloride in *N,N*-dimethylformamide, the chloro derivative **335** was formed as a sole product without any other halo derivatives. Independently, the reaction of 6-bromo-2,3-benzotropone (**23**) with lithium chloride under the same reaction conditions gave 6-chloro-2,3-benzotropone (**335**) in 96% yield. The proposed mechanism involves the intermediate **336** formed by Michael addition of a chloride ion to the β-position of the carbonyl group followed by the elimination of a bromide ion as a better leaving group.

#### 6.2. Reactions of monohalo-benzotropones

**Scheme 59:**
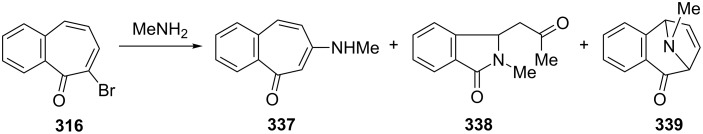
Reaction of 7-bromo-2,3-benzotropone (**316**) with methylamine.

**6.2.1. Reactions with nucleophiles:** Crabbé’s group reported the reactions of 7-bromo-2,3-benzotropone (**316**) with several primary and secondary amines (Scheme 59) [192]. Amines such as ammonia, dimethylamine, and morpholine analogous amines afforded the corresponding *cine*-substitution products such as **337**, whereas the reactions of compound **316** with various amines such as methylamine, ethylamine isopropylamine, and ethanolamine gave aromatic lactams such as **338** and tricyclic amino derivatives as **339**, in addition to the desired *cine*-substitution products, under similar reaction conditions. It was proposed that the aromatic lactam was formed via cleavage of the troponoid ring. The tricyclic ring was derived by a sequence of 1,6-addition reaction of the amine to the tropone and intramolecular displacement of the bromine by an attack from the nitrogen.

**Scheme 60:**
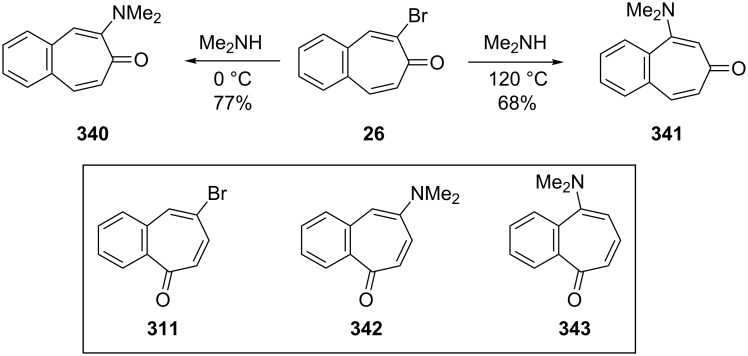
Reactions of bromo-2,3-benzotropones **26** and **311** with dimethylamine.

Namboothiri and Balasubrahmanyam showed that the *ipso*/*cine* regioselectivity in the amination of some bromobenzotropones **26** and **311** was dependent upon the temperature at which the reaction was conducted (Scheme 60) [182]. The reactions of 2-bromo-4,5-benzotropone (**26**) and 5-bromo-2,3-benzotropone (**311**) with dimethylamine were carried out at a high temperature and *ipso*-products (**340** and **342**) were more favorable than *cine*-products (**341** and **343**).

**Scheme 61:**
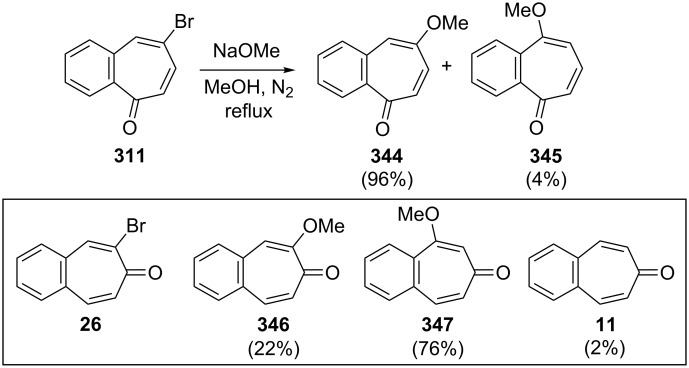
Reactions of bromobenzotropones **311** and **26** with NaOMe.

**Scheme 62:**
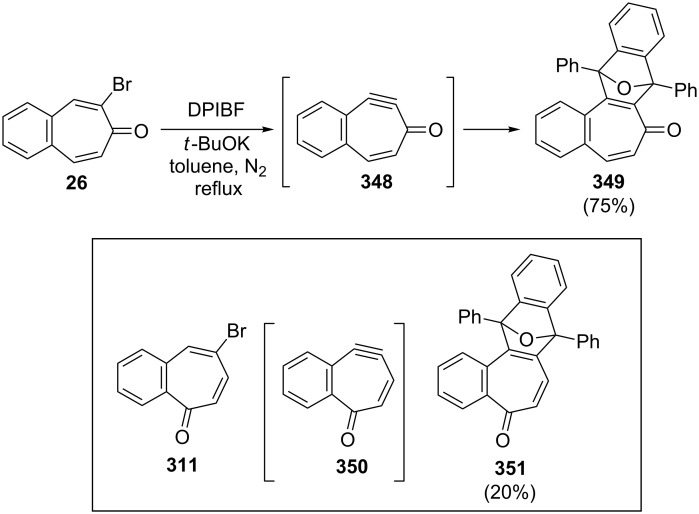
Reactions of bromobenzotropones **26** and **312** with *t*-BuOK in the presence of DPIBF.

Namboothiri and Balasubrahmanyam also investigated transformations in bromo- and alkoxybenzotropones (Scheme 61) [182]. The treatment of bromobenzotropones **26** and **311** with sodium methoxide in methanol under reflux led to a mixture of *ipso* and *cine* products. While the *ipso* product **344** in the case of **311** is dramatically favored over the *cine* product **345** (96:4), the *ipso*/*cine* ratio **346**/**347** in the case of **26** is 22:76. However, a small (2%) amount of 4,5-benzotropone (**11**) was formed under these conditions via presumably reductive removal of the bromine. In addition, a trapping experiment with 1,3-diphenylisobenzofuran (DPIBF) furnished evidence for the formation of benzodehydrotropones **348** and **350**, generated by the reaction of **26** and **311** with *t*-BuOK (Scheme 62).

**Scheme 63:**
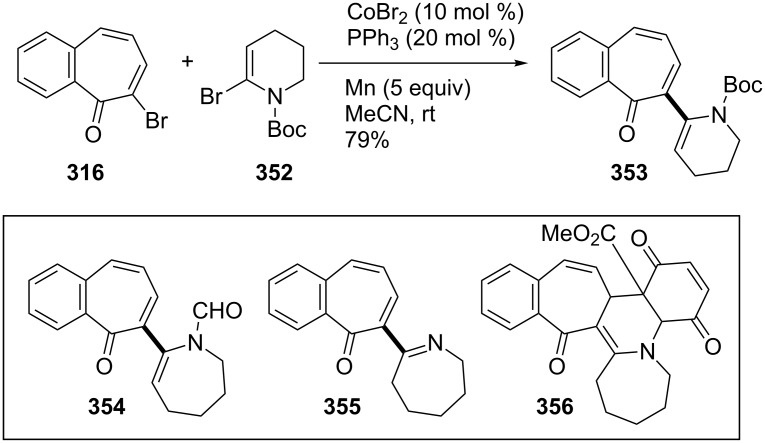
Cobalt-catalyzed reductive cross-couplings of 7-bromo-2,3-benzotropone (**316**) with cyclic α-bromo enamides.

**6.2.2. Miscellaneous reactions:** The direct functionalization of important motifs such as benzotropones, cycloheptenones, azepanes, and piperidines is of ubiquitous importance. In 2015, Beng’s group focused on the cobalt-catalyzed reductive cross-coupling of versatile α-bromo enones with cyclic α-bromo enamides under mild conditions (Scheme 63) [193]. The coupling of bromo enecarbamate **352** and 7-bromo-2,3-benzotropone (**316**) was efficiently accomplished at room temperature using the conditions described in Scheme 61. The coupling product **354** was also prepared by this method. Treatment of imino diene **355** with the corresponding ester-quinone as an activated dienophile resulted in the formation of the highly functionalized pentacyclic **356** (Scheme 63).

**Figure 15:**
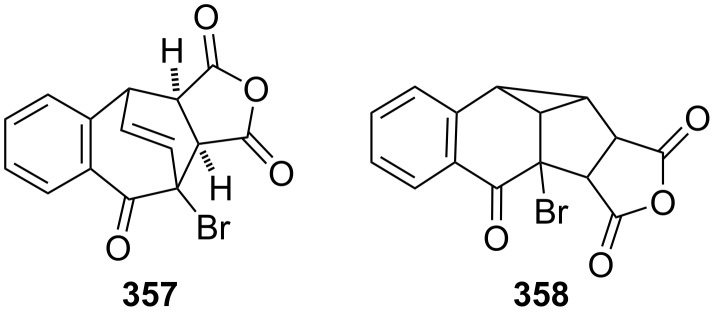
Cycloadduct **357** and its di-π-methane rearrangement product **358**.

As illustrated in Scheme 35 and Figure 14, benzotropones can be used to afford cycloadducts and their photochemical products. In this context, the cycloaddition of 7-bromo-2,3-benzotropone (**316**) to maleic anhydride was reported by Hassner’s group (Figure 15) [148]. The direct and sensitized photolysis of the cycloadduct **357** afforded di-π-methane rearrangement product **358**, which was confirmed by X-ray diffraction.

**Scheme 64:**
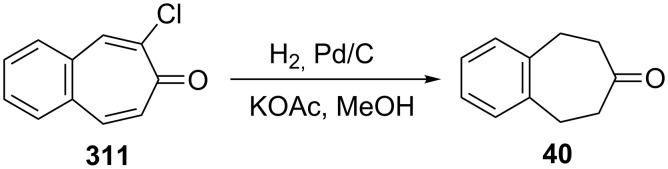
Catalytic hydrogenation of 2-chloro-4,5-benzotropone (**311**).

Simple and practical routes to 5,6,8,9-tetrahydro-7*H*-benzo[7]annulen-7-one (**40**) were reported by Uyehara’s group [184]. Catalytic hydrogenation of 2-chloro-4,5-benzotropone (**311**) with 5% palladium on activated charcoal in methanol gave the ketone **40** in 97% yield (Scheme 64).

#### 6.3. Dibromobenzotropones

**Scheme 65:**
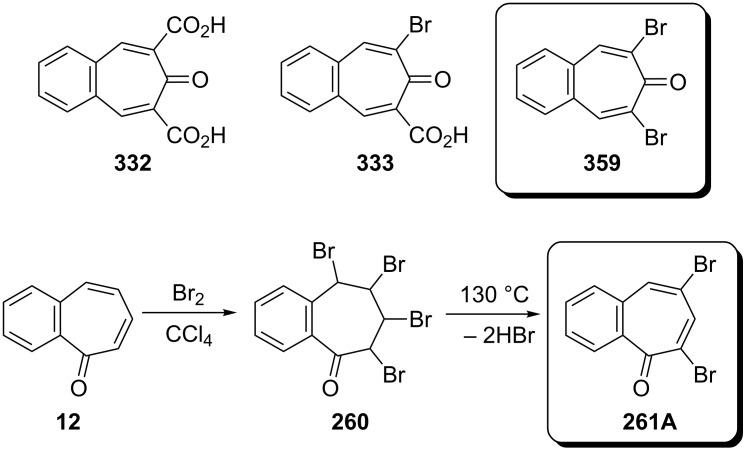
Synthesis of dibromo-benzotropones from benzotropones.

**6.3.1. Synthesis from benzotropones:** Decarboxylation of both diacid- **332** and monoacid-benzotropone **333** by Hunsdiecker–Simonini reaction gave 2,7-dibromo-4,5-benzotropone (**359**) in 31% and 12% yield, respectively (Scheme 65) [191]. Bromination of 2,3-benzotropone (**12**) afforded tetrabromide **260** only, which underwent dehydrobromination to yield 5,7-dibromo-2,3-benzotropone (**261A,** Scheme 65) [134].

**Scheme 66:**
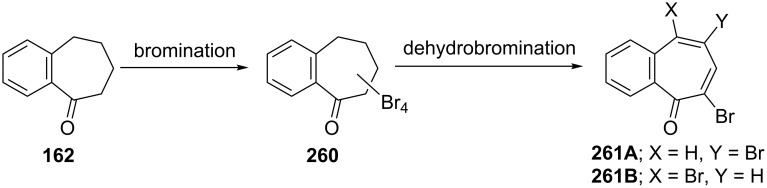
Bromination/dehydrobromination of benzosuberone (**162**).

**6.3.2. Synthesis from benzosuberone:** An alternative protocol for the preparation of the dibromobenzotropones **261A** and **261B** was bromination/dehydrobromination starting from benzosuberone (**162**) (Scheme 66) [134,163,189].

**Scheme 67:**
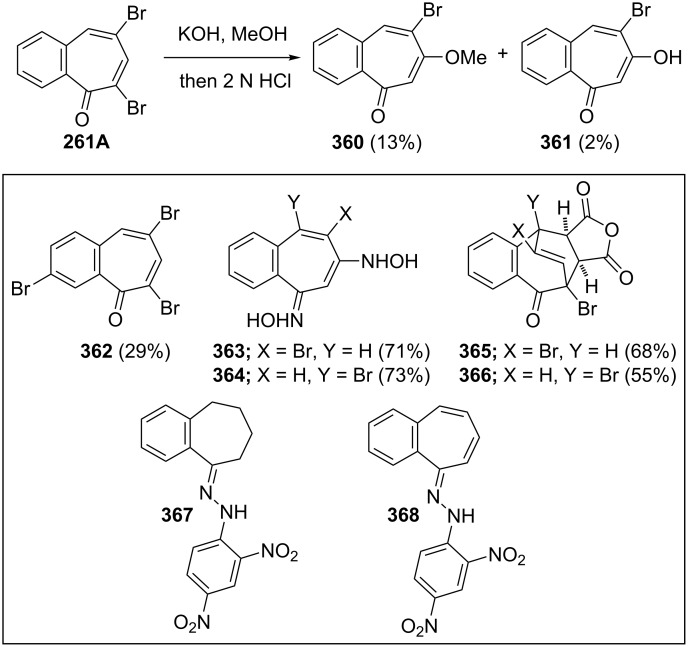
Some transformations of isomeric dibromo-benzotropones **261A**/**B**.

**6.3.3. Reactions of dibromobenzotropones:** The transformations of isomeric dibromo-benzotropones **261A** and **261B** are summarized in Scheme 67 [163,189]. Dibromo-benzotropone **261A** was treated with KOH in methanol at room temperature for 24 h followed by acidification using HCl to yield 6-methoxy- and 6-hydroxybenzotropones **360** and **361** and an uncharacterized product. Tribromide **362** was prepared by treating **162** with refluxing bromine. Treatment of dibromobenzotropones with hydroxylamine caused a *cine*-reaction to give oximes **363** and **364**. The Diels–Alder adducts **365** and **366** of **261A** and **261B** with maleic anhydride were used to elucidate the position of the bromo substituents. The reduction of **261B** in acetic acid with 4 mol equivalent of hydrogen in the presence of 5% palladium-on-charcoal and anhydrous sodium acetate, and by subsequent treatment with 2,4-dinitrophenylhydrazine, resulted in the formation of hydrazone **367**. Moreover, the hydrazone **368** was prepared in an analogous manner using 2 equivalents of hydrogen.

#### 6.4. Halobenzotropolones

**Scheme 68:**
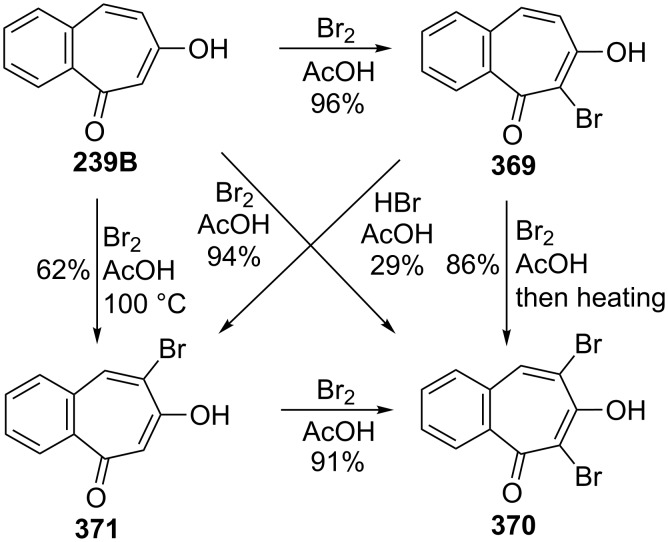
Transformations of benzotropolone **239B** to halobenzotropolones **369**–**371**.

**Figure 16:**
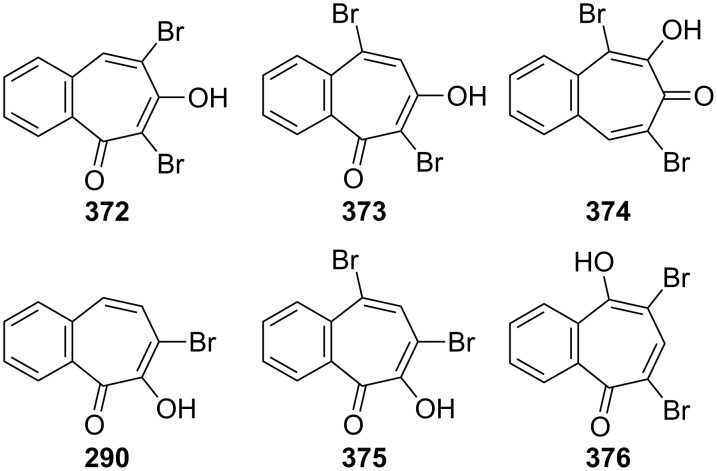
Bromobenzotropolones **372**–**376** and **290** prepared via bromination/dehydrobromination strategy.

**6.4.1. Synthesis of halobenzotropolones:** The benzotropolones undergo electrophilic substitution in the form of halogenation and their reactions towards halogens are similar. Hoshino and Ebine reported the formation and reaction of bromo derivatives **369** and **370** of benzotropolone **239B** with bromine in acetic acid under various conditions (Scheme 68) [194]. Bromobenzotropolones **372**–**376** and **290** were also synthesized by bromination/dehydrobromination of the corresponding benzotropolenes (Figure 16) [165,179,194–197].

**Scheme 69:**
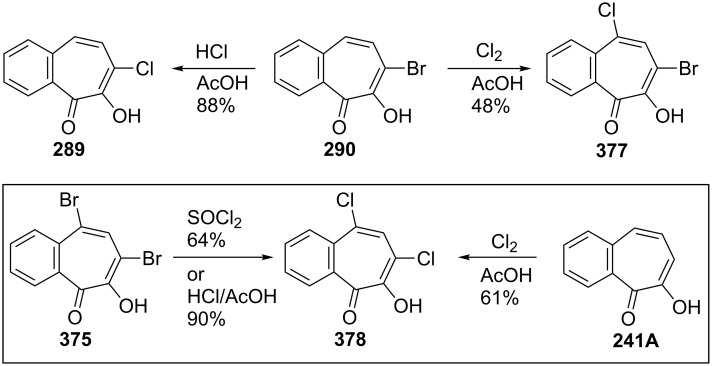
Synthesis of some halobenzotropolones **289**, **377** and **378**.

**Figure 17:**
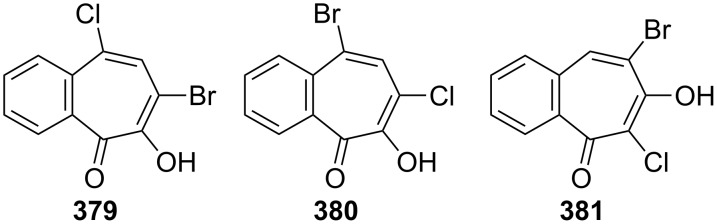
Bromo-chloro-derivatives **379**–**381** prepared via chlorination.

**Scheme 70:**
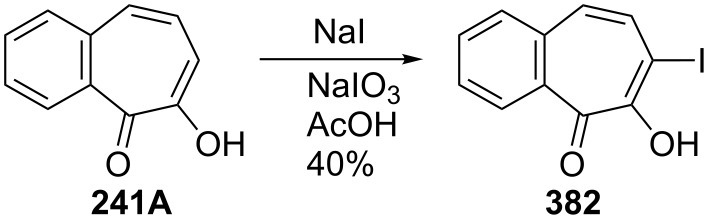
Synthesis of 7-iodo-3,4-benzotropolone (**382**).

A short communication describing how 3,4-benzotropolone (**241A**) can be chlorinated to yield monochloro-3,4-benzotropolone was presented by Nozoe’s group [174]. Ebine studied in more detail the chlorination and iodination of **241A** (Scheme 69, Scheme 70 and Figure 17) [194,198]. Treatment of the benzotropolone **241A** with one or two equivalents of chlorine in acetic acid afforded 5,7-dichloro-3,4-benzotropolone (**378**) in low to fair yield. When reacted with a concentrated hydrochloric acid, both **290** and **375** underwent halogen exchange to give **289** and **378**, respectively. A similar substitution was also observed when **375** was reacted with thionyl chloride. Bromo-chloro-3,4-benzotropolones **379**–**381** were prepared using similar procedures (Figure 17). 7-Iodo-3,4-benzotropolone (**382**) was also obtained in 40% yield by the reaction between benzotropolone **241A** and sodium iodide/sodium iodate in acetic acid (Scheme 70) [195].

**Scheme 71:**
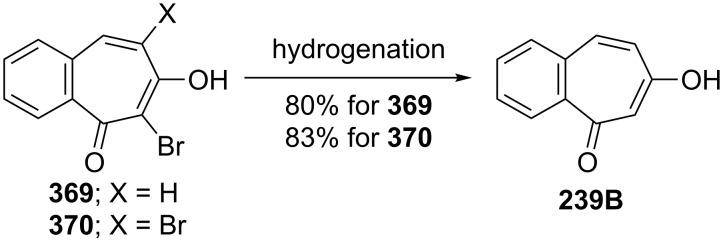
Hydrogenation of bromobenzotropolones **369** and **370**.

**Scheme 72:**
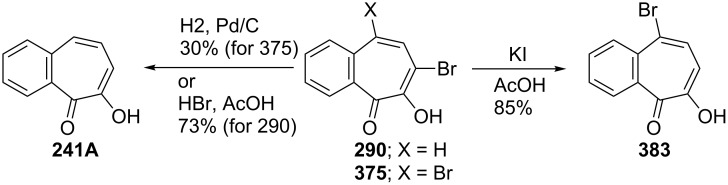
Debromination reactions of mono- and dibromides **290** and **375**.

**6.4.2. Reaction of halobenzotropolones: Dehalogenation of halobenzotropolones:** Hoshino and Ebine reported that palladium-catalyzed hydrogenation of bromobenzotroponoids **369** and **370** resulted in debromination of halogen atoms to give **239B** as depicted in Scheme 71 [194]. However, hydrogenation of **375** gave **241A** in low yield (Scheme 72) [195]. Debromination of monobromide **290** with hydrobromic acid in acetic acid afforded **241A** in 73% yield, whereas the reaction of dibromide **375** under the same conditions provided monobromide **383** in 85% yield as the debromination product (Scheme 72) [198].

**Figure 18:**
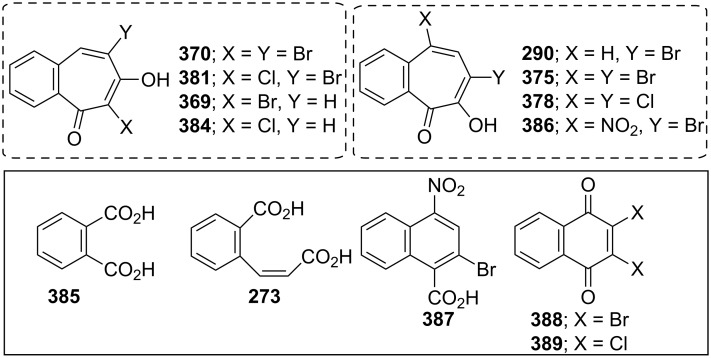
Nitratation and oxidation products of some halobenzotropolenes.

**Oxidation of halobenzotropolones:** Oxidation reactions of halo-benzotropolones were often used to determine the structures of benzotroponoids [194,198–199]. For clarification of the positions of substituents in the final compounds, Ebine reported that oxidation of dihalo-benzotropones **370** and **381** with alkaline hydrogen peroxide gave phthalic acid (**385**) in 40% and 35% yields, respectively, while that of monohalobenzotropolenes **369**, **384**, and **290** afforded *o*-carboxycinnamic acid (**273**) in 47%, 33%, and 33% yields, respectively (Figure 18) [194]. Bromobenzotropolones **290** and **375** were nitrated in acetic acid to yield the same nitration product **386** in 29% and 16% yields (Figure 18) [199]. 5-Nitro-7-bromo-3,4-benzotropolone (**386**) rearranged to 2-bromo-4-nitro-1-naphthoic acid (**387**) in 80% yield with alkali (Figure 18). When reacted with exhaustive bromination (or chlorination) in acetic acid, dibromobenzotropolone **375** gave 2,3-dibromo-1,4-naphthoquinones (**388**) in 81% yield (or **389** in 35% yield) as an unexpected product (Figure 18). The nitration of **375** in concentrated sulfuric acid also produced the corresponding **388** in 29% yield (Figure 18). A reaction of 5,7-dichloro-3,4-benzotropolone (**378**) under the same conditions gave dichloronaphthoquinone **389** in 16% yield (Figure 18). The author proposed possible tentative mechanisms for the formation of naphthoquinones.

**Scheme 73:**
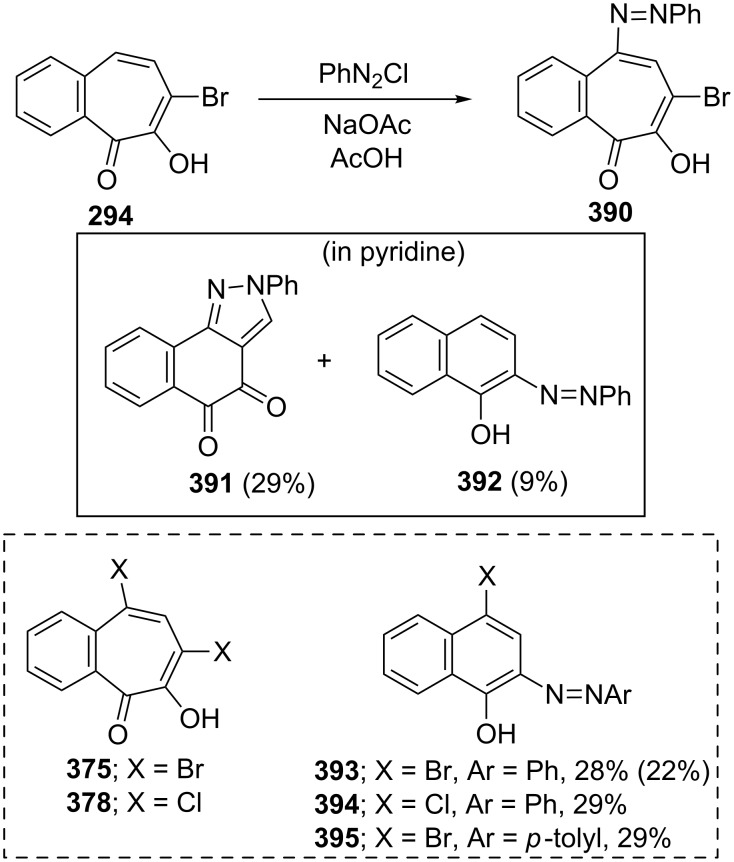
Azo-coupling reactions of some halobenzotropolones **294**, **375** and **378**.

**Azo-coupling reaction of halo-benzotropolones:** The azo-coupling reaction of 7-bromobenzotropolones **294** with diazonium cations, which are generated by treatment of aromatic amines with nitrous acid and a stronger mineral acid in acetic acid, resulted in 5-phenylazo-7-bromo-3,4-benzotropolone (**390**) in 28% yield (Scheme 73) [175]. However, when the same reaction was carried out in a pyridine solution, the formation of rearrangement products **391** and **392** in 29% and 9% yields was reported (Scheme 73). Azo-coupling reactions of 5,7-dihalo-3,4-benzotropolones **375** and **378** under similar conditions provided the corresponding naphthols **393**–**395** in low yields (Scheme 73). The possible courses for the formation of coupling products were discussed [175].

### 7. Dibenzotropones

**Figure 19:**
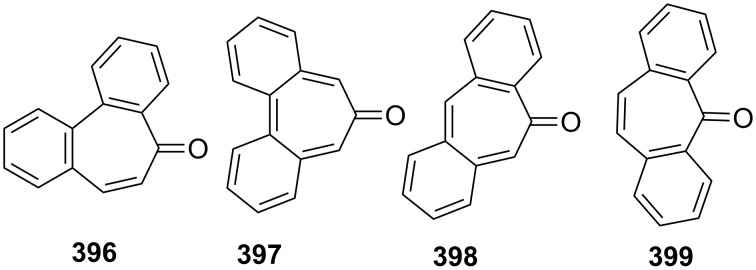
Four possible isomers of dibenzotropones **396**–**399**.

There are four possible dibenzotropone isomers: 2,3;4,5-dibenzotropone (**396**), 3,4;5,6-dibenzotropone (**397**), 2,3;5,6-dibenzotropone (**398**), and 2,3;6,7-dibenzotropone (**399,** Figure 19). We reported comprehensive syntheses and applications of dibenzosuberenones [45]. Thus, this section does not cover the chemistry of dibenzotropones.

### 8. Tribenzotropone (**400**)

**Figure 20:**
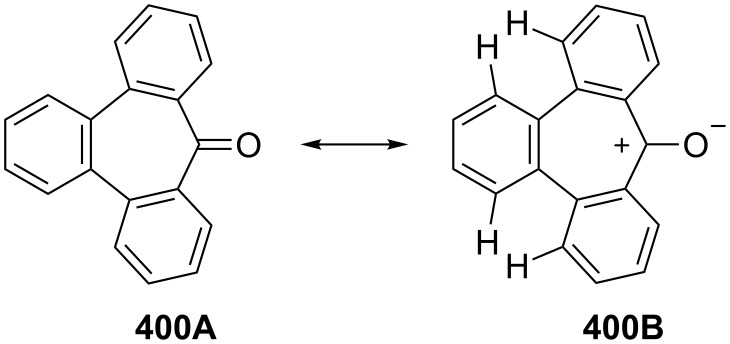
Resonance structures of tribenzotropone (**400**).

Tribenzotropone, or 9*H*-tribenzo[*a,c,e*][7]annulen-9-one (**400A**), has a tetracyclic structure, consisting of a seven-membered ring fused to benzene rings (Figure 20). Based on experimental observations, it is suggested that tribenzotropone (**400**) shows structural resistance against planarity arising from an angular strain of a planar 7-membered ring as well as the unfavorable steric interactions between the *ortho*-hydrogen atoms (Figure 20) [200]. As a measure of the characteristics of tropone, the calculated circuit resonance energies show that tribenzotropone (**400**) among the other benzotropones has a small circuit resonance energy associated with the number of benzene rings [155]. The charge density for the corresponding uniform reference frame of **400** shows that the oxygen atom occupies the site of the largest charge density.

#### 8.1. Synthesis of tribenzotropone

**Scheme 74:**
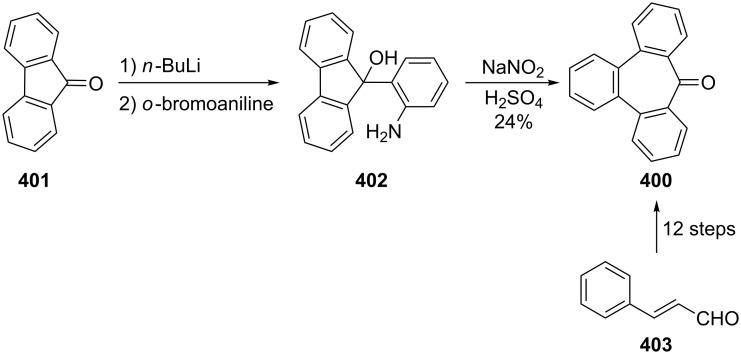
Two synthetic pathways for tribenzotropone (**400**).

**Scheme 75:**
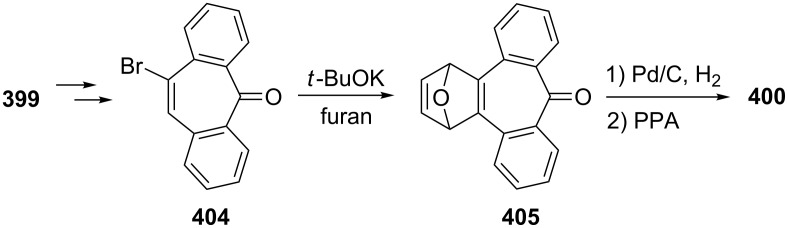
Synthesis of tribenzotropone (**400**) from dibenzotropone **399**.

The first synthesis of tribenzotropone (**400**) was simultaneously reported by two groups in 1957. Stiles’ group reported the synthesis of **400** in 24% yield via the rearrangement of the diazonium salt of 9-(2-aminophenyl)-9*H*-fluoren-9-ol (**402**) in two steps (Scheme 74) [200]. A multistep preparation with difficulties or poor yields of **400** was reported by Bergmann’s group starting from cycloaddition of butadiene and cinnamaldehyde (**403**) in 12 steps (Scheme 74) [201]. Moreover, Diels–Alder trapping with furan of an alkyne derivative from benzotropone **399** followed by catalytic hydrogenation and polyphosphoric acid (PPA)-assisted dehydration steps provided an excellent approach to the synthesis of tribenzotropone (**400**) in a 31% overall yield over five steps (Scheme 75) [202]. Wan’s group also reported the deoxygenation with Fe_2_(CO)_9_ of the cycloadduct **404** to **400** [203].

**Scheme 76:**
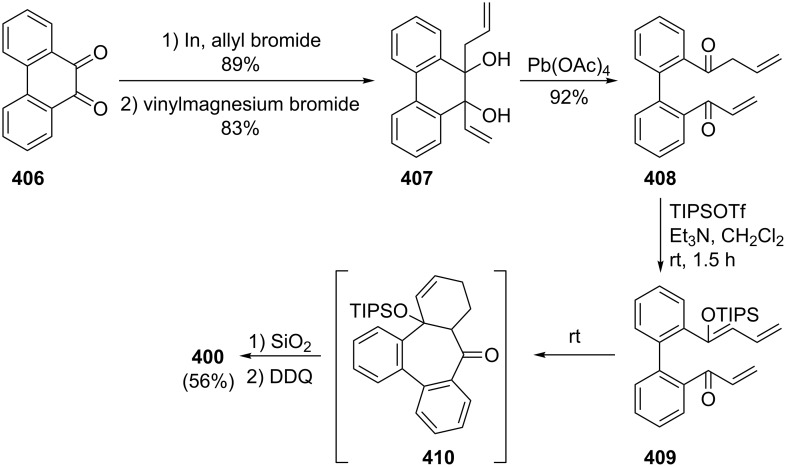
Synthesis of tribenzotropone (**400**) from 9,10-phenanthraquinone (**406**).

Koo’s group reported a challenging method for the synthesis of **400** in 38% overall yield by ring-expansion method as a key step starting from readily available 9,10-phenanthraquinone (**406**, Scheme 76) [204]. A mild and selective indium-mediated nucleophilic addition of allyl bromide followed by the addition of vinylmagnesium bromide led to the formation of diol **407** with allyl and vinyl substituents, which underwent an oxidative ring-opening reaction to form diketone **408**. Then the reaction of **408** with triisopropyl triflate (TIPSOTf) in the presence of triethylamine afforded the desired silyl enol ether **409**, which contains the required electron-rich diene and electron-deficient dienophile units for intramolecular cycloaddition. Unexpectedly, the intramolecular Diels–Alder reaction of **409** at room temperature followed by filtration from silica gel gave an inseparable mixture of tribenzotropone (**400**) and dihydro analogue of **400**. The crude mixture was reacted with 2,3-dichloro-5,6-dicyano-1,4-benzoquinone in order to complete the oxidation (DDQ).

**Scheme 77:**
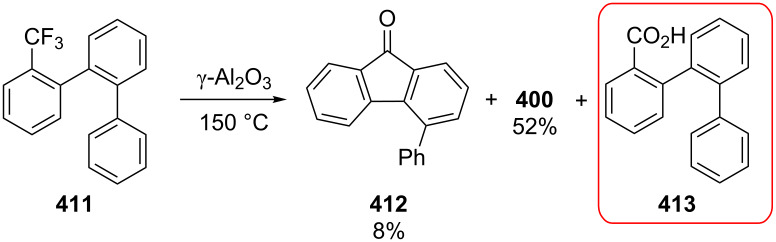
Synthesis of tribenzotropone (**400**) from trifluoromethyl-substituted arene **411**.

Papaianina and Amsharaov demonstrated that thermally activated γ-aluminum oxide can be very effective for C–F bond activation in trifluoromethyl-substituted arenes to yield either cyclic ketones or the respective carboxylic acids in good to excellent yields (Scheme 77) [205]. The condensation of trifluoromethyl-substituted arene **411** on activated alumina at 150 °C resulted in the formation of the intramolecular Friedel–Crafts products **400** (52% yield) and **412** (8% yield), whereas formation of the possible acid **413** was not observed. The prevention of side reactions and the regiochemistry of the process were attributed to the confined space of alumina pores. Furthermore, the non-activated alumina-mediated hydrolysis of **412** at 200 °C afforded *o*-terphenyl-2-carboxylic acid (**413**) in close to quantitative yield. A presumable mechanism was also proposed for the formation of acylation and hydrolysis products with C–F activation in trifluoromethylated arenes in alumina nanopores.

#### 8.2. Reactions of tribenzotropone (**400**)

**Figure 21:**
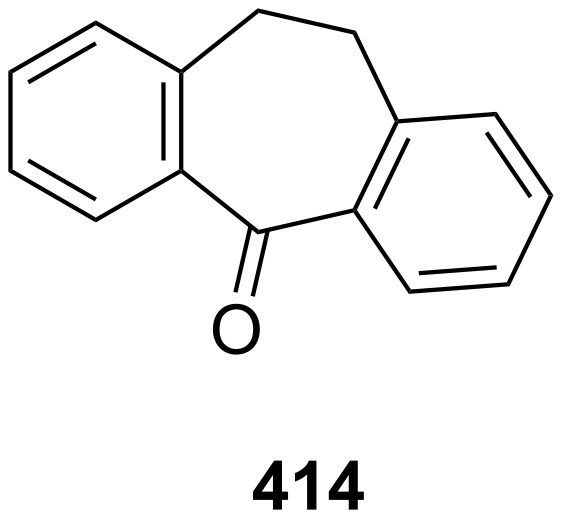
Dibenzosuberone (**414**).

Herold’s group reported ESR and ENDOR/TRIPLE resonance studies of ion pairs derived from the reduction of tribenzotropone (**400)**, dibenzotropone **399**, and dibenzosuberone **414** (Figure 21) with different alkali metals, which may be evidence of the existence of three different stereoisomers [206]. The INDO calculations of the spin densities at the lithium cation also supported the geometries proposed for the three stereoisomers.

**Figure 22:**
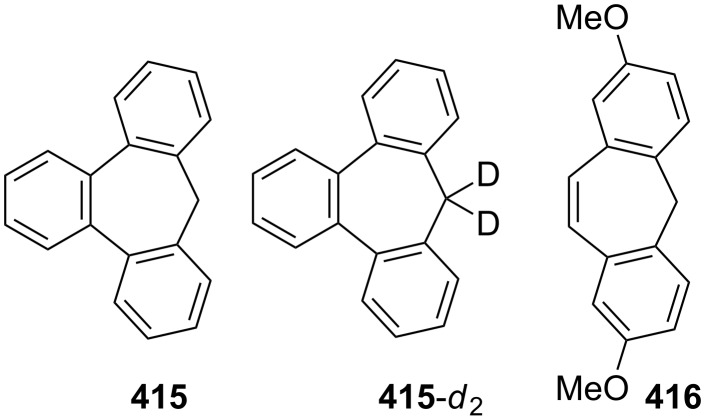
Reduction products **415** and **416** of tribenzotropone (**400**).

**Figure 23:**
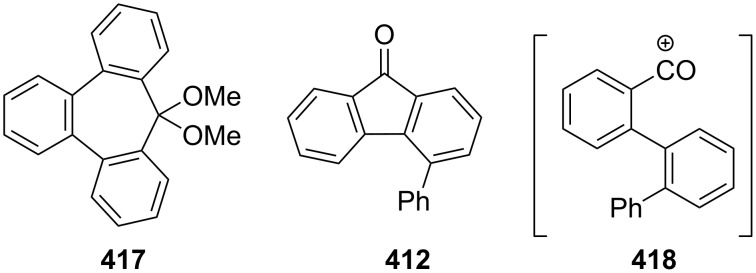
Structures of tribenzotropone dimethyl ketal **417** and 4-phenylfluorenone (**412**) and proposed intermediate **418**.

An experimental study on the excited-state carbon acidity of several dibenzosuberene derivatives was reported by Wan’s group (Figure 22) [203]. To this end, tribenzotropone (**400**) from the selected substrate was reduced with both LiAlH_4_ (with AlCl_3_) and LiAlD_4_ (with AlCl_3_) to give **415** and **415**-*d*_2_, respectively. The detectable deuterium (protium) incorporation for photolysis of **415** in D_2_O–MeCN (1:1) (or **415**-*d*_2_ in H_2_O–MeCN (1:1)) was not observed. Photolysis of **416** under similar conditions resulted in mono- (21%) and dideuterium (3%) incorporation at the methylenic position. These results were explained by the fact that benzannelation of the vinyl moiety affects the excited-state carbon acidity for **415** (or **415**-*d*_2_). Subsequent photolysis of **415** using 1 M NaOD/EtOD gave mono- and dideuterium exchange products **415**-*d* (15%) and **415**-*d*_2_ (3%). 9*H*-Tribenzo[*a*,*c*,*e*][7]annulene (**415**) as one of the model compounds for conformational studies with dynamic NMR was prepared from tribenzotropone (**400**) via Wolff–Kishner reduction (Figure 22) [207]. Tochtermann’s group prepared tribenzotropone dimethyl ketal **417** from **400** and studied the conversion of the boat form of the 7-membered ring by means of the NMR spectra (Figure 22) [208]. The free activation enthalpy for **417** was 23 kcal/mol. The treatment of **400** with polyphosphoric acid (PPA) at 200 °C yielded 4-phenylfluorenone (**412**) in 60% yield via a proposed intermediate, **418** (Figure 23) [202].

**Figure 24:**
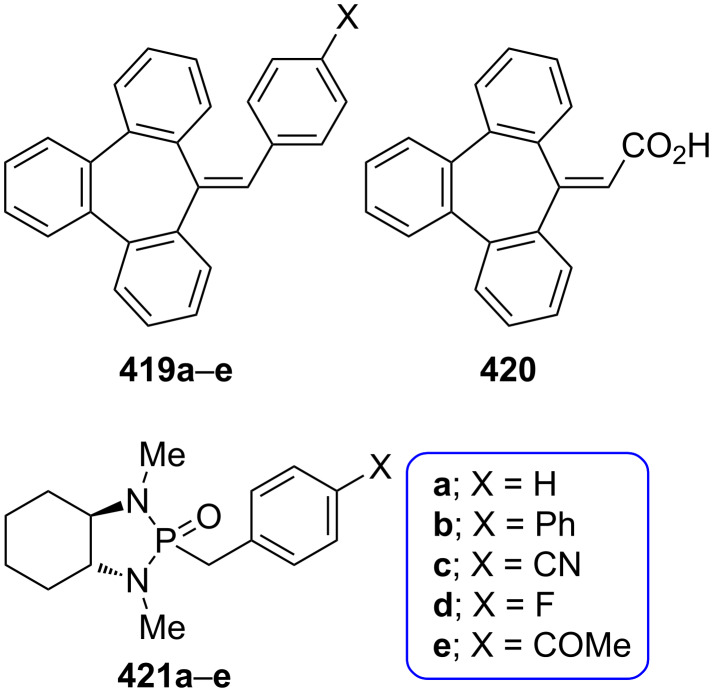
Structures of benzylidene- and methylene-9*H*-tribenzo[*a*,*c*,*e*][7]annulenes **419** and **420** and chiral phosphonamides **421**.

Bergmann and Klein reported the synthesis of the condensation product **419a** by the reaction of **400** with benzylmagnesium chloride (Figure 24) [201]. The UV absorption spectrum for **419a** was measured and it was evaluated that **419a** has no fulvenic properties. Later, Tochtermann’s group reported the synthesis of the racemic 9-methylene-9*H*-tribenzo[*a*,*c*,*e*][7]annulenes such as **420** via Wittig reaction followed by carboxylation of vinylic bromide using lithium/carbon dioxide (Figure 24) [209]. The classical resolution of the vinyl carboxylic acids as its brucine salt was also studied and the thermal racemization barrier was 31 kcal/mol at 139 °C. Udayakumar and Schuster were the first to show the direct asymmetric synthesis of a series of 9-benzylidene-9*H*-tribenzo[*a*,*c*,*e*][7]annulenes **419a–e** and they examined the photochemistry of optically active potential triggers for physical amplification of a photoresponse in liquid crystalline media (Figure 24) [210]. The optically active compounds were prepared from the reaction of **400** with chiral phosphonamides **421a**–**d** as an application of the Hanessian chiral olefination reaction. The acetyl derivative **419e** was prepared by reaction of **419c** with methyllithium. The optical purities of the compounds were determined to be 92% and 5% by NMR spectroscopy in the presence of chiral shift reagents. While UV irradiation of the benzylidene-9*H*-tribenzo[*a*,*c*,*e*][7]annulenes resulted in high-efficiency photoracemization, thermal racemization was not observed at temperatures below 100 °C.

**Figure 25:**
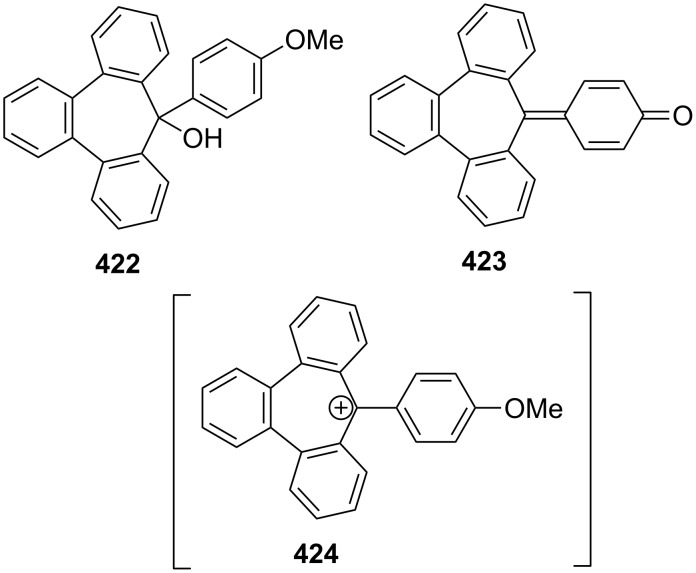
Structures of tetracyclic alcohol **422**, *p*-quinone methide **423** and cation **424**.

**Figure 26:**
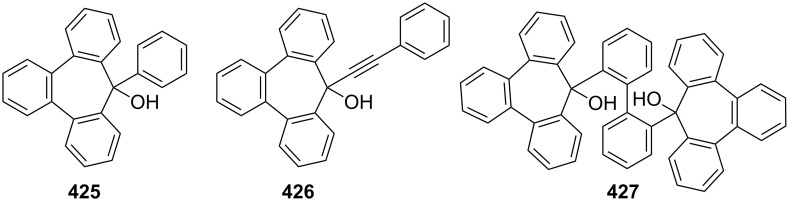
Structures of host molecules **425**–**427**.

Although the Grignard reaction between tribenzotropone (**400**) and 4-methoxyphenylmagnesium bromide provided the alcohol **422** in good yield (72%), O-dealkylation of the tetracyclic alcohol **422** gave the corresponding *p*-quinone methide **423** in low yield (23%, Figure 25) [211]. This result was attributed to the relatively low stability of the formed cation **424** due to the aromatic system twisted out of plane. Tribenzotropone (**400**) was also used as starting material for host molecules **425**–**427** (Figure 26) [212–213].

**Scheme 78:**
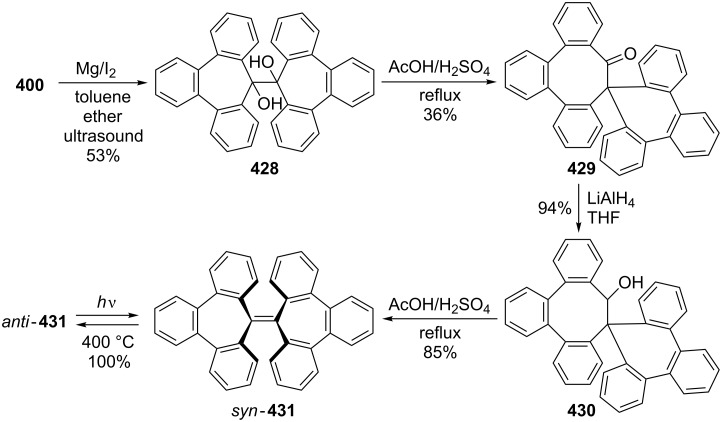
Synthesis of non-helical overcrowded derivatives *syn*/*anti***-431**.

**Figure 27:**
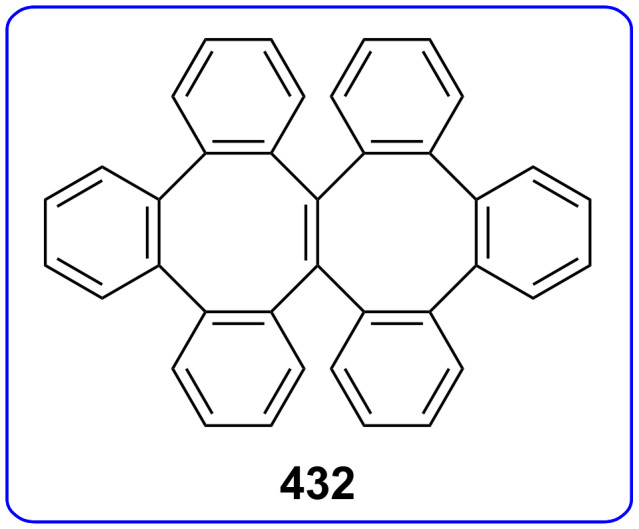
Hexabenzooctalene **432**.

As outlined in Scheme 78 with regard to the synthesis of a series of non*-*helical overcrowded derivatives, *syn*-**431** was also prepared using **400** in four steps, which covered pinacol coupling and then pinacol rearrangement, carbonyl reduction, and Wagner–Meerwein rearrangement occured [214]. Isomers *syn*-**431** and *anti*-**431** were converted as quantitative to each other at thermal and photochemical conditions as shown in Scheme 78. At the same time, the unambiguous characterization of *syn*-**431** and *anti*-**431** revealed that the previously claimed synthesis of hexabenzooctalene **432** by Tochtermann [215] was incorrect (Figure 27).

### 9. Naphthotropones

**Figure 28:**
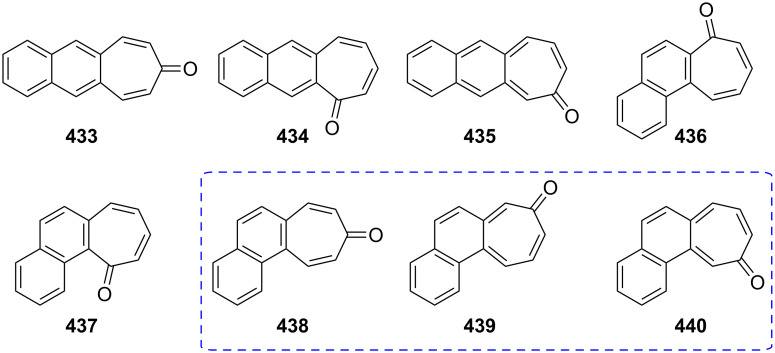
Structures of possible eight isomers **433**–**440** of naphthotropone.

Although eight isomers **433**–**440** for naphthotropone, which possess an X*H*-cyclohepta[*y*]naphthalen-Z-one (X = Y = 7, 8, 9 or 10; y = a, b) skeleton system, are possible, only five isomers **433**–**437** were found experimentally (Figure 28). Sudoh’s group reported the annulation effects of benzene rings to tropone (**1**) on the ground-state dipole moment, which can be useful for the study of molecular interactions in solution and excited states, as both the experimental and computational for the first time [216]. The ground-state dipole moments of a series of annulated tropones were computationally calculated using the Hartree–Fock (HF), density functional theory (DFT), and Møller–Plesset second-order perturbation (MP2) methods. While the ground-state dipole moment for 4,5-naphthotropone (**433**) was experimentally determined as 5.19 D, the MP2 method gave the result corresponding best to the experimental one for **433** among the three methods. The electronic transitions observed in tropone and tropolone derivatives condensed with benzene and naphthalene were studied experimentally and theoretically [217–219]. Ohkita’s group characterized the aromaticity of π-extended *o*-quinoidal tropone derivatives **433**–**435** along with five other tropone derivatives via the nucleus-independent chemical shifts (NICS), which is a computational method proven to be the most reliable probe of aromaticity due to its simplicity and efficiency [220–221]. Interestingly, the NICS(1) value calculated for the tropone ring in **433** is negative (−7.4), and indicates significantly increased aromatic character relative to the parent system. Moreover, NICS calculations demonstrated that the annulation of a benzene or naphthalene ring to the 2,3- or 4,5-position of tropone resulted in diminution of aromaticity. Furthermore, the elongations of the calculated C=O bond in the studied molecules as **433** were attributed to substantial contributions of polar resonance structures to these molecules.

#### 9.1. Synthesis and characterization studies of naphthotropones

**Scheme 79:**
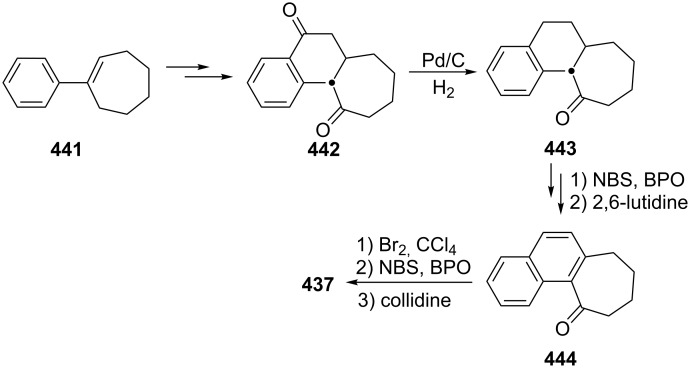
Synthesis of naphthotropone **437** starting from 1-phenylcycloheptene (**441**).

**Scheme 80:**
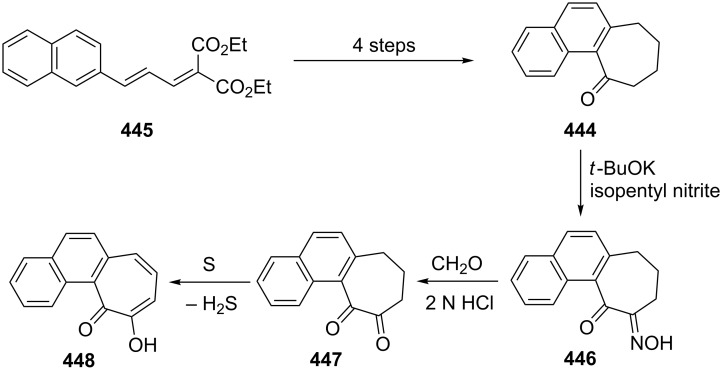
Synthesis of 10-hydroxy-11*H*-cyclohepta[*a*]naphthalen-11-one (**448**) from diester **445**.

Elad and Ginsburg reported the synthesis of a naphthotropone isomer for the first time (Scheme 79) [222]. Catalytic reduction of the key diketone **442**, which was prepared by multi-stage synthesis of 1-phenylcycloheptene (**441**), removed the carbonyl group conjugated to the benzene ring and stepwise bromination and dehydrobromination of ketone **443** afforded the desired 11*H*-cyclohepta[*a*]naphthalen-11-one (**437**) [222–223]. Treibs and Herdmann [224] reported the synthesis of 10-hydroxy-11*H*-cyclohepta[*a*]naphthalen-11-one (**448**) in very low yield starting from 2-naphthaldehyde and diethyl 2-ethylidenemalonate as outlined in Scheme 80 [224]. The condensation product **445** was converted to the ketone **444** in four steps: hydrolysis, catalytic hydrogenation, decarboxylation, and Friedel–Crafts acylation. After hydrolysis of oxime **446** derived from ketone **444**, diketone **447** was subjected to an oxidation reaction with elemental sulfur, Pd/C, or SeO_2_ to give naphthotropolene **448** in very low yield.

**Scheme 81:**
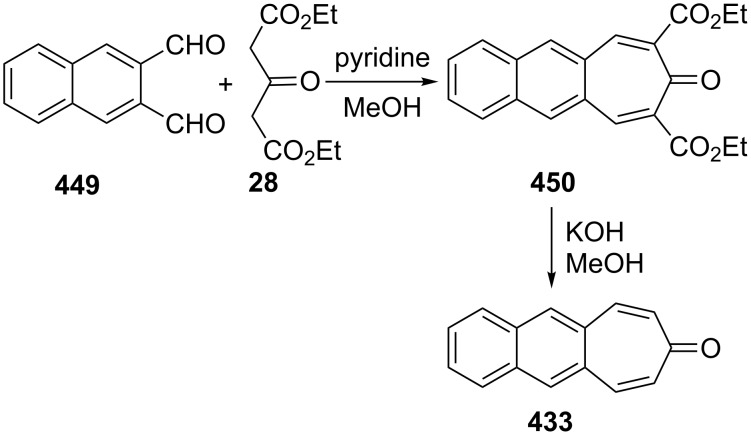
Synthesis of naphthotropone **433**.

**Scheme 82:**
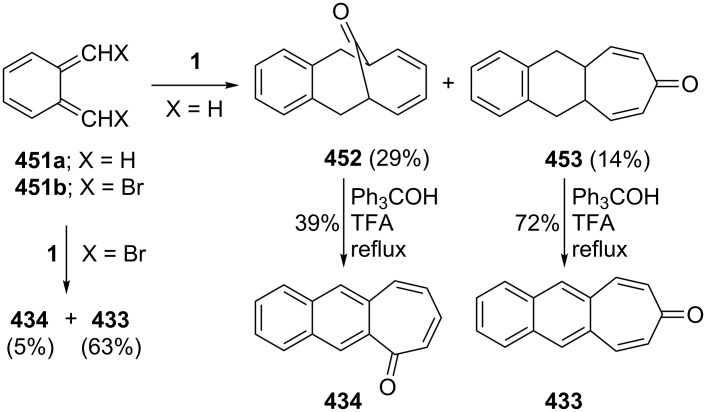
Synthesis of naphthotropones **433** and **434** via cycloaddition reaction.

**Scheme 83:**
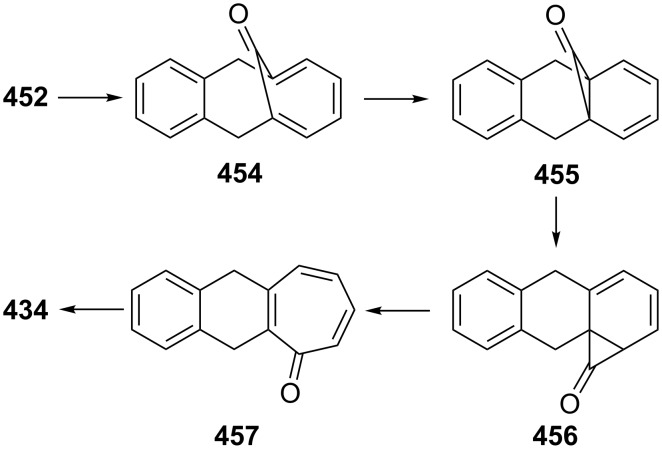
Synthesis of naphthotropone **434** starting from **452**.

**Figure 29:**
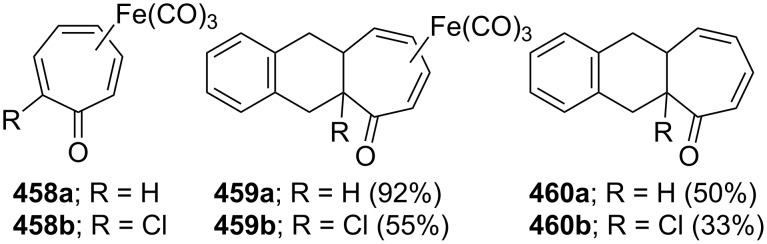
Structures of tricarbonyl(tropone)irons **458**, and possible cycloadducts **459**.

Naville’s group completed the series of benzologue tropylium cations up to C15 by preparing some tropylium cations [225]. This context with synthesis of 8*H*-cyclohepta[*b*]naphthalen-8-one (**433**) was reported featuring condensation of 2,3-naphthalenedicarboxaldehyde (**449**) with diethyl 1,3-acetonedicarboxylate (**28**) followed by decarboxylative hydrolysis of diester **450** (Scheme 81). Ito’s group reported the simple synthesis of two naphthotropone isomers utilizing the cycloaddition of tropone (**1**) (Scheme 82) [226]. Cycloaddition of exocyclic diene **451a**, obtained from *o*-xylylene **154** with excess **1** in DMF resulted in the formation of the [6 + 4] adduct **452** and [4 + 2] adduct **453**. After separation by silica gel chromatography, both of the cycloadducts were independently subjected to dehydrogenation with triphenylcarbinol in trifluoroacetic acid under reflux to yield naphthotropones **433** and **434** as the sole product in each respective reaction. Multistep synthesis of these naphthotropones was also performed through the reaction of dibromo-*o*-xylylene **451b** generated in situ from 1,2-bis(dibromomethyl)benzene at 80 °C with **1** (Scheme 82). The proposed mechanism for the transformation of **452** to **434** includes dehydrogenation, disrotatory electrocyclic ring-closing, thermal [1,5]-sigmatropic rearrangement, and again dehydrogenation steps as depicted in Scheme 83. Kanematsu’s group reported a selective reaction at the 2,3-position of tricarbonyl(tropone)iron **458a** with *o*-quinodimethane **451a** using the masking effect of tricarbonyliron complex to yield exclusively [4 + 2] adduct **459a** with no formation of other cycloadducts (Figure 29) [227]. The reaction of **459a** with *o*-chloranil in refluxing methylene chloride to remove the tricarbonyliron moiety afforded the previously unobtainable product **460a**, whereas treatment of **459b** with trimethylamine oxide provided naphthotropone **434** in 15% yield along with **460a** and its isomers. While a similar reaction of tricarbonyl(2-chlorotropone)iron **458b** and **451a** yielded the sole product **459b** (55%), 2-chlorotropone reacted poorly with **451a** to afford naphthotropone **433** (13%) as the only isolable product via [4 + 2] cycloaddition reaction followed probably by dehydrobromination and aromatization.

**Scheme 84:**
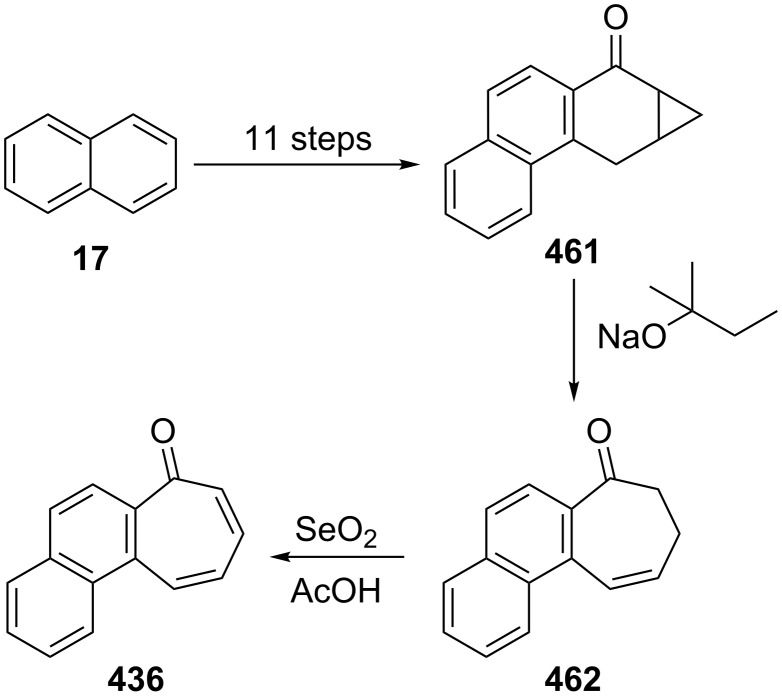
Synthesis of naphthotropone **436**.

**Scheme 85:**
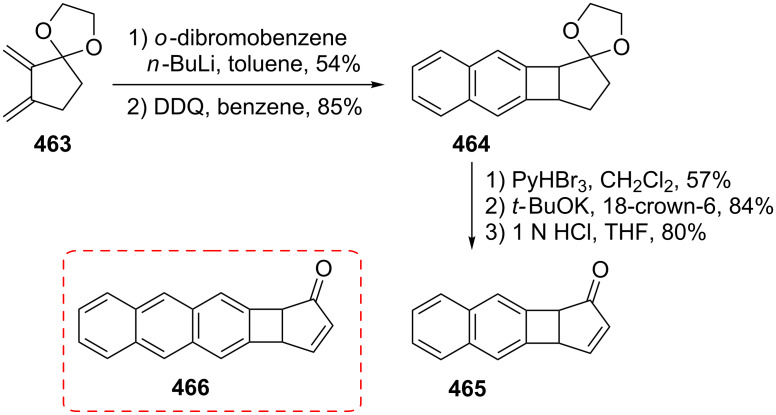
Synthesis of precursor **465** for naphthotropone **435**.

**Scheme 86:**
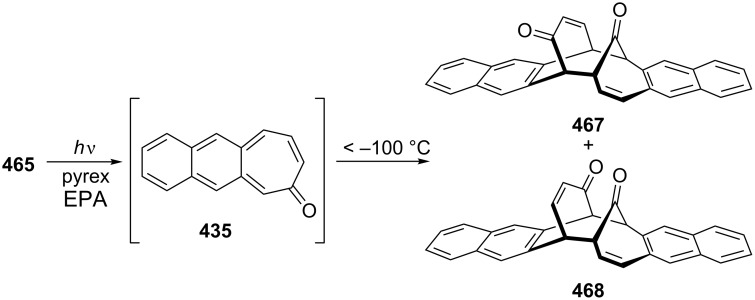
Generation of naphthotropone **435** from **465**.

Jones’ group prepared naphthotropone **436** using published procedures and known intermediates (Scheme 84) [228–231]. The ketone **461** prepared in 11 steps starting from naphthalene (**17**) was converted to **462** through ring-opening of cyclopropane with a base followed by oxidation. After previous successful generation of **13** from the corresponding benzocyclobutene **230**, Ohkita’s group also reported the synthesis of **465** as a precursor for naphthotropone **435** (Scheme 85) [220–221]. Photo-promoted [2 + 2] cycloaddition of 2-cyclopentenone with (*E*)-1,4-dichloro-2-butene followed by protection of the carbonyl group and subsequent dehydrohalogenation afforded diene **463**, which was converted to **465** after a series of reactions including the Diels–Alder reaction with benzyne, dehydrogenation with DDQ, bromination, dehydrobromination, and acid-catalyzed hydrolysis of the ketal group. Irradiation of **465** in a rigid class at −196 °C resulted in the formation of the hitherto unknown 7*H*-cyclohepta[*b*]naphthalen-7-one (**435**), which displayed characteristic UV–vis absorption extending to 700 nm and underwent rapid dimerization to give the dimers **467** and **468** (Scheme 86). However, Okhita’s group applied this strategy to generate the corresponding anthracene-tropone from **466** under the same reaction conditions (Scheme 85). However, anthro-cyclobutene derivative **466** failed to result in ring-opening for the expected tropone and the starting material **466** was recovered quantitatively. The products were unambiguously characterized as *syn*-[π12 + π14] dimers **467** and **468** by X-ray crystallography, and the preferential *syn*-dimerization was attributed to the extended secondary orbital interactions. Sato’s group also reported the IR spectra of **435** generated in nitrogen matrices at 13 K by monochromic irradiation with a XeCl excimer laser to investigate medium effects on the molecular structures of tropones [156].

#### 9.2. Applications of naphthotropones

**Figure 30:**
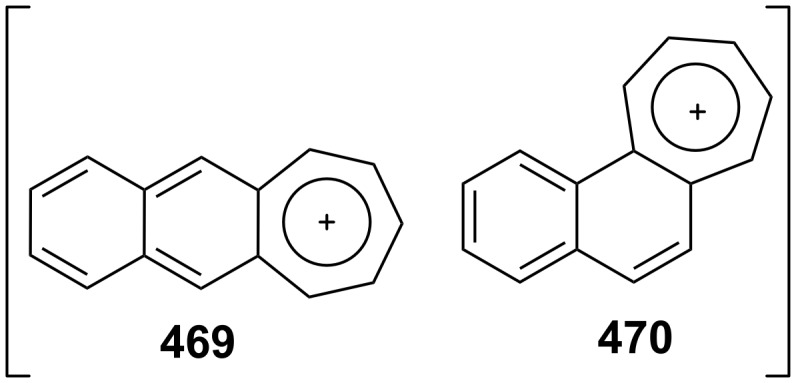
Structures of tropylium cations **469** and **470**.

In connection with the completion of the benzologue tropylium series, Naville’s group also prepared the tropylium cations **469** and **470** from the corresponding naphthotropones **433** and **436** and described the absorption spectra and the relative acidities of all cations (Figure 30) [225]. After hydride reduction of tropones, the alcohols in sulfuric acid provided the corresponding cations.

**Figure 31:**
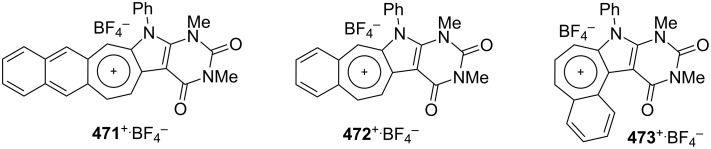
Structures of tropylium ions **471^+.^**BF_4_^−^, **472^+.^**BF_4_^−^, and **473^+.^**BF_4_^−^.

**Scheme 87:**
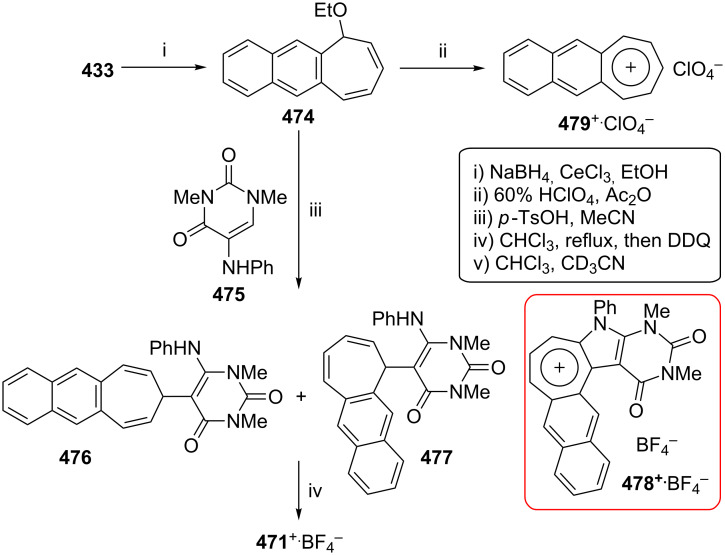
Synthesis of tropylium ions **471^+.^**BF_4_^−^ and **479^+.^**ClO_4_^−^.

Due to encouraging initial results obtained regarding the synthesis, properties, and reactivity of catacondensed aromatic π-systems as well as their photoinduced autorecycling oxidizing reactions toward some alcohol and amines [66–71], Nitta’s group focused on novel tropylium ions **471^+.^**BF_4_^−^, **472^+.^**BF_4_^−^, and **473^+.^**BF_4_^−^ containing heterocyclic moieties (Figure 31) [232]. The synthesis of **471^+.^**BF_4_^−^ was achieved by three-step reactions in modest yield (19%) starting from naphthotropone **433** while generation of **479^+.^**ClO_4_^−^ was not observed (Scheme 87). The napthotropylium cation **479^+.^**ClO_4_^−^ was prepared in 64% yield by the reduction of **433** with NaBH_4_ in EtOH in the presence of CeCl_3_ followed by subsequent treatment of **474** with 60% aqueous HClO_4_ in Ac_2_O. The synthesis of **472^+.^**BF_4_^−^ and **473^+.^**BF_4_^−^ as a mixture was carried out in similar ways starting from benzotropone **11** and its separation was performed by fractional recrystallization from MeCN/EtOAc to give pure samples. While the compounds **471^+.^**BF_4_^−^, **472^+.^**BF_4_^−^, and **473^+.^**BF_4_^−^ were fully characterized on the basis of spectroscopic methods as well as elemental analysis and X-ray analysis, their chemical shifts provided quite noteworthy information for determining structural properties such as diatropicity and bond alternation. The carbocation stability is expressed in terms of its p*K*_R+_ value, which is the affinity of the carbocation toward hydroxide ions, and this value is the most common criterion for carbocation stability. Although the p*K*_R+_ values for cations **471^+^**, **472^+^**, and **473^+^** were determined spectrophotometrically as the values of ca. 0.5–9.0, the p*K*_R+_ value of napthotropylium ion **479^+^** was clarified as much lower, at <0. Autorecycling oxidation properties of some amines as well as the reduction potentials and the reactions with some nucleophiles of the compounds **471^+.^**BF_4_^−^, **472^+.^**BF_4_^−^, and **473^+.^**BF_4_^−^ were also reported. The oxidations of benzylamine, 1-phenylethylamine, hexylamine, and cyclohexylamine with **471^+.^**BF_4_^−^, **472^+.^**BF_4_^−^, and **473^+.^**BF_4_^−^ produced the corresponding imines under aerobic and photoirradiation conditions.

**Scheme 88:**
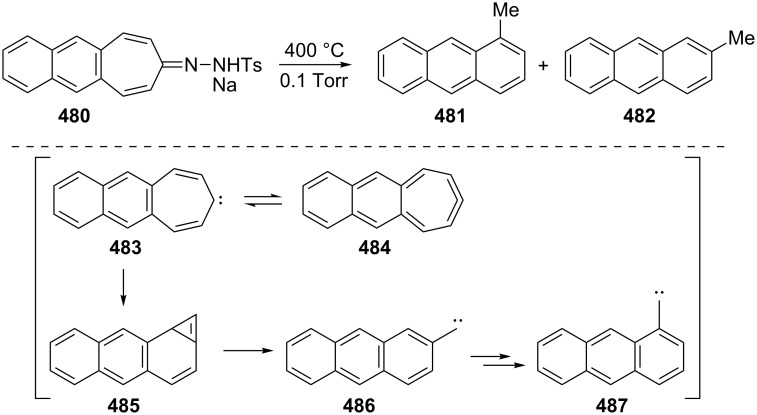
Synthesis of 1- and 2-methylanthracene (**481** and **482**) via carbene–carbene rearrangement.

**Figure 32:**
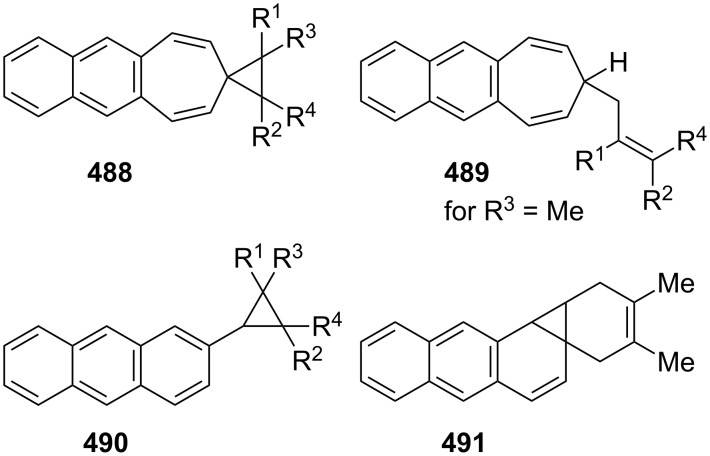
Trapping products **488**–**490**.

The napthotropones **433** and **436** were also used to prepare tosylhydrazones and their salts in the usual manner as precursors of the corresponding carbenes. Hackenberger and Dürr reported the generation and chemistry of naphtho[*b*]tropylidene **483** (Scheme 88) [233–234]. Carbene **483** generated by flash solvolysis from the salt **480** in the gas-phase led to the formation of 1- and 2-methylanthracene (**481** and **482**) via carbene–carbene rearrangement to anthrylcarbene **486** to **487** as a decisive step (Scheme 88). In the condensed phase, while the trapping of the carbene **483** with olefins yielded cycloaddition products **488** and insertion products **489**, the cycloadducts **490** through intermediate anthrylcarbene **486** also occurred as byproducts (Figure 32). However, if electron-deficient alkenes were used, the amount of cycloadduct **490** increased. The intermediate **485** was trapped by 2,3-dimethylbut-2-ene to afford **491** (Figure 32). The reactions and products described were attributed to an equilibrium mixture of singlet **483**, triplet **483**, and bicycle **485**.

**Scheme 89:**
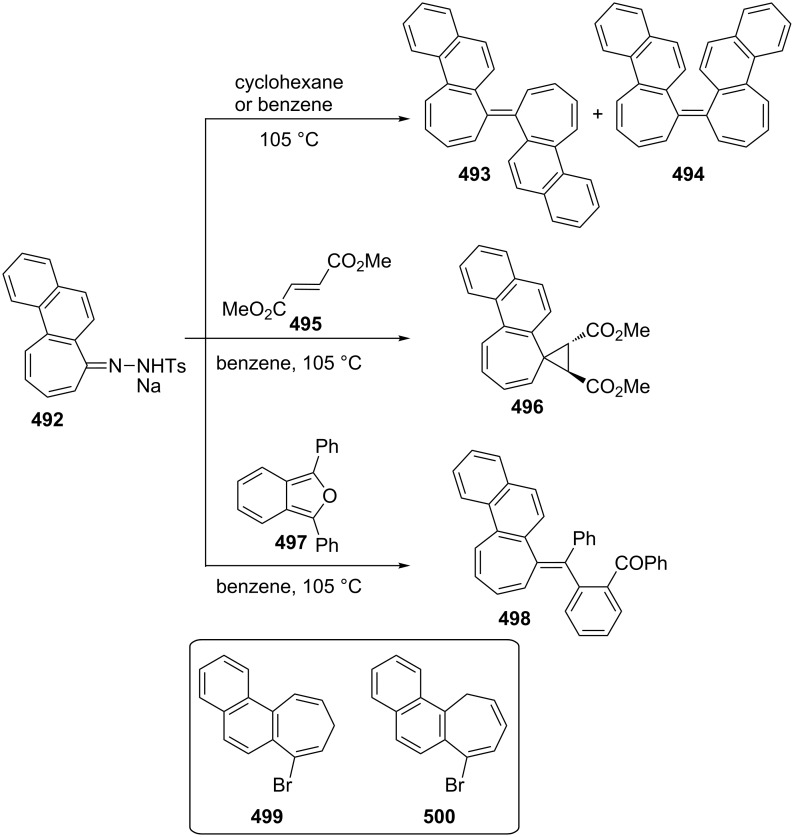
Generation and chemistry of a naphthoannelated cycloheptatrienylidene-cycloheptatetraene intermediate from salt **492**.

**Scheme 90:**
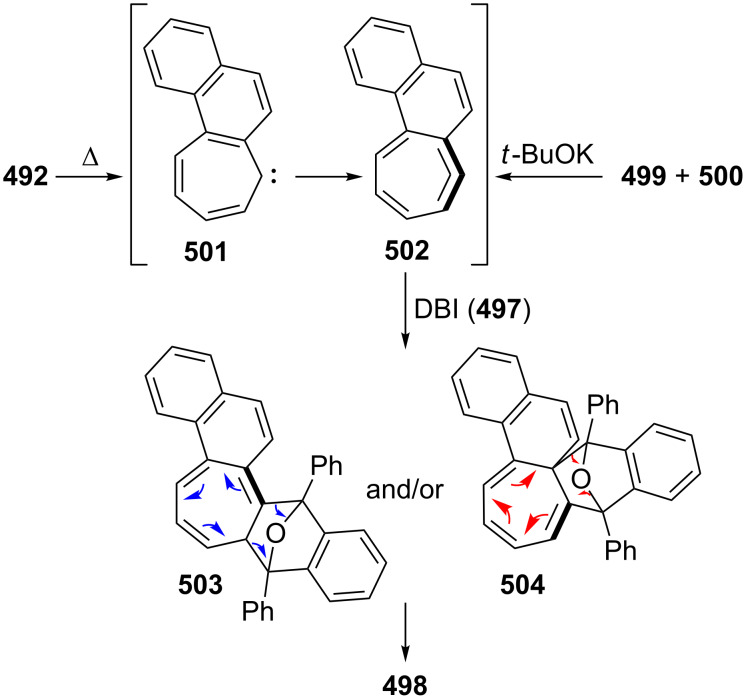
Proposed intermediates and reaction pathways for adduct **498**.

Jones’ group also reported the generation and properties of a naphthoannelated cycloheptatrienylidene-cycloheptatetraene intermediate from both the corresponding salt **492** derived from **436** or a mixture of bromocycloheptatrienes **499** and **500** (Scheme 89) [228]. While thermolysis of salt **492** in cyclohexane or benzene afforded only a mixture of naphthoannelated heptafulvalenes **493** and **494**, thermolysis in the presence of dimethyl fumarate (**495**) yielded the expected spirocyclopropane **496** along with trace amounts of the same two dimers. Thermolysis of **492** in the presence of diphenylisobenzofuran (DBI, **497**) gave a new adduct, **498**. Dehydrobromination of a mixture of bromocycloheptatrienes **499** and **500** with potassium *tert*-butoxide in the presence of **497** resulted in the formation of the rearranged adduct **498** along with carbene-dimer products **493** and **494**. Valance isomerization of carbene **501** to allene **502** plays a critical role in the proposed mechanism for the formation of the adduct **500**, which was formed by Diels–Alder addition of **497** to the allene **502** followed by rearrangement as depicted in Scheme 90. Based on INDO calculations of a number of the carbenes and allenes, Jones’ group deduced that while the chemistry of cycloheptatrienylidene and in some its annelated relatives are dominant in some cases by the allene form and in others by triplet carbene, the role of singlet carbene is uncertain.

**Scheme 91:**
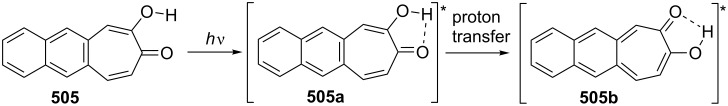
Exited-state intramolecular proton transfer of **505**.

Jang and Kelley studied the exited-state intramolecular proton transfer (ESIPT) and relaxation of 7-hydroxy-8*H*-cyclohepta[*b*]naphthalen-8-one (**505**) in room temperature solutions studied using static and time-resolved absorption as well as emission spectra for the equations indicated in Scheme 91 [235–236]. Dual fluorescence (normal and tautomer fluorescence) is observed in the protic solvent (ethanol), while only tautomer fluorescence is observed in the nonpolar solvent (cyclohexane). The dual green and red fluorescence arise from the intermolecular hydrogen-bonded normal molecules and the tautomer molecules with proton transfer in the excited state (ESIPT), respectively. The observed fluorescence lifetimes and quantum yields in ethanol and cyclohexane solutions could be attributed to competition between intersystem crossing and proton transfer in the first excited singlet state.

### 10. Miscellaneous benzotroponoids

#### 10.1. Benzoditropones

**Figure 33:**
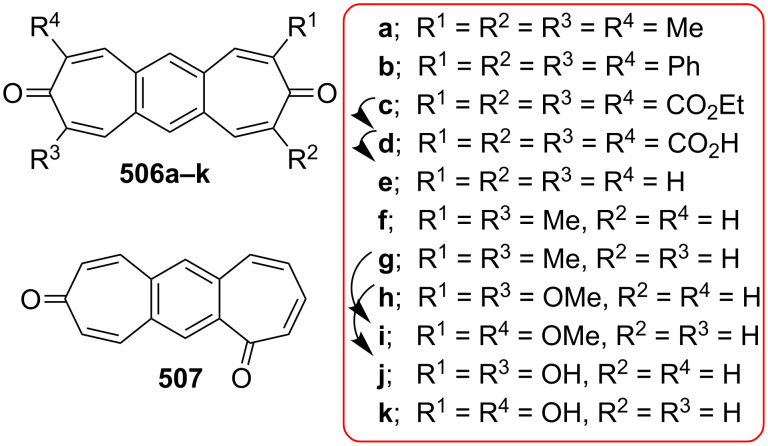
Benzoditropones **506** and **507**.

Although benzoditropone has many isomeric possibilities, only two isomers **506** and **507** of the benzoditropone system have been reported (Figure 33). The X-ray diffraction studies for benzo[1,2:4,5]di[7]annulene-3,9-dione (**506e**) as the main skeleton revealed a nearly planar geometry [237]. The intermolecular distances confirmed good agreement with normal van der Waals interactions, while the intramolecular distances led to a significant bond alternation within the seven-membered rings.

**Scheme 92:**
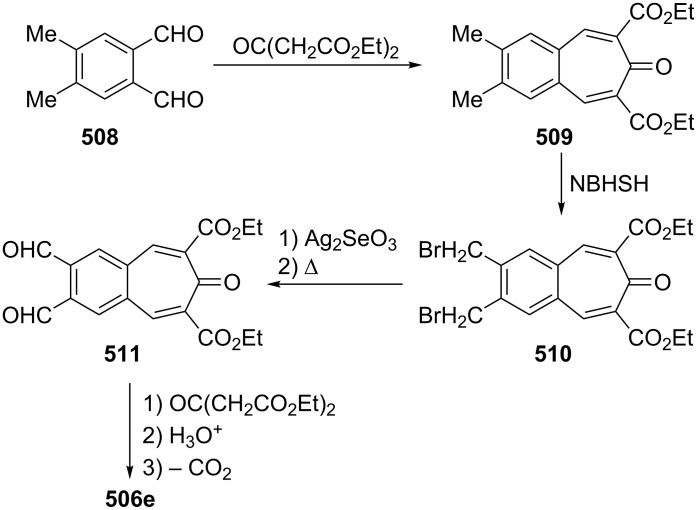
Synthesis of benzoditropone **506e**.

Aldol-type cyclizations provide an expedient access to the benzoditropones in a single step. Föhlisch and Widmann applied aldol-type cyclization for the synthesis of benzoditropones **506c–e** (Scheme 92) [238]. In an analogous manner, Soyer and Kerfento, and subsequently Soyer, attempted Aldol-type condensations of benzene-1,2,4,5-tetracarbaldehyde with the corresponding acetone derivatives to give the benzoditropone derivatives **506a–k** (Figure 33) [237–239]. An increasing bathochromic effect was observed for **506e** (R^1^–R^4^ = H) < **508c** (R^1^–R^4^ = CO_2_Et) < **506a** (R^1^–R^4^ = Me) < **506b** (R^1^–R^4^ = Ph).

**Scheme 93:**
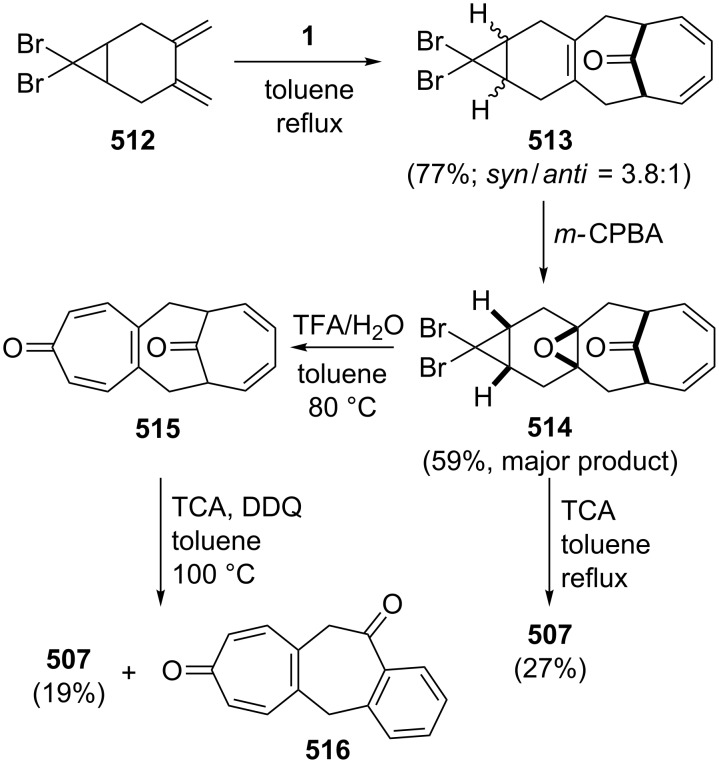
Synthetic approaches for dibenzotropone **507** via tropone (**1**).

**Scheme 94:**
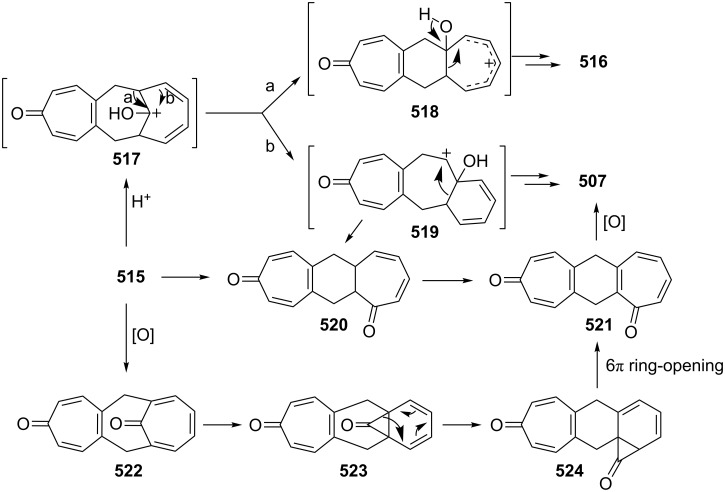
Formation mechanisms of benzoditropone **507** and **516** via **515**.

Kato’s group reported the synthesis of benzoditropones **507** via cycloaddition between tropone (**1**) and 7,7-dibromo-3,4-dimethylenebicyclo[4.1.0]heptane (**512**, Scheme 93) [240]. To this end, diene **512** reacted with **1** to give a mixture (77% yield, *syn*/*anti* = 3.8:1) of [6 + 4] cycloadducts **513** in refluxing toluene, while the reaction proceeded with high regioselectivity (90% yield, *syn*/*anti* = 9:1) in benzene in a sealed tube at 100 °C. Epoxidation of **513** with *m*-chloroperbenzoic acid (*m*CPBA) afforded a mixture containing epoxide **514** as a major product. This epoxide was then converted to benzoditropones **507** after direct or indirect steps. Bromination of **515** with molecular bromine followed by dehydrobromination by heating at 100 °C in *N,N*-dimethylformamide (DMF) also provided an improved route to **507** (56% yield). Formation of **507** and **516** from **515** occurs by the three mechanisms depicted in Scheme 94. Firstly, the protonated **517** may undergo two different 1,2-cationic rearrangements via **518** and **519** intermediates to yield **516** and **507**. Secondly, tropone-ketone **515** undergoes a thermal [1,5]-sigmatropic shift followed by successive dehydrogenation to give benzoditropone **507**. Lastly, initial dehydrogenation of **515** to bistropone **522** followed by 6π-electrocyclic ring-opening and a [1,5]-sigmatropic shift of the carbonyl carbon results in norcaradienone **524**, which undergoes 6π-retrocyclization followed by oxidation to give **507**.

**Scheme 95:**
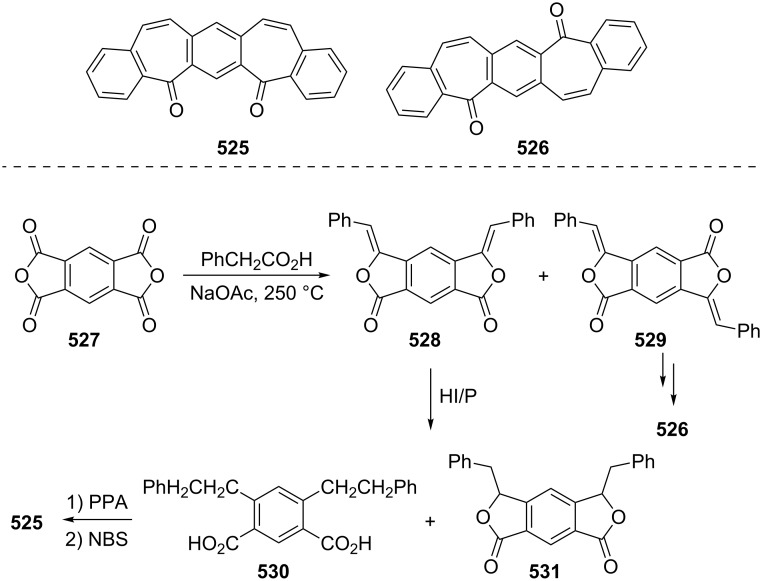
Synthesis of benzoditropones **525** and **526** from pyromellitic dianhydride (**527**).

Agranat and Avnir reported the synthesis of the benzoditropone systems **525** and **526**, which may be considered double dibenzotropones (Scheme 95) [241]. Double Perkin condensation between pyromellitic dianhydride (**527**) and phenylacetic acid gave a mixture of the two isomeric lactones, **528** and **529**, in the ratio of 5:3, which were separable by repeated fractional crystallization. The reduction of **528** and **529** with red phosphorus in boiling hydroiodic acid led to the formation of isophthalic acid derivatives (such as **530**), which underwent intramolecular Friedel–Crafts acylation by polyphosphoric acid (PPA) to construct a seven-membered ring. The synthesis of benzoditropones **525** and **526** involved the dehydrogenation of the corresponding Friedel–Crafts products with *N*-bromosuccinimide (NBS) in the presence of benzoyl peroxide followed by treatment with trimethylamine.

#### 10.2. Benzocyclobutatropones

**Figure 34:**
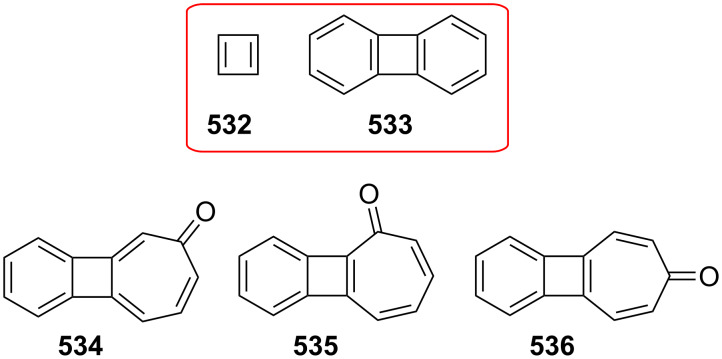
Possible three benzocyclobutatropones **534**–**536**.

Cyclobutadiene (**532**), the smallest annulene, is an unstable hydrocarbon with an extremely short lifetime in the free state and has attracted much attention from both experimental and theoretical viewpoints (Figure 34). Although **532** rapidly dimerizes via a Diels–Alder reaction, its dibenzo-derivative **533** (biphenylene) is thermally stable and shows many of the properties associated with aromatic compounds (Figure 33) [242–246]. Three possible isomeric benzocyclobutatropones, **534**–**536**, which are analogues of biphenylene in which one benzenoid ring has been replaced by the tropone ring, are of significant interest due to the question of the extent of π-electron delocalization in the seven-membered ring (Figure 34). Benzocyclobutatropones **535** and **534**, which possess a formal benzocylobutadienoid double bond, are also of particular interest.

**Scheme 96:**
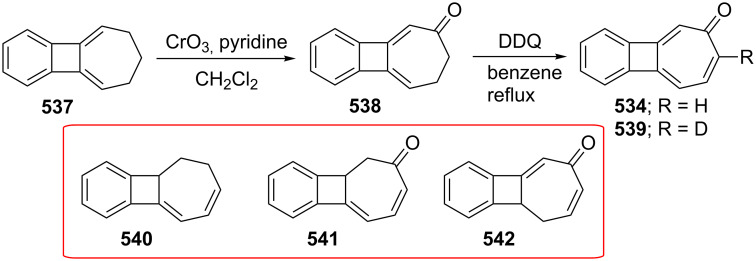
Synthesis of benzocyclobutatropones **534** and **539**.

Wege’s group attempted to prepare the main analogues **534**–**536** of a benzocyclobutatropone system [247–249]. Allylic oxidation of diene **537** with chromium trioxide–pyridine complex in dichloromethane occurred to afford dienone **538** in 21% yield, which was exposed to DDQ in refluxing benzene to give **534** in low yield (9–10%) as a stable and crystalline solid at room temperature along with some of the starting material **537** (Scheme 96) [247–248]. Deuterated derivative **539** was prepared to confirm structural assignments. NMR results showed that the seven-membered ring of **534** has a more localized π-bond system than tropone itself [248]. The CrO_3_-oxidation product **542** of the benzyne-cycloheptatriene adduct **540** was also converted to **534** after a sequence of NBS-bromination and dehydrobromination with DBU. However, the major oxidation product **542** did not react with DDQ in refluxing benzene.

**Scheme 97:**
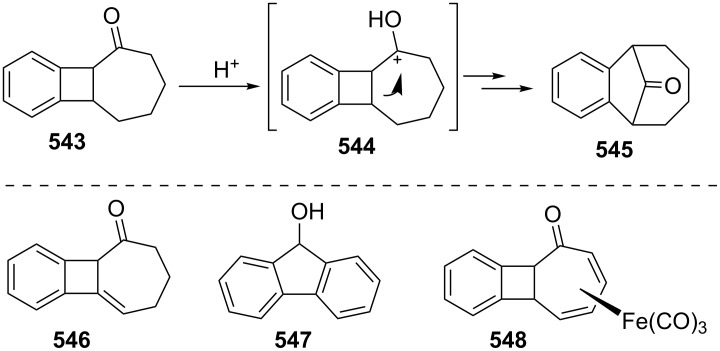
Synthesis attempts for benzocyclobutatropone **545**.

All attempts to prepare the other benzocyclobutatropone, **545**, have failed so far (Scheme 97). The potential precursor **543** of **545** was verified to be extremely acid-sensitive, and ketone **543** was rearranged to afford the bridged ketone **545** in high yield via cationic intermediates [247–248]. Another attempt then aimed to introduce a second double bond into the seven-membered ring of ketone **546**, which reacted with *N*-bromosuccinimide followed by treatment with tetrabutylammonium bromide to yield fluoren-9-ol (**547**) as the only isolable product [248]. After unsuccessful attempts resulting from the propensity of reaction intermediates to undergo skeletal rearrangements, Wege’s group attempted the preparation of ketone **548**, in which π-electrons binding to the iron carbonyl moiety as the driving force for isomerization should be suppressed [248]. However, attempts towards the preparation of the complex **548** were not successful.

**Scheme 98:**
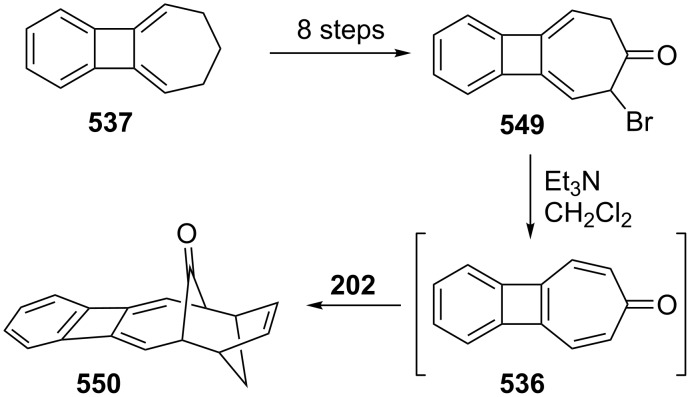
Generation and trapping of symmetric benzocyclobutatropone **536**.

To access the symmetrical tropone derivative **536**, the cycloadduct **537** was again used as a starting material since this compound contains the necessary ring skeleton of **536** and possesses the diene function permitting the introduction of the essential carbonyl group (Scheme 98) [248–249]. Compound **537** was transformed to monobromo **549** in 8 steps, which reacted with trimethylamine in the presence of cyclopentadiene (**202**) in dichloromethane at 0 °C to give the trapping product **550** in 20% yield as [6 + 4] cycloadduct. This result was attributed to the formation of benzocyclobutatropone **536**. The reaction performed without **202** gave no recognizable product.

**Scheme 99:**
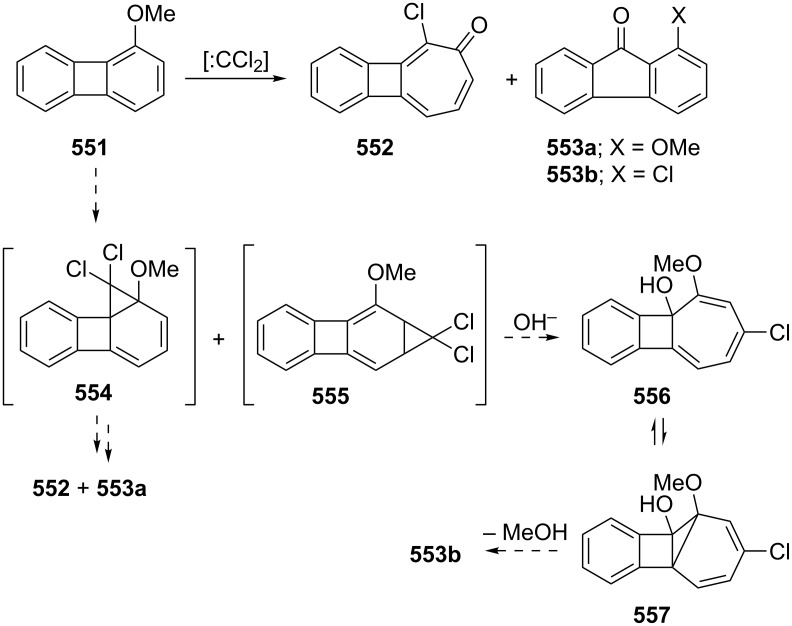
Synthesis of chloro-benzocyclobutatropone **552** and proposed mechanism of fluorenone derivatives.

**Scheme 100:**
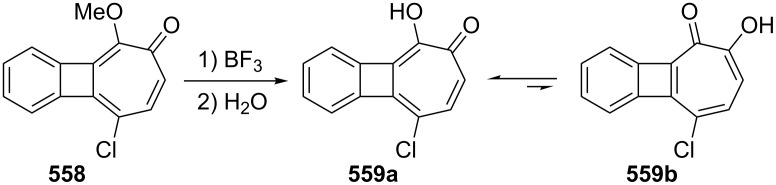
Synthesis of tropolone analogue **559**.

Ebine’s group were the first to report the addition reaction between 1-methoxybiphenylene (**551**) and dichlorocarbene generated from chloroform to give chloro-benzocyclobutatropone **552** (1.7%) together with two fluorenone derivatives, **553a** (0.8%) and **553b** (1.3%), in very low yields (Scheme 99) [250]. The formation mechanism for the products is also provided as depicted in Scheme 99. Moreover, Ebine’s group investigated the reaction of 1,2-dimethoxybiphenylene with dichlorocarbene to detect the bond fixation in biphenylene derivatives [251]. While the reaction gave two chloro-methoxy-benzocyclobutatropones and four fluorenones with chloro and methoxy substituents, similar products were obtained with dibromocarbene. The formation of these products was attributed to unequivocal chemical evidence for bond fixation of 1,2-dimethoxybiphenylene. Furthermore, cleavage of the ether functionality with boron tribromide in dichloromethane at −65 °C provided the first example of tropolone analogue **559a** (93%) of biphenylene (Scheme 100). Electronic spectra and NMR coupling constants of the compound showed that **559a** exists as only one tautomer due to instability of the antiaromatic cyclobutadiene structure in the central four-membered ring of **559b**.

**Scheme 101:**
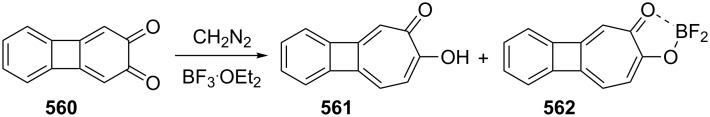
Synthesis of tropolones **561** and **562**.

At the same time, Ebine’s group reported the reaction of biphenylene-2,3-quinone (**560**) with diazomethane in the presence of boron trifluoride etherate to give another tropolone analogue **561** and its boron difluoride chelate **562**, which was hydrolyzed in acidic aqueous ethanol to **561** quantitatively (Scheme 101) [252–253]. On the other hand, some electrophilic reactions, including nitration, bromination, and azo coupling for **561** yielded only 7-substituted tropolones.

#### 10.3. Benzotropoquinones

**Figure 35:**
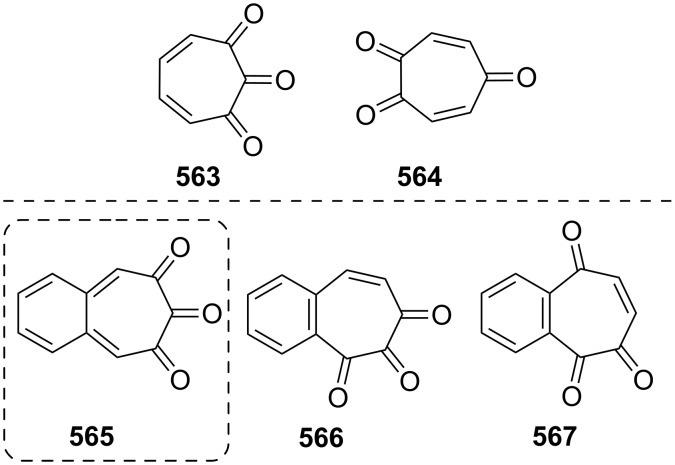
*o*/*p*-Tropoquinone rings (**563** and **564**) and benzotropoquinones (**565**–**567**).

**Scheme 102:**
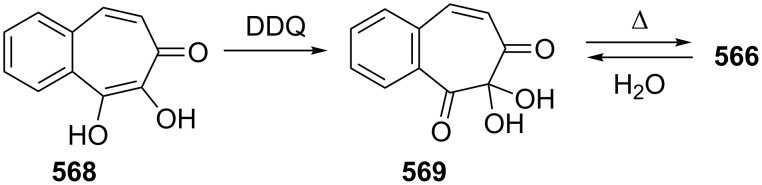
Synthesis of benzotropoquinone **566**.

**Scheme 103:**
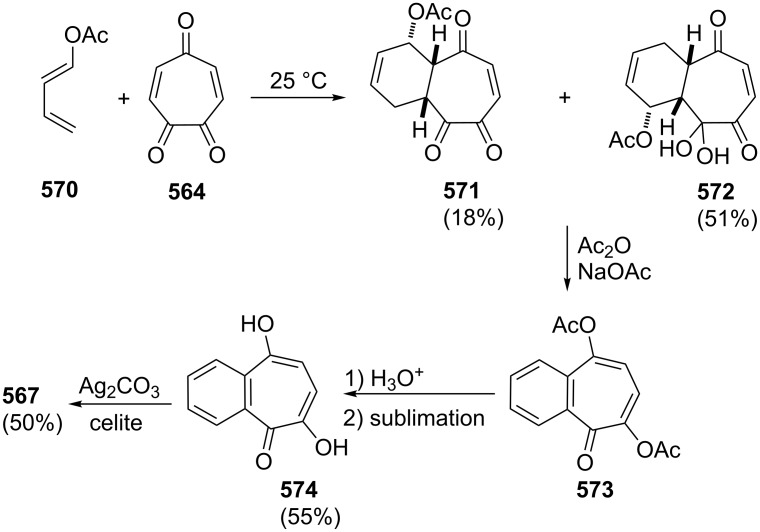
Synthesis of benzotropoquinone **567** via a Diels–Alder reaction.

Benzene annulations to *o*- and *p*-tropoquinone rings (**563** and **564**) have also attracted interest due to their unique quinone characteristics as in benzoquinone series of *o*- and *p*-tropoquinones (Figure 35). Both **566** and **567** of possible benzo analogues containing tropoquinone rings were synthesized, and their properties were described (Figure 34) [254–255]. Oxidation of hydroxybenzotropone **568** with DDQ in acetone at room temperature followed by addition of water provided 1,2,3-benzotropoquinone hydrate **569** in 85% yield, which was carefully sublimated to afford the desired 1,2,3-benzotropoquinone **566** in low yield (18%, Scheme 102) [254]. The synthesis of starting **568** was reported by Hartwig’s group [197]. Benzotropoquinone **566** is gradually decomposed in dry air and is highly hygroscopic, giving **569**. 1,2,5-Benzotropoquinone **567** was prepared by starting from the Diels–Alder reaction between 1-acetoxy-1,3-butadiene (**570**) and *p*-tropoquinone (**564**) in a four-step synthesis (Scheme 103) [255]. The acetylation of cycloadduct **571** or **572** provided the diacetoxybenzotropone **573**, which was converted to benzotropoquinone **567** after acid hydrolysis and oxidation steps.

**Figure 36:**
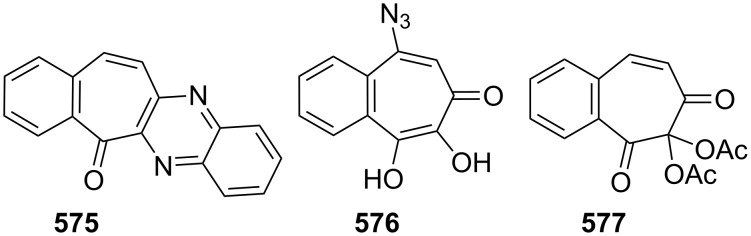
Products **575–577** through 1,2,3-benzotropoquinone hydrate **569**.

Due to the highly hygroscopic nature of **566**, chemical reactions of hydrated **569** were studied [254]. The reaction of **569** with *o*-phenylenediamine at room temperature afforded the quinoxaline derivative **575** (15%) along with benzo[*a*]phenazine (13%, Figure 36). While the reaction between NaN_3_ and **569** gave **576** through conjugate addition followed by dehydration (Figure 36), treatment of **569** with concentrated HCl at room temperature provided **568** in 80% yield. Furthermore, the corresponding diacetate **577** was obtained in 87% yield from the acetylation of **569** in the presence of H_2_SO_4_ (Figure 36). Acetylation of **569** with BF_3_ catalyst resulted in the formation of 1,2-diacetoxy-naphthalene (25%) and 3,3’,4,4’-tetraacetoxy-1,1’-binaphthyl (15%) together with **577** (15%).

**Scheme 104:**
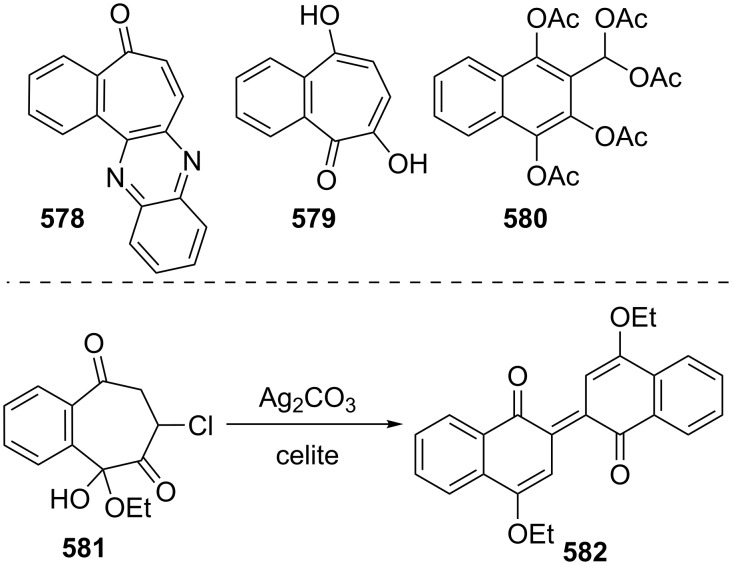
Structures **578–582** prepared from tropoquinone **567**.

Although 1,2,5-benzotropoquinone **567** is highly sensitive to moisture, it is stable under anhydrous conditions in the dark and, its hygroscopic form returns to **567** when dried under a vacuum. While the reaction of tropoquinone **567** with *o*-phenylenediamine gives a quinoxaline derivative **578**, the reduction of **567** to **579** was realized via catalytic hydrogenation with Pd/C (Scheme 104) [252]. A naphthaldehyde derivative **580** was derived from Thiele acetylation (Ac_2_O, H_2_SO_4_, room temperature) of **567** in 11% yield. Treatment of tropoquinone **567** with hydrogen chloride in ethanol gave the adduct **581** (74% yield), which was oxidized with silver carbonate-celite to yield the indigoid **582** (30%). Upon the addition of methanol, **567** reversibly forms a mixture of the corresponding methyl acetals through adjacent diketone.

#### 10.4. Dibenzotropoquinones

**Figure 37:**
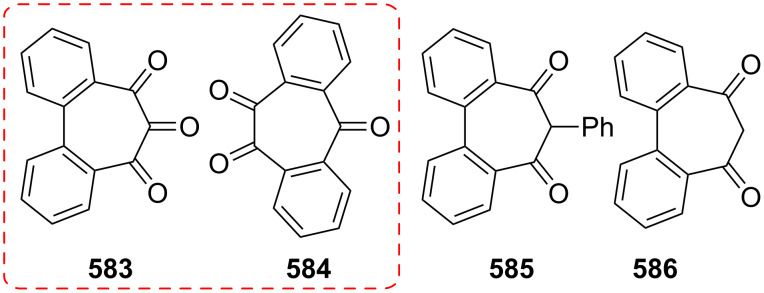
Two possible structures **583** and **584** for dibenzotropoquinone, and precursor compound **585** for **583**.

Two possible structures for dibenzotropoquinone, 5*H*-dibenzo[*a,c*][7]annulene-5,6,7-trione (**583**) and 5*H*-dibenzo[*a,d*][7]annulene-5,10,11-trione (**584**), are already known (Figure 37). Firstly, triketone **583** was prepared by oxidation of the activated methylene group of diketone **586** with selenium dioxide [256–257]. Oxidative degradation of diketone **585** with nitric acid also provided **583** (Figure 36) [258–259]. The synthesis of dibenzotropoquinone **584** was realized via SeO_2_-mediated oxidation of 5*H*-dibenzo[*a,d*][7]annulen-5-one (**399,** Figure 19) [260–261].

**Scheme 105:**
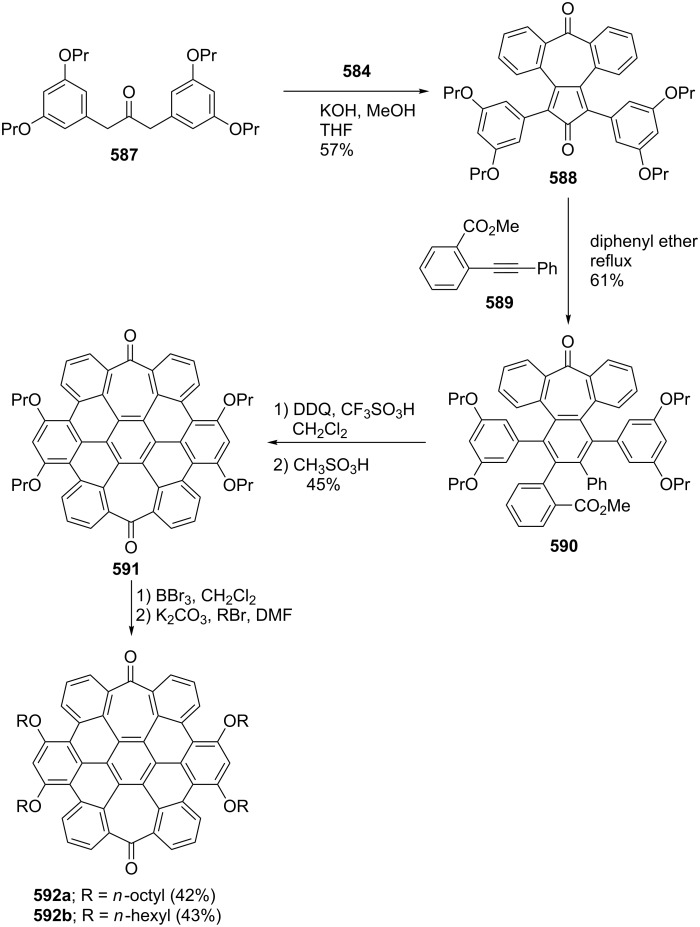
Synthesis of saddle-shaped ketone **592** using dibenzotropoquinone **584**.

Conjugated carbon nanomaterials such as fullerenes, carbon nanotubes, and graphene have received tremendous attention and have great potential application in nanoscience due to their exceptional electrical, thermal, chemical, and mechanical properties. Starting from dibenzotropoquinone **584**, Miao’s group reported the synthesis of saddle-shaped ketone **592** containing two tropone subunits embedded in the well-known framework of *peri*-hexabenzocoronene as depicted in Scheme 105 [261]. However, bistropone **592** was used as a precursor for the successful synthesis of two novel large aromatic saddles (C_70_H_26_ and C_70_H_30_) by reactions on the carbonyl groups. Local aromaticity and nonplanarity of individual rings in these saddle-shaped π-backbones were confirmed by crystal structure analysis. Moreover, preliminary studies on semiconductor properties were performed.

## Conclusion

Tropones and tropolones are an important class of seven-membered aromatic compounds. In addition, hundreds of tropone or tropolone derivatives are known in the literature. These kinds of products have a wide range of biological activity and are building blocks in the synthesis of many molecules. All these factors have made these molecules a focus of intense interest among both organic chemists and medical chemists for nearly a century. This chemistry is one of the milestones leading to a deeper understanding of static, dynamic, and multidisciplinary aspects of organic chemistry such as spectroscopic studies, mechanistic and synthetic investigations, theoretical calculations, aromaticity, evolution, and design of bioactive molecules and molecular materials.

In this review, we have described the numerous efforts concerning synthesis and applications in benzotropone chemistry spanning over 100 years, from the first works up to the most recent. The review covers isomeric benzotropones and tribenzotropones as well as their benzotropolone analogues. As it is well known, halogenated compounds are very valuable as they are the key compounds for many functionalizations. Therefore, halogenated benzotropones and benzotropolones are also included in this review. Tropoquinones are a topic of interest in organic research and these compounds are used for many functionalization reactions. Works on benzo analogues of tropoquinones are also summarized in this review. Carbene–carbene and carbene–allene rearrangements on benzo[7]annulene ring-derived benzotropones are investigated in detail in the literature and discussed in this review. Carbene insertion reaction, synthesis of azocine, synthesis and physical properties of homo- and bis-homobenzotropones, and their conversation to corresponding homotropolium cations are also other well-investigated issues reviewed in this work. Knowledge of the chemistry of benzocyclobutenotropones, naphthotropones, and their tropolone analogues is limited and more research on those compounds is required in the future.

Numerous synthetic efforts towards the synthesis and chemical reactivity of benzotropones and benzotropolones were reported from the 20th century to date. In addition to being natural products, many benzotropone derivatives can be prepared directly by oxidation of seven-membered rings. They can also be derived from cyclization, ring expansion, or cycloaddition of appropriate precursors followed by elimination or rearrangement. The oxidation of seven-membered rings generally gives a mixture, whereas cyclization of suitable acylic compounds or ring expansion reactions generally produces one isomer in high yield. Although 2,3- and 4,5-benzotropone have been investigated in detail, research on 3,4-benzotropone is rather limited due to instability of this kind of compound, which is attributed to the *o*-quinoidal structure, and because it does not have a sextet electron system in the benzene ring.

In general, two kinds of reactions on benzotropone and their analogues are common: i) reaction on the carbonyl group, which is generally a nucleophilic addition or condensation, ii) reaction on the double bond in the seven-membered ring, which is generally with a nucleophile since the tropone ring is behaving as an electrophile. The double bonds in the seven-membered ring give a cycloaddition reaction as both a diene and a dienophile. Although many reactions on this hydrocarbon have been reported, we think that there is still a need for the scientific community to develop many synthetic methods and investigate their possible interesting synthetic applications in various fields. We consider the objectives of this review as helping in the systematization of the literature data collected to date and allowing a better understanding of them, and possibly bringing new ideas to the field. We strongly believe that the synthetic potential and applications of this chemistry have not yet been fully revealed, and there are certainly further challenges and opportunities for reinvestigation, and plenty of room for further studies on the chemistry of benzotropones for medicinal, material, and synthetic organic chemists. Based on the progress in benzotropone chemistry including synthesis and applications summarized in this work, we feel certain that this review will find broad interest and will continue to attract much attention in organic synthesis applications. We hope that this review will facilitate the synthesis of tropolone-containing compounds discovered in nature or designed by medicinal chemists. They are also expected to be applied in new material fields due to their high functionalization capacity via their benzene ring, seven-membered ring, and carbonyl group.
